# Novel venom gene discovery in the platypus

**DOI:** 10.1186/gb-2010-11-9-r95

**Published:** 2010-09-29

**Authors:** Camilla M Whittington, Anthony T Papenfuss, Devin P Locke, Elaine R Mardis, Richard K Wilson, Sahar Abubucker, Makedonka Mitreva, Emily SW Wong, Arthur L Hsu, Philip W Kuchel, Katherine Belov, Wesley C Warren

**Affiliations:** 1 Faculty of Veterinary Science, The University of Sydney, Regimental Crescent, Camperdown, NSW 2006, Australia; 2 The Genome Center, Washington University School of Medicine, Forest Park Parkway, St Louis, Missouri 63108, USA; 3 Bioinformatics Division, The Walter and Eliza Hall Institute of Medical Research, Royal Parade, Parkville, VIC 3052, Australia; 4 School of Molecular Bioscience, The University of Sydney, Butlin Avenue, Camperdown, NSW 2006, Australia

## Abstract

**Background:**

To date, few peptides in the complex mixture of platypus venom have been identified and sequenced, in part due to the limited amounts of platypus venom available to study. We have constructed and sequenced a cDNA library from an active platypus venom gland to identify the remaining components.

**Results:**

We identified 83 novel putative platypus venom genes from 13 toxin families, which are homologous to known toxins from a wide range of vertebrates (fish, reptiles, insectivores) and invertebrates (spiders, sea anemones, starfish). A number of these are expressed in tissues other than the venom gland, and at least three of these families (those with homology to toxins from distant invertebrates) may play non-toxin roles. Thus, further functional testing is required to confirm venom activity. However, the presence of similar putative toxins in such widely divergent species provides further evidence for the hypothesis that there are certain protein families that are selected preferentially during evolution to become venom peptides. We have also used homology with known proteins to speculate on the contributions of each venom component to the symptoms of platypus envenomation.

**Conclusions:**

This study represents a step towards fully characterizing the first mammal venom transcriptome. We have found similarities between putative platypus toxins and those of a number of unrelated species, providing insight into the evolution of mammalian venom.

## Background

The venom of mammals such as shrews and the platypus (*Ornithorhynchus anatinus*) have been poorly studied to date, despite the fact that mammalian venom is extremely unusual and that toxins are useful sources for the development of novel pharmaceuticals; drugs have been developed from the venoms of many species, including various invertebrates, snakes, lizards, and insectivores (reviewed in [1-4]). However, the recently sequenced platypus genome [5] has provided a new resource for the investigation of mammalian venom and promises to vastly improve our knowledge of the contents of platypus venom, as well as to provide insight into the evolution of this unique trait.

Male platypuses possess spurs on each hind leg that are connected to paired venom glands on the dorsocaudal aspect of the abdomen to form the crural system [6]. Juvenile females are also in possession of these spurs, which regress prior to adulthood; the venom system develops only in the male. In adult males, the venom glands increase in size during the spring breeding season [7], which is to our knowledge the only such example of temporally differential venom production. The venom system is thought to have a reproductive role, such as in territory defense, although this has not been conclusively proven (reviewed in [8]). Envenomation of humans causes a number of unusual symptoms, including an immediate and excruciating pain that cannot be relieved through normal first-aid practices, including morphine, and generalized 'whole body' pain [9]. It also causes nausea, gastric pain, cold sweats and lymph node swelling [7]. Blood work reveals high erythrocyte sedimentation and low total protein and serum albumin levels, and symptoms such as localized pain and muscle wasting of the affected limb persist for weeks after envenomation [9].

Progress towards identifying the components of platypus venom has been hindered, in large part because of the limited quantities of venom available for study (reviewed in [8]). It is known that platypus venom contains 19 different peptide fractions plus non-protein components [10,11], but only three of these have been fully sequenced to date: C-type natriuretic peptides (OvCNPs) [12,13], defensin-like peptides (OvDLPs) [14,15], and nerve growth factor (OvNGF) [5]. Their functions are as yet a mystery. A venom L-to-D-peptide isomerase and hyaluronidase have also been discovered but not sequenced [10]; the venom also has protease activity [10].

Limited platypus envenomation events and a lack of testing in rodent models, as is commonly done with other venoms, have prevented the thorough understanding of the altered physiology that results from venom infusion into victims. Much of what is currently known about platypus venom has been gleaned from experiments during the 1800s, followed by proteomic studies during the 1990s. Early experiments injecting platypus venom into rabbits produced intravascular coagulation, a drop in blood pressure (probably due to vasodilation), and hemorrhagic edema [16,17]. More recent investigations also observed histamine release and cutaneous anaphylaxis [7]. *In vitro*, the venom causes smooth muscle relaxation [10,17] and feeble hemolysis [17], and when applied to cultured dorsal root ganglion cells, it produces a calcium-dependent non-specific cation current into the cells, which *in vivo *may produce nerve firing and thus pain [18]. When applied *in vitro*, OvCNP produces cation-specific ion channels [11], edema (swelling), smooth muscle relaxation and mast cell histamine release [19], and it is speculated that the OvDLPs may also produce mast cell degranulation [20].

In order to discover additional components of platypus venom, we constructed a cDNA library from an in-season adult male platypus venom gland, and have sequenced it on two independent next-generation sequencing platforms. This is the first venom transcriptome from any mammal, and so has great potential to increase our knowledge of mammalian venom. Distinguishing venom peptides from genes encoding normal body proteins (from which many venom peptides have evolved [21]) can be challenging [8] without relying on information from venoms of closely related species (of which there are none for platypuses). Here, we characterize the platypus venom transcriptome and identify putative venom genes by relying on homologies with known venom peptides in unrelated species. We also speculate on the functions of the encoded peptides in relation to the symptoms of platypus envenomation.

## Results

Two platypus venom gland cDNA libraries were sequenced using the Illumina platform, which produced 19,069,168 reads of 36 nucleotides in length, and the 454 FLX platform, which yielded 239,557 reads (average length 180 nucleotides). These reads were aligned to the platypus Ensembl genebuild (v.42). Of the 239,557 FLX sequences, 50,254 had hits to 8,821 unique cDNA sequences, of which 8,734 had amino acid translations (from the total of 24,981 cDNA sequences, 24,763 of which had amino acid translations) at 85% identity and 10^-5^. The remaining 189,303 reads that had no hits to cDNA were aligned against the assembly (535,968 sequences from Ensembl v. 42). Of these, 151,313 had hits to the assembly at 10^-5 ^and 85% identity.

A visual representation of Gene Ontology (GO) annotation of 454 read data is shown in Figure S1 in Additional file 1. The most common GO terms were cellular process, metabolic process, cell and cell part, binding, and catalytic activity; full results are available online [22]. It should be noted that GO terms such as regulation of transcription and regulation of translation, which would be required to support production and secretion of increased quantities of venom during the breeding season, appear in this list.

We identified platypus venom genes based on homology to known venom proteins. This approach was taken because we have previously found that there are homologues of all three known platypus venom peptides present in the venom of reptiles [5,23]. It has previously been speculated by us as well as other groups (for example, [21]) that there may be specific protein motifs that are preferentially selected for evolution to venom molecules independently in different animals, further supporting the use of our homology approach to identify platypus venom genes. We thus identified novel putative platypus venom genes by using TBLASTN to search the animal toxins contained within the Tox-Prot database [24] [most toxins contained within the database come from reptilians (1,204 of 2,855; v 57.8 released September 2009)] against the platypus genome, and then looked for Ensembl or GenomeScan gene predictions overlapping with 454 and Illumina reads. Sequences for peptides encoded by these putative venom genes are available online [25].

After aligning reads and Tox-Prot proteins to the platypus genome, gene prediction in regions containing both reads and Tox-Prot homologous regions yielded 155 putative genes. Predictions that did not have read support or that were expressed in three or more (of six) non-venom tissues were removed, leaving 83 putative platypus venom genes (see Additional file 1 for further details on toxin classification and Additional file 2 for peptide sequences). A threshold of three non-venom tissues was chosen so as to limit the number of false negatives; we have previously shown that platypus venom OvDLPs, OvNGF and OvCNPs are expressed in some non-venom tissues. Those genes not expressed in any non-venom tissues (33) were classified as probable (likely) platypus venom genes (Table S1 in Additional file 1).

**Figure 1:**
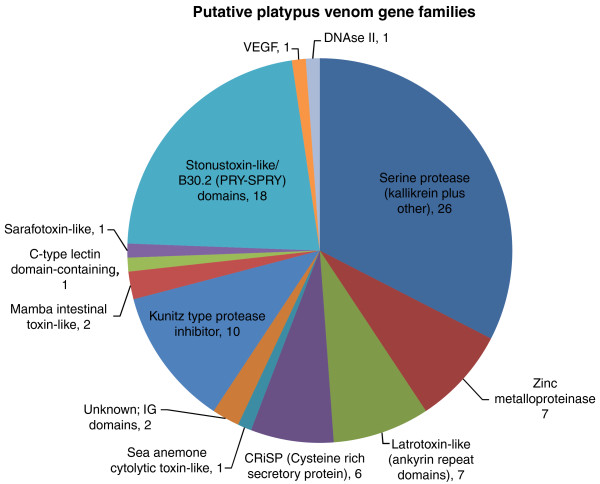
**Representation of the putative platypus venom gene families discovered by homology searching with other toxin sequences**. Putative functions are shown in Table 1.

**Figure 2:**
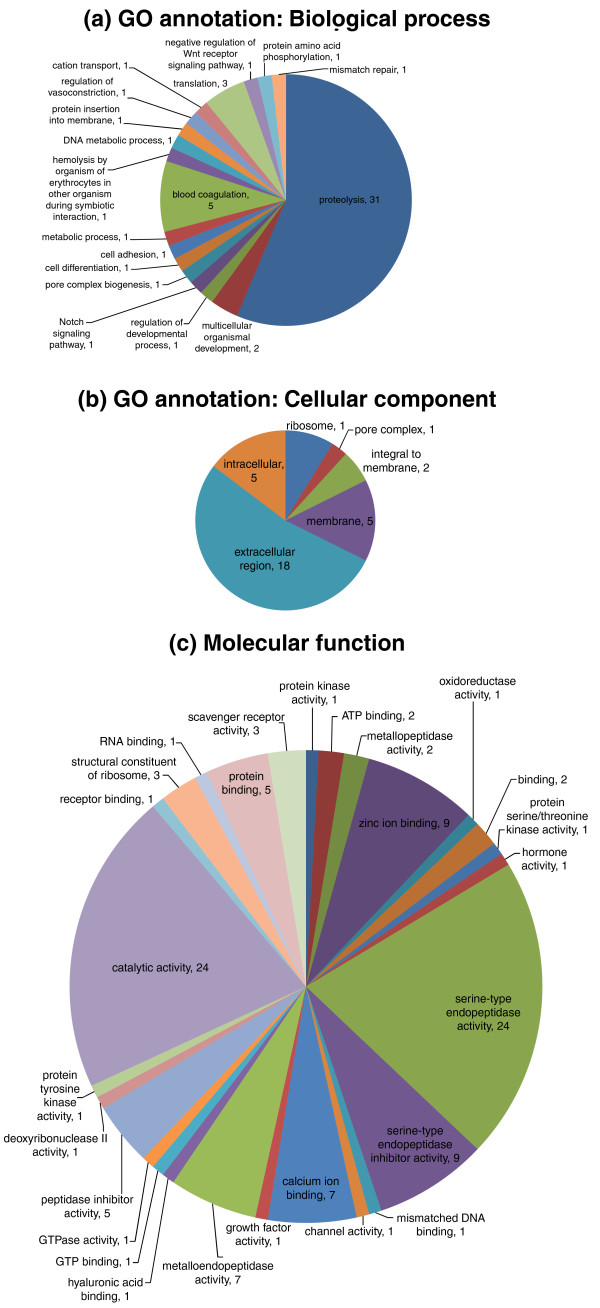
**Gene Ontology annotation of putative platypus venom genes**. **(a) **Biological process; **(b) **cellular component; **(c) **molecular function. Data can be classified under more than one GO term.

BLAST searches of GenBank and the Tox-Prot database using the peptides encoded by these genes allowed classification to toxin family (Figure 1; homology was defined using E < 0.0001) and speculation about putative functions (Table 1). The 83 putative platypus venom peptides came from 13 different families; it appears that like the venom of many snakes, platypus venom contains a large number of protein toxins from a small number of families [26], possibly because after the initial emergence of a toxin gene, subsequent duplications will increase expression levels, and thus multigene toxin families are formed [27]. GO annotation of these predicted peptides is shown in Figure 2. It can be seen that the GO term 'proteolysis' is highly represented (31 have this annotation), consistent with our analysis showing 33 protease-encoding genes. GO terms, including 'blood coagulation', 'pore complex biogenesis', 'cation transport', 'metallopeptidase activity', 'serine-type endopeptidase activity', and 'peptidase inhibitor activity', also match with the peptides encoded by the classes of venom genes that we discovered. In many cases, it was possible to link the putative functions of these peptides with the symptoms of platypus envenomation and the known pharmacological effects of the venom, which we discuss below.

**Table 1:** Previously unknown toxins identified in the platypus venom gland transcriptome data

Number of platypus venom genes	Toxin family	Range of percent identities to Tox-Prot proteins	Venom homologue examples	Predicted effects (related to envenomation symptoms)	Example references
26	Serine protease (kallikrein plus other)	27-62	Blarina toxin (shrew); gilatoxin (lizard); trocarin D (snake)	Coagulation; inflammation; nociperception; smooth muscle contraction; vasodilation	[28-30]
18	Stonustoxin-like/B30.2 (PRY-SPRY) domains	26-51	Stonustoxin (stonefish); ohanin (snake)	Hemolysis; edema; pain	[51,53,54]
10	Kunitz type protease inhibitor	44-59	Beta-bungarotoxin (snake)	Hemostatic effects; inflammation; neurotoxic; protective effects for storage	[40]
7	Zinc metalloproteinase	28-46	Zinc metalloproteinase-disintegrin (snake)	Inflammation; myonecrosis	[28,37]
7	Latrotoxin-like (ankyrin repeat domains)	25-33	Alpha-latrotoxin (spider)	Pain	[45]
6	CRiSP (Cysteine rich secretory protein)	33-68	Helothermine (lizard); cysteine-rich venom protein (snake)	Muscle wasting; smooth muscle relaxation	[46,47]
1	Sea anemone cytolytic toxin-like	36	Actinoporins (sea anemone)	Hemolysis; pain; pore formation	[48]
2	Unknown; IG domains	0	-	Unknown	-
2	Mamba intestinal toxin-like	56	MIT_1 _(snake)	Open cation channels; unknown	[72]
1	C-type lectin domain-containing	38	Rhodocytin (snake); however, contains several additional domains	Unknown (does not match envenomation symptoms)	-
1	Sarafotoxin-like	38	Sarafotoxin (snake)	Unknown (does not match envenomation symptoms)	-
1	VEGF	53	Vascular endothelial growth factor toxin (snake)	Edema; vascular permeability	[73]
1	DNAse II	35	Plancitoxin-1 (starfish)	Apoptosis; DNA degradation	[74]
Total 83					

### Proteases

Platypus venom has previously been found to have protease activity [10], and the largest group of putative platypus venom toxins identified were proteases (33 total; 12 expressed in venom gland alone are probable platypus venom toxins). These included 7 genes that had greater than 500 Illumina reads mapping to them and which therefore appear to be highly expressed. The large number of protease genes and their high expression suggests that proteases are important components of platypus venom. There are a number of hypotheses for the activities of these, discussed in the following paragraphs, but as a group they may act to cleave venom components into active molecules in the secretory cells and lumen of the venom gland or in the tissues of the victim [10]. The general protease activity could also help to dissolve tissue and facilitate the spread of the venom.

#### Serine proteases

**Figure 3:**
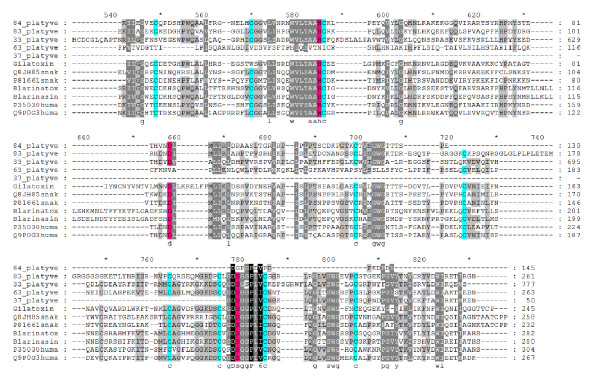
**Partial MUSCLE alignment of putative platypus venom kallikrein serine protease sequences, showing the most conserved regions**. The full alignment can be seen in Figure S5 in Additional file 1. Gilatoxin (P43685), blarina toxin (BAD18893), blarinasin (Q5FBW2), two snake sequences and two human tissue kallikreins are also shown (SWISS-PROT accession numbers are listed). The catalytic triad is highlighted in pink, and conserved cysteines highlighted in blue. Not all platypus venom peptides contain the triad and cysteines.

Twenty-six peptides were predicted from platypus venom gland cDNA to have homology to serine proteases of several types, which are found in the venom of most snakes [28]. Nine of these are expressed in venom gland alone and are classified as probable venom toxins. A phylogenetic tree of platypus serine protease sequences is shown in Figure S2 in Additional file 1. The kallikrein-type serine proteases encoded by five genes found in the platypus venom transcriptome may have effects including vasodilation, smooth muscle contraction, inflammation and nociperception (pain) (reviewed in [29]). Kallikrein-like proteases are also present in shrew [30,31], lizard [32] and some snake venoms [28]. Venom kallikreins generally possess a catalytic triad and 10 to 12 conserved cysteine residues [31,33,34]. Not all of the identified platypus peptides contain this catalytic triad (Figure 3), possibly due to problems with gene prediction, which is error-prone. However, the shrew peptides have rare non-homologous insertions near Asp of this triad [31], and non-homologous insertions are also found in lizard gilatoxin [32], indicating that some sequence variation is possible whilst still maintaining the kallikrein-like activity of the peptide.

Six of the putative platypus venom serine proteases were found to have homology to endogenous coagulation factors (for example, Factor X), which are involved in the blood coagulation cascade, and snake venom group D prothrombin activators such as trocarin D, which cause coagulation and inflammation [35]. Many other proteins encoded by genes identified in the platypus venom transcriptome also appear to have hemostatic effects (Table 1), as do many snake venoms [36]. At first glance, the symptoms of platypus envenomation do not point to hemostatic effects, but several studies have shown that the venom does in fact affect blood characteristics. Fenner *et al. *[9] recorded that an envenomated patient had a high erythrocyte sedimentation value, meaning that there were increased levels of pro-clotting factors present in the blood, which can be indicative of inflammation. The patient himself also noted that the spur wounds, despite being deep, bled little even though the platypus had to be forcibly removed. *In vitro *experiments have shown the venom to be a coagulant, and it also causes hemorrhagic edema [16,17]. We hypothesize that the putative venom serine proteases are responsible for some of these effects.

#### Metalloproteinases

**Figure 4:**
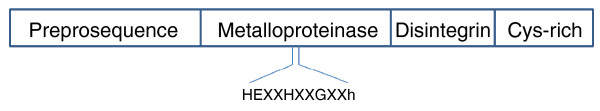
**Representation of domain order in the platypus venom metalloproteinases for which we appear to have complete sequence**. Lowercase h denotes that the residue is not found in all platypus sequences. This arrangement mirrors that of the snake venom PIII metalloproteinases (after Matsui *et al. *[28]). Domains were identified using BLAST searches of the NCBI Conserved Domains database [66].

Seven genes encoding PIII zinc metalloproteinases, which contain the zinc binding motif HEXXHXXGXXH [28], were found in the platypus venom transcriptome. Three of these were found to be expressed in venom gland alone and are classified as probable venom toxins. Zinc metalloproteinases are a second group of protease enzymes present in snake venom, which cause bleeding in the victim through fibrin(ogen)olytic activity (reviewed in [28]). This is not a known symptom of platypus envenomation. However, some snake venom metalloproteinases (including PIIIs) do not cause bleeding, and have instead been shown to cause inflammation (reviewed in [37]). We thus hypothesize that the seven metalloproteinases in platypus venom have inflammatory effects. The platypus venom peptides follow the same structure as snake venom PIII metalloproteinases, containing preprosequence, metalloproteinase, disintegrin, and cysteine-rich domains [28] (Figure 4). This conservation of domain and domain order across such widely divergent species as the platypus and reptiles again suggests the selection of certain peptide motifs for evolution to venom molecules.

### Protease inhibitors

**Figure 5:**
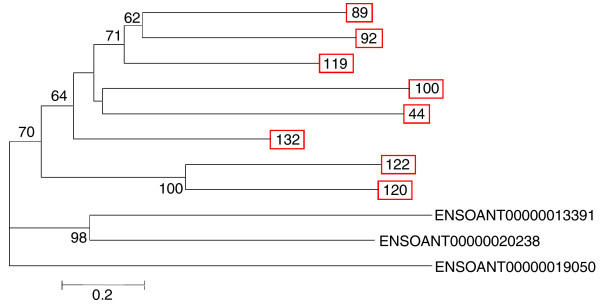
**Unrooted neighbor-joining phylogenetic tree of the kunitz domain-containing putative platypus venom peptides (boxed)**. Bootstrap values less than 50 have been omitted. ENSOANT represents platypus homologues not expressed in venom gland.

Ten putative platypus venom genes encode proteins with homology to kunitz-type protease inhibitors, many of which are involved in controlling the blood coagulation cascade [38,39]. Six of these are expressed in venom gland alone and are classified as probable platypus venom toxins. A neighbor-joining tree of putative platypus venom kunitz-type protease inhibitors plus non-venom homologues is shown in Figure 5. It can be seen that the putative platypus venom peptides cluster together into a single clade, displaying the duplications that have given rise to this putative toxin family.

Many snake venoms also contain serine protease inhibitors, which affect hemostasis and produce inflammation [40]; toxin kunitz-type protease inhibitors called kalicludines are also found in sea anemones [41]. The presence of these potential anticoagulant molecules may seem at odds with the proposed coagulation effects of some of the putative platypus venom serine proteases identified above, but there are examples in snakes where one venom contains multiple proteases with coagulant and anticoagulant effects, or where one protease has both effects; it is thought that in these cases the concentration of toxins determines the type of effect on the victim (reviewed in [28]). The function of protease inhibitors in platypus venom gland is unclear, but it is suggested that perhaps these act to inhibit the catalytic activity of proteases [29] in the venom gland, so that their effects are only released once the venom is injected into the victim. Alternatively, these inhibitors may act as neurotoxins or pro-inflammatory agents, as is the case for some of the snake venom analogues (reviewed in [42,43]). It should also be noted that in other species the non-venom protease inhibitor bikunin inhibits proteolysis and inflammation [44]. The platypus protease inhibitors thus may be expressed in the venom gland in a protective capacity to prevent inflammation in the host tissue and thus allow storage of the venom.

### Proteins homologous to invertebrate venom components: alpha-latrotoxin, CRiSPs, cytolytic toxin

Genes encoding proteins with homology to invertebrate venom toxins were also found. For example, we identified seven genes encoding peptides with homology to spider venom alpha-latrotoxin, a neurotoxin also containing ankyrin repeats, which causes a massive release of neurotransmitters on contact with vertebrate neurones (reviewed in [45]). Three of these are expressed in venom gland alone and are classified as probable platypus venom toxins. However, searches of alpha-latrotoxins against the GenBank database do reveal ankyrin repeat-containing proteins from non-venomous species at similar identities, raising the possibility that this peptide family plays a non-toxin role in the platypus venom gland. It is also possible that the homologous platypus peptides may act, like the alpha-latrotoxins, as potent neurotoxins responsible for the production of pain. Functional studies will be required to determine which hypothesis is correct.

Six genes encoding proteins with homology to CRiSPs (cysteine rich secretory proteins), which are present in a diverse range of vertebrate and invertebrate organisms, were also found. All putative platypus venom CRiSP genes were found expressed in one or more non-venom tissues, raising the possibility that they may have non-venom function. However, CRiSPs have been found in cone snail venom acting as proteases, and in snake and lizard venom acting as ion channel blockers, blockers of smooth muscle contraction (reviewed in [46]), and myotoxins [47]. The platypus CRiSPs may thus act as ion channel blockers to produce the muscle wasting observed in envenomated patients [9] and the *in vitro *effect of smooth muscle relaxation [10,17]. An analysis of the domains contained within the putative platypus venom CRiSPs is shown in Figure S3 in Additional file 1.

One protein with homology to sea anemone cytolytic toxins (for example, actinoporin) was also found. This was not found expressed in tissues other than the venom gland and on this basis is classified as a probable platypus venom toxin. This peptide has a sea anemone cytotoxic protein domain, is homologous to peptides such as hemolytic toxin and actinoporin Or-A, and does not show significant homology along its length to any proteins from other species in the National Center for Biotechnology Information (NCBI) database. Sea anemone cytotoxic proteins bind to cell membranes and have cation-selective pore-forming activity [48]; we thus suggest that the platypus homologue could cause the weak hemolysis (breaking open of red blood cells) [17] as well as pain [9] that have been observed in envenomated victims. However, actinoporin homologues have also recently been discovered in some vertebrates and plants (for example, [49]), again raising the possibility that this peptide is not a venom toxin and plays some other role in the venom gland. Functional studies will be required to confirm or refute the role of the platypus homologue in toxicity.

### Stonustoxin-like proteins

Another large group of putative platypus venom genes (18; 8 expressed in venom gland alone) were found to encode proteins with homology to stonustoxin, verrucotoxin and neoverrucotoxin (related peptides from the venom of the stonefish *Synanceja *sp. [50,51]), and snake venom ohanins. Previously, no overall sequence homology between the stonefish toxins and other proteins had been found [51]. The alpha- and beta-subunits of stonustoxin are partially homologous and share a domain (B.30.2, also known as PRY-SPRY) with other proteins that may be involved in ligand binding or protein folding [52], as well as with snake venom ohanin. All of the platypus peptides also possess SPRY, PRY, or both domains, in combination with other domains (Figure S4 in Additional file 1).

Ohanin affects the central nervous system and is proposed to cause pain and reduce locomotion for both offence and defense [53]. This effect is strikingly similar to what has been proposed as the mechanism of action for platypus venom on other platypuses [20]. Stonustoxin and neoverrucotoxin produce hypertension (high blood pressure), hemolysis, edema, and increased vascular permeability (reviewed in [51,54]), some of which are symptoms of platypus envenomation. The edema produced by stonefish envenomation is persistent (reviewed in [55]), and it is thus possible that the platypus homologues are responsible for the persistent edema that is characteristic of platypus envenomation. The fact that B.30.2-domain-containing peptides have been found in the venom of fish, reptiles, and putatively the platypus is strong support for the hypothesis that certain protein motifs have been independently selected for evolution to venom function multiple times in different lineages.

## Discussion

Our searches identified 88 putative platypus venom genes, 83 of which have not been previously identified (OvDLPs, OvNGF and OvCNPs, known to be expressed in platypus venom, were also found in the transcriptome data). It is now clear that the venom of the platypus contains a diverse range of proteins, many of which may be functional analogues of venom components of other species, including reptiles, insectivores, fish, and even invertebrates. Reptiles diverged from the vertebrate lineage 315 million years ago, and platypuses diverged from the rest of the mammals 166 million years ago [5]. The fact that these extremely divergent species share similar venom components, some of which were found repeatedly in platypus and other venoms, suggests that there are indeed protein motifs that are preferentially selected for independent evolution to venom molecules in a striking display of convergent evolution, and that many animal venoms share some similarities in their mode of action [27].

The retention of similar molecular scaffolds (with respect to protein domains and domain order) has previously been shown to occur in different proteins in snake venom [21,27,56], but this is the first time that it has been observed across such divergent organisms, including mammals, in a wide range of different molecules. It appears that in many cases the same molecular scaffolds have been repeatedly selected for in the venom of different species, with some variability in the coding region, presumably to allow toxins with slightly different activities to be derived from conserved templates [27,57]. Perhaps these similarities are to be expected when it is considered that there are only a limited number of ways that venoms can affect the homeostasis of victims to either debilitate or kill them. It is interesting to note these similarities when the assumed primary function of, say, reptile venom is to kill prey and possibly serve some digestive purpose, whilst platypus venom appears to be used for intraspecific territory defense. However, it must be noted that in many cases there are significant variations between the sequences of the putative platypus venom peptides and that of other species, so it is possible that these variations represent novel bioactivities. This feature of mutation of some regions of the protein whilst maintaining the original molecular scaffold is a key feature of the evolution of snake venom toxins [58].

To our knowledge, this is the first sequencing of a mammalian venom gland transcriptome. Although our method of identifying mammalian venom genes based on homology to previously identified toxin proteins from unrelated species will miss completely novel venom genes, there do appear to be common motifs in venom peptides across widely divergent species (reviewed in [27]), and so this represents the best approach for venom gene identification at present. In addition, the key feature of venom gene evolution by duplication and diversification from genes encoding proteins involved in normal cellular processes [21] means that rejecting a potential platypus venom gene on the basis of homology with a non-venom gene is inappropriate. For this reason, we utilized transcriptome data from additional non-venom tissues to filter our potential false positives, which we then classed as non-venom and excluded from our putative venom gene set.

In the future, emerging technologies such as improved transcriptome assemblers and longer read lengths may improve venom transcriptome sequencing projects by reducing our reliance on gene prediction methods and fragmented genome assemblies (in the case of platypus), and also allowing comprehensive transcriptomic analysis for venomous species that currently do not have a genome sequence. In addition, due to the seasonal nature of platypus venom production [7], future studies may focus on gene regulation within the venom gland as a method to refine our current predictions. This will allow the identification of those genes up-regulated during periods of high venom production, and will also represent our best chance to identify completely novel platypus venom genes with no homology to existing toxins.

## Conclusions

We have identified proteins encoded by genes expressed in the platypus venom gland that have putative involvement in processes such as hemostasis, inflammatory response, smooth muscle contraction, myonecrosis, vascular permeability and pain response. We have framed these results with respect to the known symptoms of platypus envenomation in order to gain some insight into the basic biology of this unique mammalian trait. After the completion of *in vitro *and *in vivo *assays to validate these putative venom proteins, the toxins identified here will represent a potential source of novel molecules for biomedical research. Platypus venom is a hitherto untapped resource in this respect, and this work represents our first steps towards more fully characterizing the active constituents of platypus venom.

## Materials and methods

### Platypus tissue collection and RNA extraction

Tissue was obtained opportunistically from an adult male platypus soon after death from a dog attack, and frozen at -80°C for later use. The animal died during the breeding season, and the venom glands appeared very large (approximately 3 cm in diameter), indicating that the gland was active at the time of death. Histological analysis confirmed this assessment. RNA was extracted from one venom gland using TriReagent according to the manufacturer's instructions (Molecular Research Centre Inc., Cincinnati, OH, USA). RNA samples were subjected to DNase digestion using standard protocols (Promega, Madison, WI, USA).

### Platypus venom gland cDNA synthesis

Two lots of venom gland cDNA were made, one using SuperScriptII reverse transcriptase and one using AccuScript high fidelity reverse transcriptase, in a modified SMART first-strand cDNA synthesis protocol as follows. Reagent mix one (2.0 μl 12-μM 5' Smart_Oligo (5'-AAGCAGTGGTAACAACGCATCCGACGCrGrG rG-3' ); 2.0 μl 12-μM 3' Oligo_dT_SmartIIA (5'-AAGCAGTGGTAACAACGCATCC GACTTTTTTTTTTTTTTTTTTTTTTVN-3'); 2.0 μl Invitrogen 10-mM dNTP Mix (Invitrogen, Carlsbad, CA, USA); 2.0 μl venom gland RNA; 2.0 μl diethylpyrocarbonate (DEPC)-treated water was incubated at 65°C for 5 minutes, and mixed with reagent mix two (SuperScriptII protocol: 8.0 μl SuperScriptII 5 × First-strand buffer (Invitrogen), 0.8 μl 100-mM dithiothreitol (Invitrogen), 1.0 μl 10-mg/ml BSA (New England BioLabs, Ipswich, MA, USA), 1.0 μl 40-U/μl RNaseOUT (Invitrogen), 15.2 μl DEPC-treated water, held at 45°C; AccuScript protocol: 4.0 μl AccuScript 10 × RT Buffer (Stratagene, Cedar Creek, TX, USA), 4.0 μl 100-mM dithiothreitol (Stratagene), 1.0 μl 10-mg/ml BSA (New England BioLabs), 1.0 μl 40-U/μl RNaseOUT (Invitrogen), 16.0 μl DEPC-treated water, 4.0 μl AccuScript HiFi RT (Stratagene), held at 45°C). The mixture was incubated in a thermocycler (45°C for 2 minutes (hot start); negative ramp: go to 35°C in 1 minute; 35°C for 2 minutes, 45°C for 5 minutes; positive ramp: +15°C (until 60°C) at +0.1°C/s; 55°C for 2 minutes; 60°C for 2 minutes; go to step 6 ten times) and stored at -20°C until further use.

### Library construction

Library construction used high fidelity DNA polymerase and an OligodT method following the protocols used in the platypus genome project [5]. One Illumina 36-bp library and one 454 FLX library were made. Sequencing of the 454 library produced 239,557 reads and sequencing of the Illumina library produced 19,069,168 reads (610,213,376 nucleotides from 8 flow cells). Data are available on NCBI Sequence Read Archive under the following experiment accession numbers: Illumina data [SRX026473]; 454 data [SRX000186].

### Construction of an enhanced genebuild

Tox-Prot proteins were aligned to the platypus genome using TBLASTN. All chains of high scoring segment pairs (HSPs) with E-values < 10^-5 ^were included in the analysis. Chains in unannotated regions were added to the Ensembl genebuild to create an enhanced genebuild. Chains overlapping predicted Ensembl genes were not included, and the genebuild was updated to include the Tox-Prot match.

### Analysis of 454 reads

454 reads were aligned to the platypus Ensembl transcripts (release 42) and to the Ensembl genome using BLASTN (E-value < 10^-5^). Transcripts were assigned putative function by searching against InterPro domains v.16 [59]. First, default parameters for InterProScan v.16 [60] were used to search against the InterPro database, and second, transcripts were mapped to the three organizing principles of the GO [61]. Mappings are stored by MySQL database, displayed using the Amigo browser, and are available online [22]. In this way, 7,494 transcripts were mapped to 3,280 unique Interpro domains and 5,913 sequences had GO annotation (the ontology data released in April 2008 were used in this analysis).

For each GO term, its enrichment in the venom expressed transcripts was measured over the complete set of 24,763 cDNAs (from Ensembl v.42) as background using a hypergeometric test; the *P*-value cutoff of 1.0e-5 was chosen for enrichment [62].

### Analysis of Solexa data

Illumina reads were mapped to the platypus genome (Ensembl release 49) using MAQ [63]. Reads with alignments overlapping genes in the enhanced genebuild were assigned to those genes and read abundance levels determined. Reads were also assembled using MAQ [63] and contigs in unannotated regions were extracted for further analysis.

### GO annotation of putative venom peptide predictions

GO annotation of the putative venom peptide predictions was done using InterProScan v.4.5 and the resulting data parsed using a custom script. The peptides matched 51 GO categories; peptides could be assigned more than one GO term and this resulted in 205 GO annotations in total.

### Gene prediction

Gene predictions were carried out at areas of the genome that were hit with Tox-Prot BLAST searches. Predictions were carried out on entire contigs, and 10,000 bp each side of hits to ultracontigs and chromosomes. If incomplete peptide predictions resulted from chromosomes and ultracontigs, then sequence was taken up to 100,000 bp each side in an attempt to obtain the full prediction. Predictions were carried out using GenomeScan [64], with the Tox-Prot peptide as the template. The resulting predictions were mapped to the genome on a gbrowse platform [65]. If predictions overlapped with Ensembl predictions, then the original peptide prediction was discarded and replaced with the Ensembl peptide, unless 454 FLX read data supported the GenomeScan prediction better. These peptide predictions that were not Ensembl predictions were then used in a BLASTP search of NCBI's NR database (default values) to determine the type of peptide encoded by each gene, and in some cases subjected to a Conserved Domain search [66] where the BLAST search was inconclusive (for example, where only small regions of the gene were hit). As there was similarity between some gene predictions, this was checked and redundant sequences removed (in general, this was due to non-assembly of several short contigs into longer genomic sequences). Sequences were put through a secondary screen to ensure that there was a hit from at least one Tox-Prot HSP to an exon of the gene.

### Validation of gene predictions

Screening then took place in order to eliminate any peptides found to be expressed in three or more non-venom tissues. The remaining peptide sequences were searched using TBLASTN (E = 0.0001) against the platypus EST database on NCBI (9,699 EST sequences from fibroblast cell lines). Peptides were blasted against the trimmed EST data from bill, brain, liver, spleen, and testis that were generated for the platypus genome (WUBLAST, TBLASTN, filter = seg, E = 0.0001) and alignments were manually checked to confirm expression of these genes (such as close to 100% match and spanning the entire read). Peptides were screened out if they had hits to ESTs of three out of the six different tissues. The exclusion of peptides expressed in the arbitrary value of three non-venom tissues, rather than those expressed in any non-venom tissues, was chosen because it has previously been shown that platypus venom genes are expressed in non-venom tissues [20,67]. This thus reduced the chance of excluding true venom peptides from the analysis. However, those not expressed in non-venom tissues, of which there are 33, could possibly be considered as probable/likely venom peptides; classification of these is shown in Table S1 in Additional file 1, and is also mentioned throughout the text.

All remaining predictions were checked by alignment on the gbrowse platform to assess whether there were 454 FLX hits to these coding regions. Eighty-three peptides had support from either Illumina (≥ 10 reads) or 454 reads (≥ 1 read), and were taken as putative venom peptides.

### Signal peptide analysis

The predicted peptides were run through SignalP [68], using the parameters short output; truncation 70 amino acid residues; model HMM. Nineteen of these had predicted signal peptides (classifications shown in Table S2 in Additional file 1); however, we do not believe that the absence of a signal peptide in a putative platypus venom toxin is grounds for exclusion from the category of probable venom toxin. This is due to the fact that many of these peptide predictions were truncated due to short contig lengths (due to the fragmented nature of the platypus genome assembly), and so it is expected that a higher percentage would have had detectable signal peptides if the full sequence were available.

### Homology confirmation/classification into toxin groupings

The subset of toxin peptides in the Tox-Prot database extracted as above were assembled into a BLASTable database. The peptide predictions were blasted against the Tox-Prot database (WUBLAST, BLASTP, filter = seg, E = 0.0001) to enable confirmation of toxin homology and also to allow the platypus venom peptides to be sorted into venom categories. The protein domains of some predictions were examined by BLASTing against the NCBI Conserved Domain Database [66] using default values.

### Identification of homologous platypus genes

Predictions were used in BLAST searches against the Ensembl v.56 platypus predicted peptides (WUBLAST, BLASTP, wordmask = seg, word length 3, BLOSUM80 matrix, E = 10^-15^; in cases where no hits were found E = 10^-5^).

### Phylogenetic tree construction

Trees were built from manually adjusted MUSCLE [69] alignments of peptide sequences using MEGA 4.0 [70]. Trimming to include only conserved regions took place where it is noted in figure legends. Neighbor-joining trees using pairwise deletion were constructed. In some cases, homologous animal toxins were also included in these trees; homology was identified by blasting predictions against the Tox-Prot database (WUBLAST, E = 0.0001); homologous platypus Ensembl peptide predictions not found to be expressed in the venom gland were also included. Due to the large degree of divergence between the members of many peptide groups, and the fact that similarity in many cases extended only over one or two peptide domains, sequence alignment was difficult, as noted in the Supplementary Results of Additional file 1.

### Classification of proteases

Due to the complexity of protease classification, protease predictions were categorized using BLAST against the MEROPS peptidase database [71].

## Abbreviations

bp: base pair; BSA: bovine serum albumin; CRiSP: cysteine rich secretory protein; EST: expressed sequence tag; GO: Gene Ontology; HSP: high scoring segment pair; NCBI: National Center for Biotechnology Information; OvCNP: *Ornithorhynchus *venom C-type natriuretic peptide; OvDLP: *Ornithorhynchus *venom defensin-like peptide; OvNGF: *Ornithorhynchus *venom nerve growth factor.

## Authors' contributions

CW extracted RNA, made cDNA and constructed cDNA libraries, and conducted primary data analysis, including GO annotation of predicted peptides, gene prediction, validation of gene predictions, signal peptide analysis, homology confirmation and classification into toxin groupings, identification of homologous genes, phylogenetic tree construction, and classification of proteases. CW wrote the manuscript. TP, WW and KB assisted in the design of the project, and provided assistance in finalizing the manuscript prior to publication. TP also constructed the enhanced genebuild, assisted by EW, assembled and mapped the Illumina and 454 data to the genome, assisted by AH, and provided advice on computational analysis. PK provided advice on venom pharmacology and project design. DL assisted with training, methodology and construction of the cDNA libraries. Sequencing was carried out by the Genome Center at Washington University, overseen by RW and EM. SA and MM carried out mapping of the 454 data to the genome and GO analysis of the 454 data and provided advice on computational analysis. All authors read and approved the final manuscript.

## Supplementary Material

Additional file 1 **Additional information on GO annotation of 454 data and putative platypus venom genes, location and read support for putative platypus venom genes, phylogenetic trees, and supplementary discussion**. Click here for file

Additional file 2 **Sequences of the 83 putative platypus venom peptides**. Click here for file
