# Supplementary material for: Novel venom gene discovery in the platypus
Source: Genome Biol. 2010 Sep 29;11(9):R95. doi: 10.1186/gb-2010-11-9-r95 (PMC2965387; doi:10.1186/gb-2010-11-9-r95)
Supplement: Additional file 1 — Additional information on GO annotation of 454 data and putative platypus venom genes, location and read support for putative platypus venom genes, phylogenetic trees, and supplementary discussion. [file gb-2010-11-9-r95-S1.PDF]

Additional File 1. Additional information on GO annotation of 454 data and putative platypus venom genes, location and read support for putative platypus venom genes, phylogenetic trees, and supplementary discussion.

GO annotation of 454 data

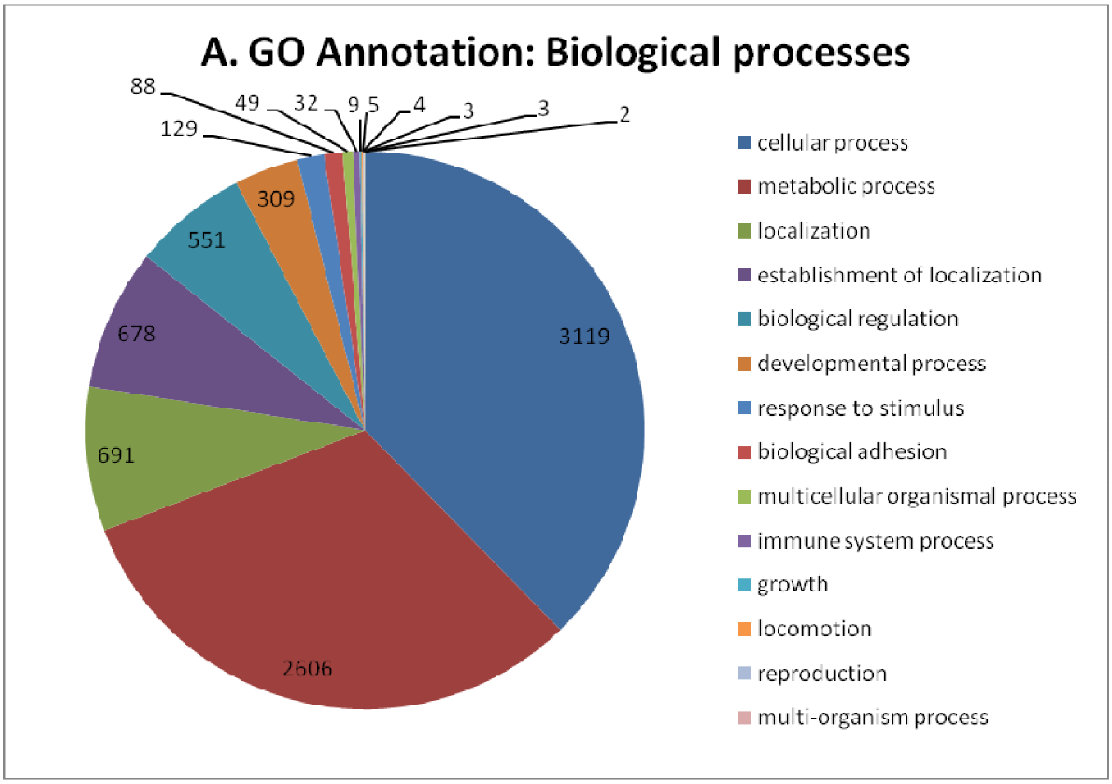

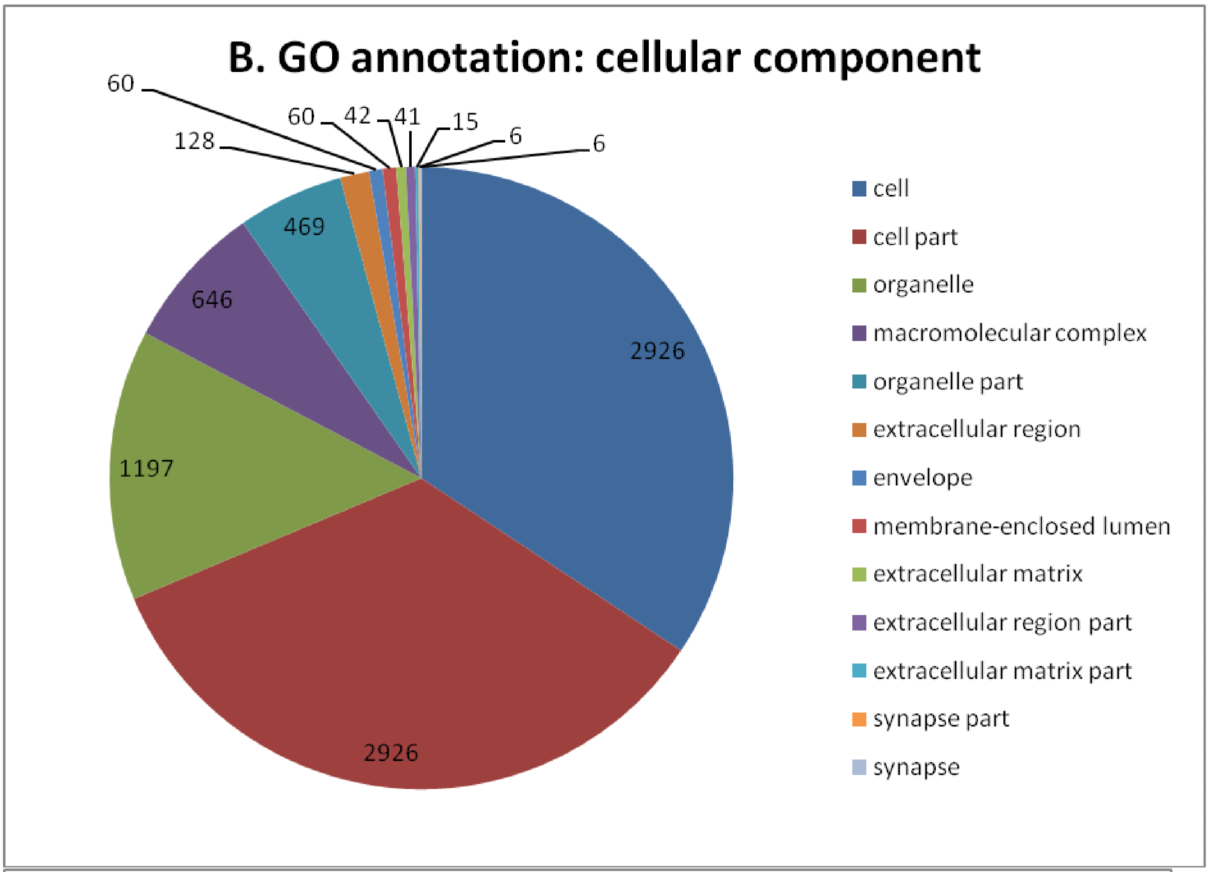

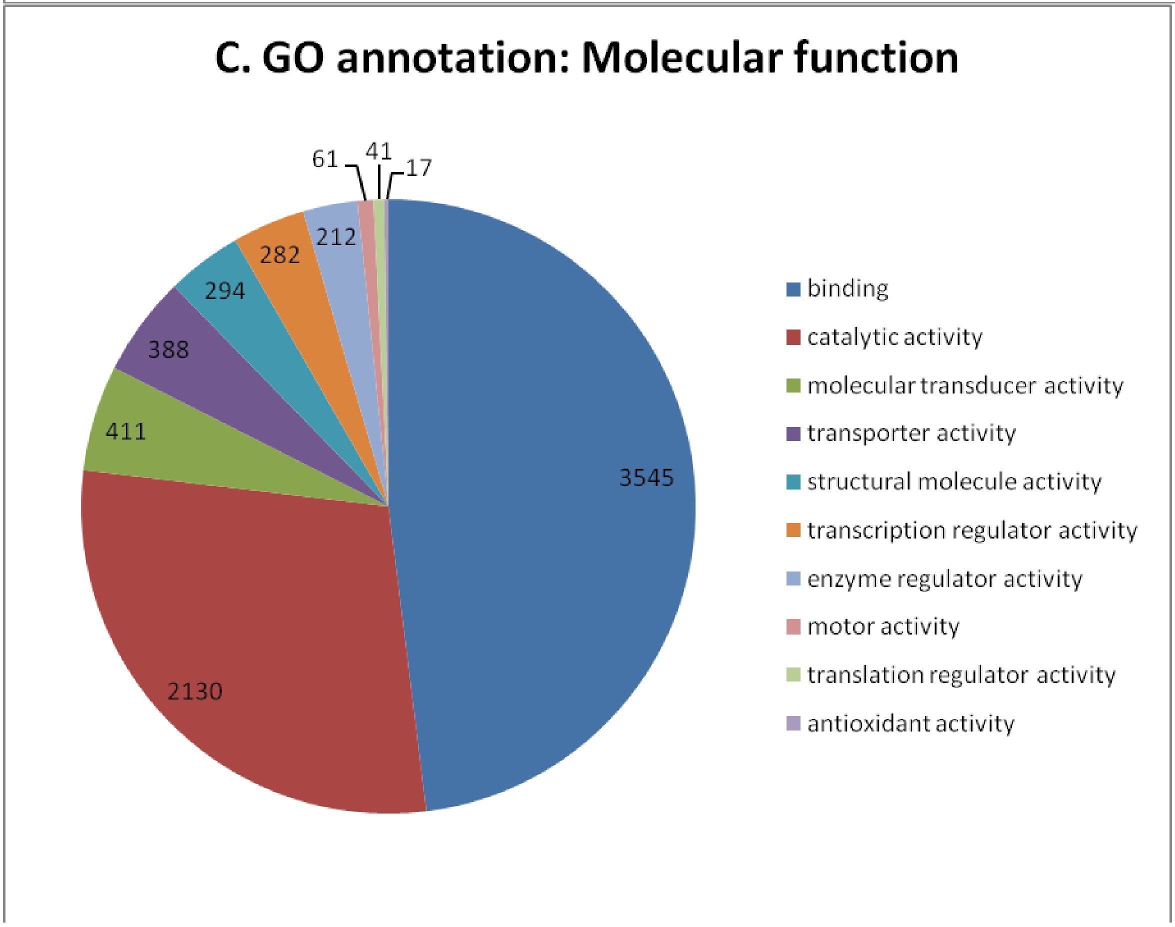

Figure S 1. GO annotation of 454 read data by terms A. Biological processes B. Cellular component and C. Molecular function. Data can be classified under more than one GO term. See Supplementary

Material for methods; full results including GO category IDs can be found at http://www.nematode.net/cgi-bin/amigo/go_platypus_venom/go.cgi.

Results for putative platypus venom genes

Table S 1. Classification of the 33 platypus venom peptides not expressed in any non-venom tissues.

# Platypus venom genes	Toxin family
9	Serine protease (kallikrein plus other)
8	Stonustoxin-like/B30.2 (PRY-SPRY) domains
6	Kunitz type protease inhibitor
3	Zinc metalloproteinase
3	Latrotoxin-like (ankyrin repeat domains)
0	CRiSP (Cysteine rich secretory protein)
1	Sea anemone cytolytic toxin-like
1	Unknown; IG domains
0	Mamba intestinal toxin-like
0	C-type lectin domain-containing
1	Sarafotoxin-like
1	VEGF
0	DNAse II
33	TOTAL

Table S 2. Classification of the 19 platypus venom peptides with signal peptide as predicted by SignalP.

# Platypus venom genes	Toxin family
7	Serine protease (kallikrein plus other)
3	Stonustoxin-like/B30.2 (PRY-SPRY) domains
3	Kunitz type protease inhibitor
1	Zinc metalloproteinase
0	Latrotoxin-like (ankyrin repeat domains)
0	CRiSP (Cysteine rich secretory protein)
0	Sea anemone cytolytic toxin-like
1	Unknown; IG domains
1	Mamba intestinal toxin-like
1	C-type lectin domain-containing
1	Sarafotoxin-like
0	VEGF
1	DNAse II
19	TOTAL

Table S 3. GO term IDs for GO annotation of the putative platypus venom peptides.

Cellular Component	GO ID	Total gene count
ribosome	GO:0005840	3
pore complex	GO:0046930	1

integral to membrane	GO:0016021	2
membrane	GO:0016020	5
extracellular region	GO:0005576	18
intracellular	GO:0005622	5
**Molecular function**	**GO ID**	**Total gene count**
protein kinase activity	GO:0004672	1
ATP binding	GO:0005524	2
metallopeptidase activity	GO:0008237	2
zinc ion binding	GO:0008270	9
oxidoreductase activity	GO:0016491	1
binding	GO:0005488	2
protein serine/threonine kinase activity	GO:0004674	1
hormone activity	GO:0005179	1
serine-type endopeptidase activity	GO:0004252	24
serine-type endopeptidase inhibitor activity	GO:0004867	9
mismatched DNA binding	GO:0030983	1
channel activity	GO:0015267	1
calcium ion binding	GO:0005509	7
growth factor activity	GO:0008083	1
metalloendopeptidase activity	GO:0004222	7
hyaluronic acid binding	GO:0005540	1
GTP binding	GO:0005525	1
GTPase activity	GO:0003924	1
peptidase inhibitor activity	GO:0030414	5
deoxyribonuclease II activity	GO:0004531	1
protein tyrosine kinase activity	GO:0004713	1
catalytic activity	GO:0003824	24
receptor binding	GO:0005102	1
structural constituent of ribosome	GO:0003735	3
RNA binding	GO:0003723	1
protein binding	GO:0005515	5
scavenger receptor activity	GO:0005044	3
**Biological process**	**GO ID**	**Total gene count**
proteolysis	GO:0006508	31
multicellular organismal development	GO:0007275	2
regulation of developmental process	GO:0050793	1
Notch signaling pathway	GO:0007219	1
pore complex biogenesis	GO:0046931	1
cell differentiation	GO:0030154	1
cell adhesion	GO:0007155	1
metabolic process	GO:0008152	1
blood coagulation	GO:0007596	5

hemolysis by organism of erythrocytes in other organism during symbiotic interaction	GO:0052331	1
DNA metabolic process	GO:0006259	1
protein insertion into membrane	GO:0051205	1
regulation of vasoconstriction	GO:0019229	1
cation transport	GO:0006812	1
translation	GO:0006412	3
negative regulation of Wnt receptor signaling pathway	GO:0030178	1
protein amino acid phosphorylation	GO:0006468	1
mismatch repair	GO:0006298	1
		205

Table S 4. Location, classification, and read abundance data for putative platypus venom genes. Highest Tox-Prot hit e-values are shown, along with highest non-platypus hits when BLASTed against the GenBank nr database. Peptide sequences in fasta format can be found in Appendix S1.

ID Number	Location	Genomic co-ordinates (start-end)	Name	Classification by BLASTP against Tox-Prot	Illumina read count	454 reads	Highest Tox-Prot hit	Highest non-platypus hit to GenBank nr database	%identities to toxprot
94	Contig4484	347-29569	94_ENSOANT00000019231	CRISP	63	Not evaluated	8.00E-27	2.00E-80	33
29	Contig11593	2275-18411	29_CRVP_TRIST	CRISP	42		3.80E-20	3.00E-21	54
98	Contig51030	2399-1488	98_HELO_HELHO	CRISP	5260		4.20E-21	1.00E-14	54
136	X1	2650590-2649887	136_HELO_HELHO	CRISP	969		4.20E-21	2.00E-14	54
49	Contig191645	535-392	49_CRVP2_LAPHA	CRISP	2739		2.50E-16	7.00E-15	65
107	Contig83239	1070-1165	107_HELO_HELHO	CRISP	464		6.70E-13	3.00E-06	68
1	1	9974337-9960449	1_ENSOANT00000023257	C-type lectin	14		3.00E-17	0	38
154	Contig2134	1874-4963	154_ANF39_ORNAN	C-type natriuretic peptide	95666		5.50E-19	1.00E-28	100
151	Contig4716	31589-32717	151_OvDLPB	Defensin-like peptide	4273		1.40E-29	No non-platypus hits	100
152	Contig4716	35169-36201	152_OvDLPC	Defensin-like peptide	293		2.60E-28	No non-platypus hits	100
19	7	18865879-18944635	19_ENSOANT00000018893	Kunitz-type protease inhibitor	158		6.90E-12	0.00E+00	44
44	Contig18043	6679-16184	44_ENSOANT00000017425	Kunitz-type protease inhibitor	8	Y	4.60E-15	2.00E-60	50
92	Contig4011	32045-05667	92_ ENSOANT00000008336	Kunitz-type protease inhibitor	33	Not evaluated	2.10E-17	7.00E-92	50
132	Ultra523	78475-80783	132_IVBTI_OXYSC	Kunitz-type protease inhibitor	2550		7.50E-15	1.00E-17	50
100	Contig5613	3104-20945	100_KC3_ANESU	Kunitz-type protease inhibitor	339		1.30E-13	8.00E-18	55
106	Contig6878	15555-38384	106_IVBTI_OXYSC	Kunitz-type protease inhibitor	559		7.10E-16	4.00E-157	56
120	Ultra42	1508056-1520702	120_KC1_ANESU	Kunitz-type protease inhibitor	354		1.90E-16	5.00E-15	56
89	Contig39003	5240-00454	89_ENSOANT00000011622	Kunitz-type protease inhibitor	16		1.40E-15	7.00E-62	57
119	Ultra42	1499736-1502192	119_ENSOANT00000004654	Kunitz-type protease inhibitor	64		6.50E-16	3.00E-41	58
122	Ultra42	1528313-1561237	122_KC1_ANESU	Kunitz-type protease inhibitor	1636		1.70E-15	8.00E-23	59
27	Contig1091	70478-108783	27_ENSOANT00000004000	Latrotoxin	179		3.20E-09	0.00E+00	25
116	Ultra362	993119-1033694	116_LATA_LATMA	Latrotoxin	182		1.60E-40	0.00E+00	27
118	Ultra393	55738-93479	118_ENSOANT00000002372	Latrotoxin	598		6.90E-44	0.00E+00	28
143	ultra222	3159423-3189139	143_ENSOANT00000010286	Latrotoxin	0		3.10E-25	0.00E+00	28
2	2	1798507-1832761	2_ENSOANT00000010958	Latrotoxin	249		1.60E-13	0	30
73	Contig26760	658-5160	73_ENSOANT00000020565	Latrotoxin	5		5.50E-08	1.00E-36	32

113	Ultra29	2963416-2964961	113_ENSOANT00000016497	Latrotoxin	34		1.10E-12	1.00E-104	33
6	3	55430963-55432817	6_ENSOANT00000011197	Neurotoxic peptide	48		5.90E-09	7.00E-82	34
32	Contig1161	180227-195225	32_ENSOANT00000024943	no hits	1	Y	No hits	6.00E-110	0
96	Contig4728	182-23935	96_ENSOANT00000015877	no hits	11	Not evaluated	No hits	3.00E-89	none
62	Contig20697	15034-17258	62_ENSOANT00000011287	Plancitoxin (DNAse)	748		4.50E-56	1.00E-116	35
131	Ultra474	4285133-4303733	131_SRTDL_ATRMM	Sarafotoxin-like	2015		8.40E-10	4.00E-76	38
38	Contig15824	1797-6136	38_ACTP1_ACTVL	Sea anemone cytolytic toxin-like	60		5.40E-08	2.00E-14	36
35	Contig13194	6510-25844	35_ENSOANT00000023374	Stonustoxin-like peptide	70		4.70E-05	1.00E-72	30
112	Ultra288	3521679-3511591	112_ENSOANT00000012771	Stonustoxin-like peptide	63		3.00E-14	1.00E-69	31
47	Contig18410	594-10078	47_ENSOANT00000017858	Stonustoxin-like peptide	381		1.00E-12	1.00E-98	32
51	Contig19556	1561-24671	51_ENSOANT00000019691	Stonustoxin-like peptide	163		2.90E-11	1.00E-104	32
48	Contig191147	95-631	48_VESP_LACMU	Stonustoxin-like peptide	108		1.50E-18	1.00E-54	33
103	Contig58910	230-3571	103_VESP_LACMU	Stonustoxin-like peptide	151		1.50E-18	4.00E-55	33
59	Contig20531	127-15150	59_ENSOANT00000011909	Stonustoxin-like peptide	26		1.40E-18	2.00E-98	34
60	Contig2061	3200-8452	60_VESP_OPHHA	Stonustoxin-like peptide	350		6.80E-17	3.00E-40	34
102	Contig5740	16832-26945	102_ENSOANT00000000934	Stonustoxin-like peptide	55		5.10E-21	5.00E-99	34
108	Contig93671	1158-0502	108_ENSOANT00000004798	Stonustoxin-like peptide	41		5.70E-17	8.00E-44	34
127	Ultra462	5676783-5670064	127_ENSOANT00000004184	Stonustoxin-like peptide	319		1.10E-13	0.00E+00	35
93	Contig4432	426-16529	93_VESP_LACMU	Stonustoxin-like peptide	437		4.50E-11	6.00E-17	36
130	Ultra474	1380893-1371508	130_ENSOANT00000016139	Stonustoxin-like peptide	29		5.10E-16	2.00E-105	36
66	Contig24569	11380-05189	66_VESP_OPHHA	Stonustoxin-like peptide	2	Y	1.60E-22	8.00E-42	39
101	Contig5740	165-14083	101_VESP_LACMU	Stonustoxin-like peptide	10	Not evaluated	9.10E-07	7.00E-42	40
117	Ultra369	564118-555525	117_ENSOANT00000023178	Stonustoxin-like peptide	310		5.80E-13	0.00E+00	40
50	Contig19251	1314-5396	50_VESP_LACMU	Stonustoxin-like peptide	419		5.30E-14	1.00E-51	51
133	Ultra543	141462-143451	133_ENSOANT00000006376	Toxin Mit1	15		3.80E-27	3.00E-34	56
134	Ultra67	353818-348115	134_ENSOANT00000006275	VEGF	0	Y	4.70E-29	2.00E-79	53
155	7	34930447-34931184	155_OvNGF	Venom nerve growth factor	14782	Not evaluated	1.00E-81	4.00E-107	63

3	3	10781118-10772456	3_ENSOANT00000011763	Venom serine protease	49		1.20E-13	0	27
71	Contig26215	6983-10587	71_ENSOANT00000002247	Venom serine protease	346		2.40E-29	6.00E-71	28
125	Ultra445	937870-951999	125_ENSOANT00000005815	Venom serine protease	541		4.10E-21	7.00E-154	29
7	4	22189061-22197523	7_ENSOANT00000017207	Venom serine protease	22		1.40E-22	1.00E-51	31
88	Contig37386	3629-5277	88_ENSOANT00000019002	Venom serine protease	23		1.00E-24	4.00E-47	32
8	5	6061000-6073066	8_ENSOANT00000001117	Venom serine protease	311		9.40E-37	6.00E-180	33
24	Contig10377	16350-28712	24_FA10V_TROCA	Venom serine protease	307		1.40E-28	3.00E-102	33
33	Contig1209	16194-06590	33_ENSOANT00000021475	Venom serine protease	17		6.00E-35	0	33
72	Contig26595	68650-4149	72_ENSOANT00000021872	Venom serine protease	102		3.00E-20	7.00E-38	33
80	Contig3488	19880-42747	80_ENSOANT00000000762	Stonustoxin	74		1.40E-20	2.00E-63	33
64	Contig23476	13977-09127	64_ENSOANT00000020676	Venom serine protease	590		2.00E-30	1.00E-56	34
28	Contig11214	10090-22311	28_ENSOANT00000015601	Venom serine protease	24		6.20E-27	2.00E-83	35
126	Ultra445	6549300-6546822	126_ENSOANT00000023530	Venom serine protease	1785		3.60E-32	0.00E+00	36
46	Contig18167	13057-16574	46_ENSOANT00000020334	Venom serine protease	530		9.60E-38	1.00E-92	37
61	Contig20666	8210-12292	61_VSPP_CERCE	Venom serine protease	50		8.30E-16	6.00E-28	38
83	Contig35121	6189-01515	83_ENSOANT00000017802	Venom serine protease	138		8.70E-41	4.00E-70	38
111	Ultra222	3043009-3025562	111_BLTX_BLABR	Venom serine protease	870		2.50E-22	0.00E+00	38
142	ultra490	740536-761316	142_ENSOANT00000021035	Venom serine protease	0	Y	3.90E-39	0.00E+00	38
16	6	6138514-6143234	16_ENSOANT00000018475	Venom serine protease	19	Not evaluated	5.10E-96	0.00E+00	43
84	Contig35392	5943-11703	84_ENSOANT00000023650	Venom serine protease	285		6.90E-25	1.00E-45	45
105	Contig67430	3323-0626	105_ENSOANT00000030925	Venom serine protease	14		1.10E-12	2.00E-21	45
43	Contig177471	315-449	43_VSP10_TRIST	Venom serine protease	57		2.50E-09	8.00E-12	50
115	Ultra336	9683503-9669526	115_ENSOANT00000032408	Venom serine protease	70		7.40E-127	0.00E+00	50
63	Contig22292	530-2221	63_BLTX_BLABR	Venom serine protease	12659		5.30E-30	1.00E-33	59
37	Contig147239	798-982	37_VSPL_BOTAS	Venom serine protease	1270		1.00E-17	1.00E-18	62
115B	Ultra336	9635431-9646795	115B_ENSOANT00000024652	Venom serine protease (coagulation factor)	124		4.90E-50	4.00E-123	33
124	Ultra437	491706-481972	124_LC2_VIPLE	Venom serine protease (coagulation factor)	13		1.20E-08	7.00E-16	37

56	Contig20439	8764-03838	56_ENSOANT00000007184	Zinc metalloproteinase	102		1.00E-63	0.00E+00	28
42	Contig17606	12030-08473	42_ENSOANT00000008735	Zinc metalloproteinase	36		1.70E-55	5.00E-170	31
57	Contig20439	11891-10428	57_VMED_AGKCL	Zinc metalloproteinase	0	Y	9.20E-61	6.00E-152	35
15	5	9427586-9417798	15_ENSOANT00000012758	Zinc metalloproteinase	195	Not evaluated	4.50E-96	1.00E-160	41
14	5	9393832-9379983	14_ENSOANT00000012761	Zinc metalloproteinase	97		3.40E-129	0.00E+00	42
10	5	9333772-9351397	10_ENSOANT00000012763	Zinc metalloproteinase	223		8.00E-153	0	46
99	Contig5337	29594-22518	99_ENSOANT00000014702	Zinc metalloproteinase	46		1.60E-89	0.00E+00	46

Supplementary Results: Phylogenetic trees

Venom serine proteases

The snake venom serine proteases, as well as the homologue blarinatoxin, appear to contain an TrypSPC domain. On the basis that domain conservation is important for venom function, one peptide was removed, and any obviously truncated sequences were removed from the alignment to provide common sites for tree building.

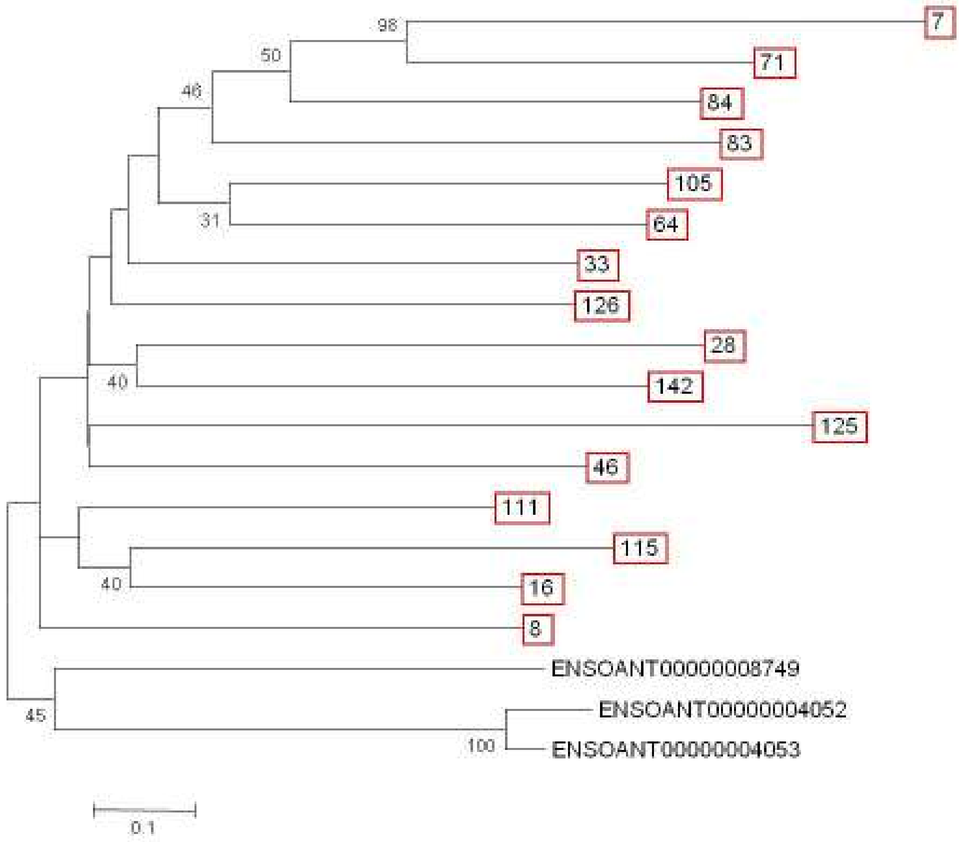

Figure S 2. Rooted phylogenetic tree of the putative platypus venom serine proteases (boxed). Bootstrap values less than 50 have been omitted. ENSOANT represent platypus homologues not expressed in venom gland.

CRiSPs

It became apparent during alignment analysis that the sequences for the putative platypus venom CRiSPs were in many cases incomplete, due to the short genomic contigs that these sequences were predicted from. In this case, we opted for a domain-based analysis, as it is apparent that domains are important for protein activity, but it is difficult to perform phylogenetic analysis with any certainty because of the incomplete gene predictions.

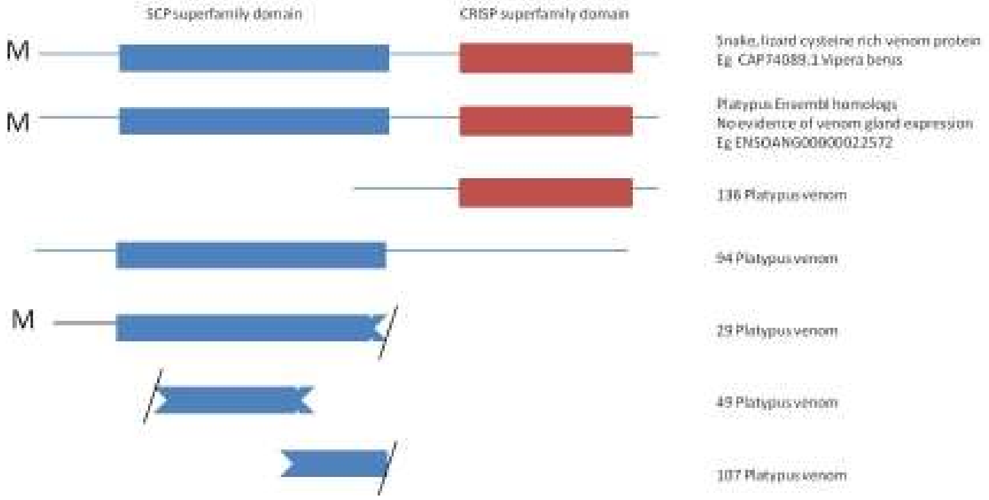

Figure S 3. Diagrammatic representation of conserved domains in the five CRiSPs expressed in platypus venom gland, as determined by BLASTing against the NCBI Conserved Domain Database (http://www.ncbi.nlm.nih.gov/Structure/cdd/wrpsb.cgi). M indicates the starting methionine in the peptide sequence. Slashes represent obviously incomplete peptide predictions (amino acid residues denoted as X in the peptide prediction), and v-shaped domain ends indicate incomplete domains. It can be seen that the putative platypus venom CRiSPs are likely incomplete sequences, although they display domain conservation with the reptile peptides of the CRiSP family as well as platypus homologues, one of which likely gave rise to the putative venom peptides.

Stonustoxin-like peptides

On the basis that specific peptide domains are necessary for venom function, two putative platypus venom stonustoxin-like peptides missing both of the SPRY/PRY domains that are present in snake and stonefish venom homologues were removed from the putative platypus venom gene list.

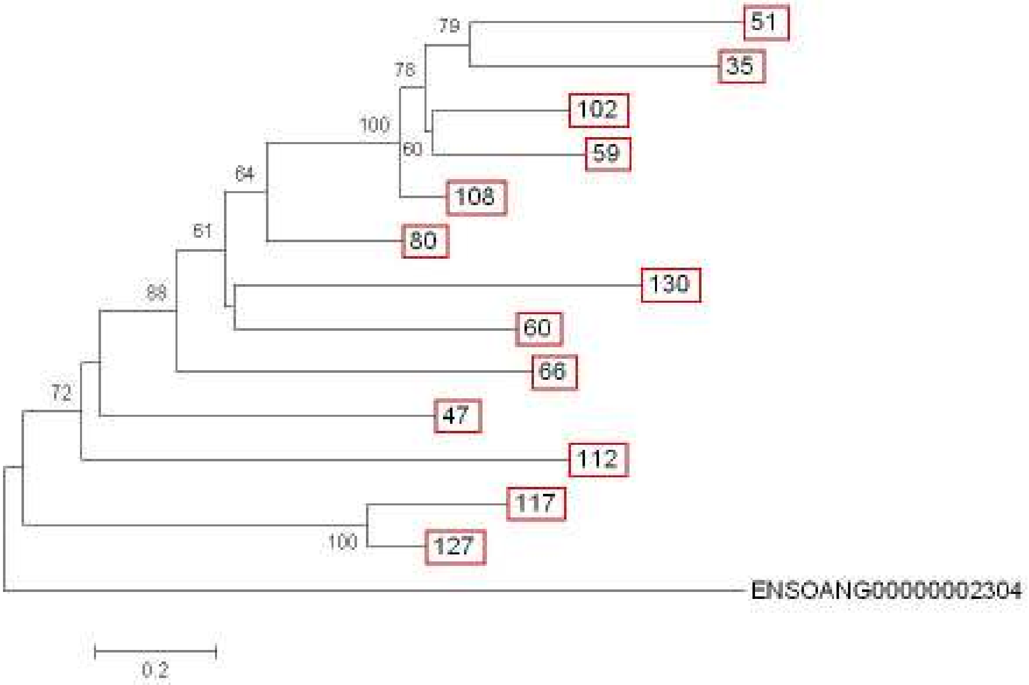

Figure S 4. Unrooted phylogenetic tree of the stonustoxin-like putative platypus venom peptides (boxed). Bootstrap values less than 50 have been omitted. ENSOANG represents platypus homologues not expressed in venom gland.

Kallikrein-like peptides

```
                      *        20         *        40         *        60         *        80         *       100
84_platyve : --------------------------------------------------------------------------------------------------------- :   -
83_platyve : --------------------------------------------------------------------------------------------------------- :   -
33_platyve : DNPQRKRDYLCLAPLWLALVTLAAAGVLIWYFLGFKEEGTSSRLYSGSVAVLDRQFFPDLANHESGAFRSEIAKAQIMLKELISATRLSAYYNSSTVYSFGAKPL : 105
63_platyve : --------------------------------------------------------------------------------------------------------- :   -
37_platyve : --------------------------------------------------------------------------------------------------------- :   -
Gilatoxin  : --------------------------------------------------------------------------------------------------------- :   -
Q8JH85snak : --------------------------------------------------------------------------------------------------------- :   -
P81661snak : --------------------------------------------------------------------------------------------------------- :   -
Blarinatox : --------------------------------------------------------------------------------------------------------- :   -
Blarinasin : --------------------------------------------------------------------------------------------------------- :   -
P35030huma : --------------------------------------------------------------------------------------------------------- :   -
Q9P0G3huma : --------------------------------------------------------------------------------------------------------- :   -

                 *       120         *       140         *       160         *       180         *       200         *
84_platyve : --------------------------------------------------------------------------------------------------------- :   -
83_platyve : --------------------------------------------------------------------------------------------------------- :   -
33_platyve : TCFFWFILQVPNSKVQKMSPDWVKEVLVDELKARANASDALPQDDQYEMDPGTLTLLEASLRDIIVLNSTLGMLCCSVSFSLLGAGEGRGVMGELGLGEEDLYRL : 210
63_platyve : --------------------------------------------------------------------------------------------------------- :   -
37_platyve : --------------------------------------------------------------------------------------------------------- :   -
Gilatoxin  : --------------------------------------------------------------------------------------------------------- :   -
Q8JH85snak : --------------------------------------------------------------------------------------------------------- :   -
P81661snak : --------------------------------------------------------------------------------------------------------- :   -
Blarinatox : --------------------------------------------------------------------------------------------------------- :   -
Blarinasin : --------------------------------------------------------------------------------------------------------- :   -
P35030huma : --------------------------------------------------------------------------------------------------------- :   -
Q9P0G3huma : --------------------------------------------------------------------------------------------------------- :   -

                    220         *       240         *       260         *       280         *       300         *
84_platyve : --------------------------------------------------------------------------------------------------------- :   -
83_platyve : --------------------------------------------------------------------------------------------------------- :   -
33_platyve : PQAQKLRESKRERQWGQLEERRGNGGARSILGDCRGRRERLILLLYGCSRVEPALDVLSSGPVMSVVWKKGLYSYDDPFTLAAQAVPFQVCAVNLTLEESLELQG : 315
63_platyve : --------------------------------------------------------------------------------------------------------- :   -
37_platyve : --------------------------------------------------------------------------------------------------------- :   -
Gilatoxin  : --------------------------------------------------------------------------------------------------------- :   -
Q8JH85snak : --------------------------------------------------------------------------------------------------------- :   -
P81661snak : --------------------------------------------------------------------------------------------------------- :   -
Blarinatox : --------------------------------------------------------------------------------------------------------- :   -
Blarinasin : --------------------------------------------------------------------------------------------------------- :   -
P35030huma : --------------------------------------------------------------------------------------------------------- :   -
Q9P0G3huma : --------------------------------------------------------------------------------------------------------- :   -

               320         *       340         *       360         *       380         *       400         *       420
84_platyve : --------------------------------------------------------------------------------------------------------- :   -
83_platyve : ------------------------------------------------------------MALFIL--------------------------------------- :   6
33_platyve : PIRTPYYPSYYSPSTHCTWHLKVPSPDYGVALWFDSYALQRGKKSGLCTQGQWTIQNRRMCGQRILNAYAERIPVVTAAGLTINFTSQISLTGPGLQAHYSLYNT : 420
63_platyve : ----------------------------------------------------------AHISSCLQ--------------------------------------- :   8
37_platyve : --------------------------------------------------------------------------------------------------------- :   -
Gilatoxin  : --------------------------------------------------------------------------------------------------------- :   -
Q8JH85snak : --------------------------------------------------------------------------------------------------------- :   -
P81661snak : --------------------------------------------------------------------------------------------------------- :   -
Blarinatox : --------------------------------------------------------------------------------------------------------- :   -
Blarinasin : --------------------------------------------------------------YLLL--------------------------------------- :   4
P35030huma : ---------------MCGPDDRCPARWPGPGRAVKCGKGLAAARPGRVERGGAQRGGAGLELHPLL--------------------------------------- :  51
Q9P0G3huma : ------------------------------------------------------------MSLRVL--------------------------------------- :   6

                      *       440         *       460         *       480         *       500         *       520
84_platyve : --------------------------------------------------------------------------------------------------------- :   -
83_platyve : -----------------------------------------------------------------------------A-------------------LTLVAAAA :  15
33_platyve : SDPCPGAFLCPLNGLCVPGCDGIKDCGSGMDERNCVCPAKFQCPEDSACIALPKVCDRHLDCVDGSDEQHCNHTVPCGAFTFKCADGSCVKKPNPQCDDLPDCPD : 525
63_platyve : -----------------------------------------------------------------------------DVSTWMSARHLKLNMPKTELLIFPPKPC :  36
37_platyve : --------------------------------------------------------------------------------------------------------- :   -
Gilatoxin  : --------------------------------------------------------------------------------------------------------- :   -
Q8JH85snak : ---------------------------------------------------------------------------------------MVLIRVLANLVMLHLSYG :  18
P81661snak : --------------------------------------------------------------------------------------------------------- :   -
Blarinatox : ----------------------------------------------------------------------------------MCFLLLCLTLTLAGTGAVPTGPS :  23
Blarinasin : ---------------------------------------------------------------------------------------LCLPLTLMGTGAVPPGPS :  22
P35030huma : -----------------------------------------------------------------------------GGRTWRAAR----DADGCEALGTVAVPF :  75
Q9P0G3huma : -----------------------------------------------------------------------------GSGTWPSAPKMFLLLTALQVLAIAMTQS :  34

                 *       540         *       560         *       580         *       600         *       620         *
84_platyve : ----------------KIIEGVECQPDSHPWQAALFRG--NELHCGGVLVNRNWVLTAAHCKL-------PEYQVYLGQHNLKAKEKGGQVIRARTSYRHPNYST :  80
83_platyve : ATDK-----------EKIIAGEKCKEDGHPYQVAVYRG--GGLLCGGVLIHPGWVLTAAHCRK-------PQLQVLLGKYNLHKQEEFQQLSPVAQPFPHPDYDR : 100
33_platyve : QSDELHCDCGLQAPTNRILGGFNSVEGEWPWQASLQAQ--GRHICGGSLIADRWVLSAAHCFQKDSLALPAVWTVYLGKLQQNSSRASEVSFKVSRLLLHPYYEE : 628
63_platyve : PLPD-----------FPVTVDGTTI-------LPISQARNLGDIVDSVFSFTPHIQSVTNICR-------SHLHNIAKIHPFLSIQ-TAIVLSILHSTARIIFLQ : 115
37_platyve : --------------------------------------------------------------------------------------------------------- :   -
Gilatoxin  : -----------------IIGGQECDETGHPWLALLHRS--EGSTWSGVLINRDWILTAAHCEE------LGPMKICFGMKNRNVLRGDEQVKVAAVKKCYPATAG :  80
Q8JH85snak : EKSS-----------ELVIGGRPCNINQHRSLALLYNS--SGFLCGGTLINQQWVLSAAHCDM-------ENMQIYLGLHNFSLPNMDQKRRVAEEKFFCLSNKS : 103
P81661snak : -----------------VIGGDECDINEHPFLAFMYYS--PQYFCGMTLINQEWVLTAAHCDK-------TYMRIYLGIHTRSVANDDEVIRYPKEKFICPNKKK :  79
Blarinatox : IEIH-----------SRIIGGWECDKHSQPWQALLTFTRKHNSVCGGVLVHSQWVLTAAHCIG-------DNYKVLLGLHDRSSEESTVQEARVSARFPHPLYNM : 110
Blarinasin : IEIH-----------PRIVGGWECDKHSQPWQALLTFTNGLDGVCGGVLVHPQWVLTAAHCIG-------DNYKIKLGLHDRFSKDDPFQEFQVSASFPHPSYNM : 109
P35030huma : -DDD-----------DKIVGGYTCEENSLPYQVSLNSG---SHFCGGSLISEQWVVSAAHCYK-------TRIQVRLGEHNIKVLEGNEQFINAAKIIRHPKYNR : 158
Q9P0G3huma : QEDE-----------NKIIGGHTCTRSSQPWQAALLAGPRRRFLCGGALLSGQWVITAAHCGR-------PILQVALGKHNLRRWEATQQVLRVVRQVTHPNYNS : 121
                                 g                           l    w   aahc                g

                    640         *       660         *       680         *       700         *       720         *
84_platyve : E----------------------THVNDI-------MLIRIDRAASLTGRIRP--IPIPTSCDKPGTKCTVSGWGTTTS--------PE---------------- : 130
83_platyve : G----------------------RHDNDI-------MLIRIAHPTPVSRHIKP--ISIETDCNANSSSCLISGWGKTDR-EGQYP--GRGGKCKPSQNRSGLGLP : 171
33_platyve : D----------------------THDYDV-------ALIQIDHPVVRSPVVRPLCIPAPTHFFEPGLKCWVTGWGA-LR-EGGSF--SNTLQKVDVQIVH----- : 695
63_platyve : K----------------------CFKNVA-------PLLKNLQWLPVDLRMKQKLITIGFKAVHPVAPSYLSSLLSFYC-LPPIF--PSELQCVELQILP----- : 183
37_platyve : --------------------------------------------------------------------------------------------------------- :   -
Gilatoxin  : T------------IYNCNYVNTVLMNNDILKRELFPMLIKIDSSVDYNERVAP--ISIPTSPASLGAECSVLGWGTTTP-DDVTL--PDVPVCVNIEIFN----- : 163
Q8JH85snak : Y---------------------TKWDKDI-------MLIKINRRVKTSTHIAP--ISIPSNPPRLRSVCRIMGWGSITS-PRETL--PYVPHCANIMILR----- : 170
P81661snak : N---------------------VITDKDI-------MLIRINRPVKNSTHIAP--ISIPSNPPSVGSVCRIMGWGAITT-SEDTY--PDVPHCANINLFN----- : 146
Blarinatox : TLLNLLLSHKMNLTFFYKTFLGADFSHDI-------MLIRIDQPVQLTDAVQV--IDIPTQEPQVGSTCHVSGWGRTSQNYENSFVLPEKLQCVEFTLLS----- : 201
Blarinasin : RLLKLLLSDELNDTYYDEISLGADFSHDI-------MMMQIEKPVQLNDAVQV--IDIPTQEPQVGSKCHASGWGSMDP-YSRNFPRTGKLQCVDLTLMS----- : 199
P35030huma : D----------------------TLDNDI-------MLIKISSPAVINARVST--ISIPTTPPAAGTECLISGWGNTLS-FGADY--PDELKCLDAPVLT----- : 224
Q9P0G3huma : R----------------------THDNDI-------MLIQIQQPARIGRAVRP--IEVTQACASPGTSCRVSGWGTISS-PIARY--PASLQCVNINISP----- : 187
                                        d            l                           c   gwg

               740         *       760         *       780         *       800         *       820         *
84_platyve : ------------------------------------------DGPGPDVPD---------------------PKDPDI------------------------- : 145
83_platyve : LPLETEMGRGSGSGKETLYNRISR-NVPCQRSEQMGRDPCLGDSGGPIVCGGR-----LRGLVSWGEVPCGTGEKPGVYTNVCRYVDWIRETIRGN------- : 261
33_platyve : ----------QDLCDEAYRFSITP-RMMCAGYRKGKKDSCQGDSGSPIVCKEPSGRWFLAGLVSWG-LGCGRPNYFGVYTRISRVLDWIKQEMS--------- : 777
63_platyve : ----------NEICDLAHPEKVTE-FMLCAGLMQGGKDSCQGDSGGPIICNNT-----LQGITSWGHFPCGLPGKPGLSTKVFAYLDWIAKTMEDN------- : 263
37_platyve : -----------------------------------------GDSGGPIVCNGK-----LQGLVSWGTFPCGQPNDPGVYTQVCKFTAWIRKTMKEN------- :  50
Gilatoxin  : ----------NAVCQVARDLWKFT-NKLCAGVDFGGKDSCKGDSGGPIVCDNQ-----LTGNVSWG-FNCEQGEKYG-YIKLIKFNFWIQNIIQGGTTCP--- : 245
Q8JH85snak : ----------YWVCRAIYGSLPAKSRTLCAGVPRRRIGSCLGDSGGPLICNGQ-----IQGIASWGSDPCVNHGAPGVYTKVFDYNDWIQSIIAGNTAATCPP : 258
P81661snak : ----------NTVCREAYNGLPAK--TLCAGVLQGGIDTCGGDSGGPLICNGQ-----FQGILSWGSDPCAEPRKPAFYTKVFDYLPWIQSIIAGNKTATCPP : 232
Blarinatox : ----------NNECSHAHMFKVTE-AMLCAGHMEGGKDSCVGDSGGPLICDGV-----FQGIASWGSSPCGQQGRPGIYVKVFLYISWIQETIKAHS------ : 282
Blarinasin : ----------NNECSRSHIFKITD-DMLCAGHIKGRKDTCGGDSGGPLICDGV-----FQGTTSWGSYPCGKPRTPGVYVKIFSHVDWIREIIATHS------ : 280
P35030huma : ----------QAECKASYPGKITN-SMFCVGFLEGGKDSCQRDSGGPVVCNGQ-----LQGVVSWG-HGCAWKNRPGVYTKVYNYVDWIKDTIAANS------ : 304
Q9P0G3huma : ----------DEVCQKAYPRTITP-GMVCAGVPQGGKDSCQGDSGGPLVCRGQ-----LQGLVSWGMERCALPGYPGVYTNLCKYRSWIEETMRDK------- : 267
                                         c          c gDsggP 6c          g  swg   c     pg y        wi
```

Figure S 5. MUSCLE alignment of platypus venom kallikrein sequences. Gilatoxin (P43685), blarina toxin (BAD18893), blarinasin (Q5FBW2), two snake sequences and two human tissue kallikreins are also shown (SWISS-PROT accession numbers shown). The catalytic triad is highlighted in pink, and conserved cysteines highlighted in blue. Not all platypus venom peptides contain the triad and cysteines.

Kunitz-type protease inhibitors

It was evident upon looking at the domains of the putative platypus kunitz-type protease inhibitors that they followed a slightly different structure to their snake venom homologues whilst still conserving one of the same domains. Snake venom peptides appeared to have kunitz domains, whilst the platypus predictions contained kunitz domains, plus WAP and other varying domains. This demonstrates the preferential selection of particular protein motifs for evolution to venom peptides. The tree below contains the includes WAP-containing (122 and 120, which group together) and kunitz only domain-containing peptides, but not those with the extra mixed domains; including these made tree construction impossible as they lacked common sites. No non-platypus venom sequences were included, as the phylogenetic distance appeared to be too great to allow meaningful alignment.

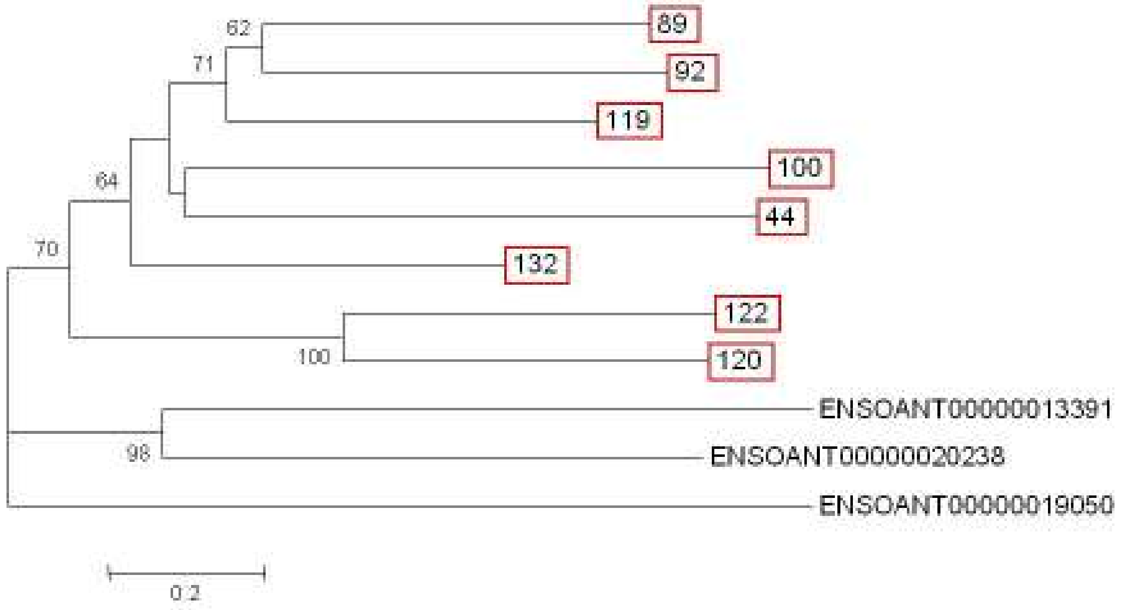

Figure S 6. Unrooted phylogenetic tree of the kunitz-domain containing putative platypus venom peptides (boxed). Bootstrap values less than 50 have been omitted. ENSOANT represent platypus homologues not expressed in venom gland.

Zinc Metalloproteinases

Although all obviously truncated sequences were removed prior to alignment, we were unable to obtain a monophyletic venom clade in this tree. However, it is apparent that one of the two venom clades contains sequences from genes located on chromosome 5. Basal to this clade is a peptide not found expressed in venom gland, and the gene encoding this is also located on chromosome 5. The four genes in this clade (see Figure S8) lie within a ~150kb stretch of chromosome 5, with regular ~ 20kb gaps in between each gene. We suggest that the zinc metalloproteinase sequences evolved as follows: duplications giving rise to the basal non-venom genes, followed by a translocation of ENSOANT00000012756 to chromosome 5,

followed by a duplication and neofunctionalization (to form '10') and then subsequent rounds of gene duplication (to give '14' and '15'), with the same process occurring for genes in the original location. Thus, all putative zinc metalloproteinases are derived from a common ancestor, with duplications occurring in separate areas of the genome giving rise to the putative platypus venom zinc metalloproteases.

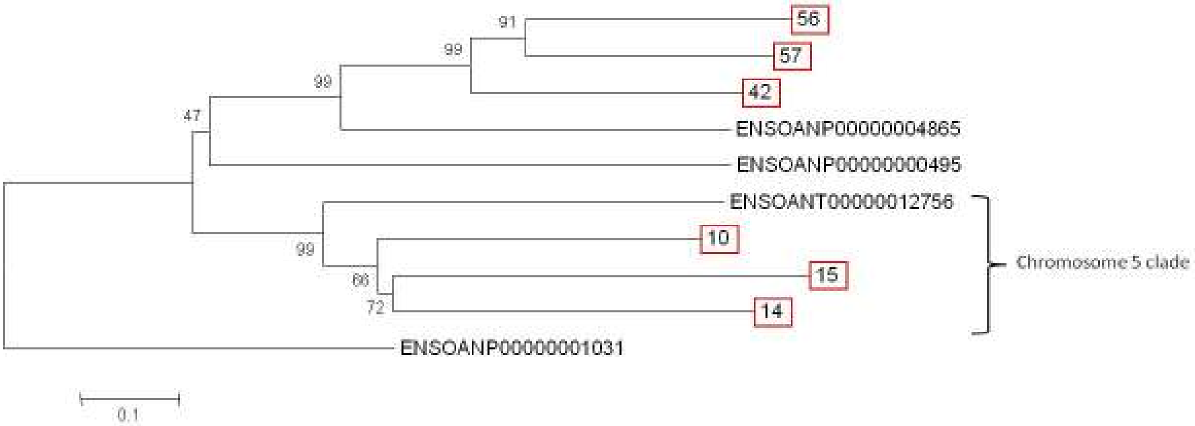

Figure S 7. Rooted phylogenetic tree of the zinc metalloproteinase putative platypus venom peptides (boxed). ENSOANT represent platypus homologues not expressed in venom gland.

C-type lectin

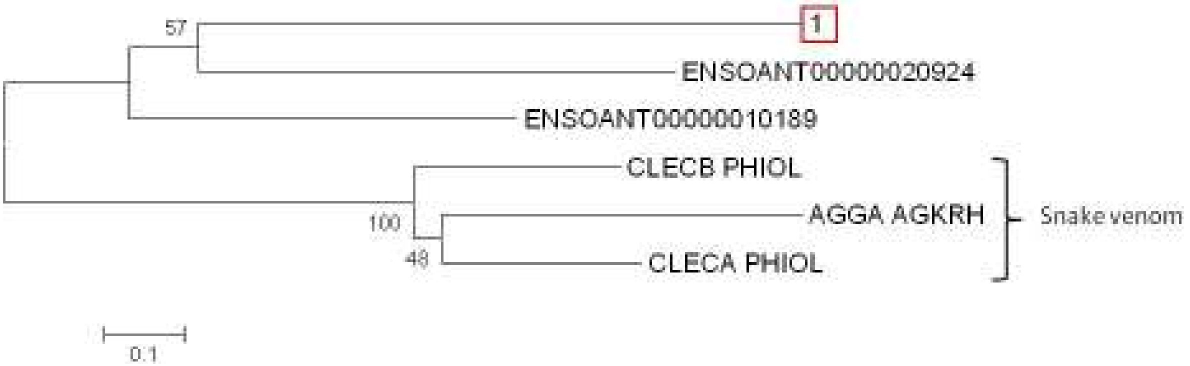

Figure S 8. Unrooted phylogenetic tree showing gene duplication leading to the platypus c-type lectin putative venom peptide (boxed). ENSOANT represent platypus homologues not expressed in venom gland. Snake venom peptides cluster separately.

Supplementary Discussion

Serine Proteases

Proteases are generally divided into two classes based on their structures: serine proteases and metalloproteinases [reviewed in 28]. 26 peptides were predicted from platypus venom gland cDNA sequence as having homology to serine proteases, which are themselves divided into several families; 24 of these were categorised by searching against the MEROPS peptidase database [71] as S1A serine proteases. Serine proteases are found in most snake venoms, and some have fibrinolytic and fibrinogenolytic activity and thus disrupt coagulation, whilst others, such as the kallikreins, cause bradykinin release, or target molecules such as proteins involved in the coagulation cascade [reviewed in 28]. The serine

protease genes expressed in platypus venom gland were determined via BLAST searches to show homology to serine protease families including the kallikreins, coagulation factors, and group D prothrombin inhibitors.

The kallikrein family is a large multigene family of varying biological functions that is present in a wide range of tissues, including salivary gland and skin [reviewed in 33]. Many kallikreins may play a role as activation enzymes [33], and although it is not a diagnostic feature, some convert kininogen to the vasoactive peptide Lys-bradykinin, which has a variety of effects including vasodilation, smooth muscle contraction, inflammation and nociperception [reviewed in 29]. All of these effects are features of platypus envenomation or have been observed in pharmacological studies. Shrew Blarina toxin, a kallkrein-like peptide, is toxic and causes vasodilation and inflammation by converting kininogens to kinin [30], whereas the related kallikrein-like peptide blarinasin is not toxic to mice and probably acts like an endogenous kallikrein [31]. It is anticipated that future functional studies will show that some of the identified platypus venom kallikrein-like proteases may also act as normal tissue kallikreins whilst others will have toxic effects.

Metalloproteinases

All snake venom metalloproteinases are zinc metalloproteinases; this also appears to be the case with the platypus venom metalloproteinases, which contain the zinc binding motif HEXXHXXGXXH (bar one, which is missing the final H) [28]. BLAST searches of the MEROPS database [71] revealed all seven platypus venom metalloproteinases to be members of the high molecular mass PIII family. Due to the fragmented nature of the platypus genome, many of our venom gene predictions appear to be truncated. However, the platypus venom metalloproteinases for which we appear to have full-length sequences follow the same structure as snake venom PIII metalloproteinases, containing preprosequence, metalloproteinase, disintegrin, and cysteine-rich domains [28] (Figure 5).

Protease inhibitors

Ten putative platypus venom genes encode proteins with homology to kunitz-type protease inhibitors, many of which are involved in modulating coagulation pathways by binding to and inactivating Factor X and Tissue Factor VII/VIIa complexes in the blood coagulation cascade [in 38, 39]. The kunitz-type protease inhibitors in platypus venom all contain varying numbers of kunitz domains in combination with other domains. However, two peptides containing only two kunitz-type protease inhibitor domains each are notable for this similarity with bikunin, another two kunitz-type protease inhibitor domain protein. Bikunin is mostly produced in the liver, and inhibits proteolysis and inflammation, possibly by interacting with immune cells such as neutrophils to prevent chemokine production [44]. The platypus bikunin-like molecules may be expressed in the venom gland in a protective capacity to prevent inflammation in the host tissue and thus allow storage of the venom.
