# Supplementary material for: Novel venom gene discovery in the platypus
Source: Genome Biol. 2010 Sep 29;11(9):R95. doi: 10.1186/gb-2010-11-9-r95 (PMC2965387; doi:10.1186/gb-2010-11-9-r95)
Supplement: Additional file 2 — Sequences of the 83 putative platypus venom peptides. [file gb-2010-11-9-r95-S2.PDF]

**Additional File 2. Sequences of the 83 putative platypus venom peptides.**

>1_ENSOANT00000023257 peptide:ENSOANP00000023253 pep:NOVEL_protein_coding
MLLNIKSILWICSTLIVTNALHKVQVEKSPPVKGSLSGKVSLPCHFSTMPTLPPSYNNTSEFLRIKWSKVEEDKI
GKDLKETTVLVAQNGNIKIGQGYKGRVSVPTHPEDVGDASLTVVKLRASDAGFYRCDVMYGIEDTQDTVSLAVEG
VVFHYRASSSRYTLNFKEAQEVCLANGAVIASPEQLKAAYEDGFEQCDAGWLSDQTVRYPIRTPRAGCYGDMMGK
EGVRTYGFRVPDERYDVYCYVDHLEGEVFHITSPNKLTFEEAEEECVNQDGRLATVGELHAAWRNGFDKCNYGWL
ADGSVRHPVTVARAQCGGGLLGVRTLYRFENQTGFPAPDSTFDAYCHKPKQNISETTTIELSIPVETGSPNLSKE
SQIIPVKATLNPHLVTDTQTNVPPMGMIHEQPQEHTSQTQTATDSTAAESPSVAGSTGGPWQLEPTSSASGPLET
PARPDGGEDIFWTPAVPEGDRPGSLPVGSHHIPDQGVTPTEQIEVGPWVTSLRATVHTPVKESLATGTSVTMTGE
EMVQSATKMPLEAKTESKLKTHVISEERPTGHFGFTVGQDDDSESLRTVWPDQSTLVPTQVPEFVTVSKTSEESN
VTQRESAEVTEISPVTTLESEEPLTEDTQPWPEGSIHQHTSLAPPSENHTEEKLFFSSPESTSPQKLVEGTATAI
FRSQQTEGSSQIEEQTAGLEEITISHVLPTSTAWLPTVEREDGTQDSFSGTAEGFPGTDAFPKQIYVTEYTGEMT
KHYDVPVSEITDQTSTSVESTEAKRHKEESTMPAVAFETDTTHSTRSTLDLEVITVSKWSLEERNVTISESVESL
EPFNVTELPTAVLTIAGVSEKAEQTTGLAEHTVKSFTPSPDSVESPVVKFTEEPWSSEEKRKASFQSTTKAAVKE
ESIVTDSITEEKLRPLPGTEQPIDEPVERSTIFHELDLKAPRAVTTLHPYEETSDMEGSAFHYNGDAQKPGSVTQ
AIPISEITDFKWDVIGPSVTLGGETAKDKLLTEQTALEATISPGAPSTTITEDAEREPSFSSLATLPSTTLQASE
KVEKQDTEGSAYPTSSGDLGIDGKVPDFEEIPIVKGTTLGTTVHEAISPTDVVTEPSRTLRITPSSRPQVHISPV
TEQELKREDSRSVPPVSPSRSSTTDSITQPQWETSTETKAETFPQESETGSGWLERPHFEHSPNETTVSGSTTGP
LDTVQPTSVSERFTTKEILPAVDEEPGDKTTSDIVIIDESIPPSVPTTPDELTTKEEGIEIDREYFTTSAASVVR
PTGLRKTEESTEALDRQEVSTSKPQTGKKSHPDIIVHVFEVTRNKTGRLSDLSVFGQPIDSESKEEEPCSEVPDE
EHDLLSEILPVLPEIIDLEDFFSEEDEDCENATDVTTSPTLLTTSPTLQFIPEKQQLTTAPKDTKAEEVRRPFLE
SVAPQNFSQNGGINPFVLAETGAPGIILPGESKELVEPFEATWKPEIYSETTEPFSSGEPDIYPTEPLNAFANLY
QSRPSEVAESLTQQGPGFHKISGESTKTLTLFSEESSGDAPVDQESLRTVIPVTTEVGTGKDVGKTLAITSTPST
ATSYATGDSLPEDGSSVLTDDIQPETLSTVESWEKTTPGHVVEVLRSSSVLIPEGSGYLEETIIQASTVATDLLD
SGTLDSQSATKVTSDFSNILLTESVSNISATLIPLREETAYPSGEPTATTSRSAGEMDSSNQVFNTFPETKAESS
KREITSNSVPSIKYLSTQESGQLLVTELKNSTDRINSIHLEPTTARSILPSTESTEPKTEEINSAFLSTEQNLLW
LFREYTETKSNTVQDFVQGNPTTSAISLDLSFGDQGSGGIDIATEPTSVTSIPIKGVTGSKEVKTDHEEKEVAST
EPSSVKYTLSPKPTTVILSTESDISLSKTEIVSRTPKEVLSTVEPEEPNHGTELSGAQTIFPMEEGSSDDLGKYL
IAPTTHSITEEEKGIDGGSGDAPFLERGTQRAPFDSATVPTFKRINPTTDSKGPESSTVASVLRLLAETFIVEGS
GEEAPDLSRTLELVPEGPTEKATKIDSPLVEQGSGEVDSLIDTKTTIPPQTLPDVTRGQSAKGGEEIINTDSSDY
SLTAEPTKLVVNTGHEIKVEILDSENQVPSEEKIQELSEPEKTTIATGEIIFELETFAKMTSRSTESPLLTTKET
SISRKESSLGEEDISFISPITPELEEKVGTFTYLTRFTTKPSPSSHPLSATESMKSESVGPNPAGWFMKEEESIT
QNILVTPQGTKPTAKELNADLLFSGQGSGEEIIPSQPTLDALATSNFINIKDIVSTLKPELSELESSAPNLLVEK
IEQNEIEENEGSGLLGPEAEETSEADTLEHITSTMVDGSRGIGAQLLSTPVTLSLDPAAPTSQTVSRDETKPPTS
EMITERVSQGYTLQSETKGTIAFSSLSGTLSPSTDRIFITSTSKESSALYESSGETSGWFDPEDLEHSTQTAKTE
TTRDVPSSHEENFSEVSPSEAVVTNGSFPALVISPSHSEDSNRYGSTDGSSDGSSDGSSDGSTDGSLDGSSDGST
DNSVDAPTDNSTYSSADASTDKEEKDEIFPITESAVLEILPEITSDKNTIIDLDSTKSTDEDILGIQTETDVEVH
TTPDGNNEEILEVEDNYEQAFNNSLAEEKAEGSGDNLPMTMPNQAISNESEISQGIRRVDHTEYDSGNIMHFTTK
VSKLHTETKLEVSLPTVASLALLSTSPSVQQEIKDPDFETKHVATESNLDDNFESSTLSDSQAVADQSDAISTSG
YLDRTPEENEDKKFVNPSFKPELSSGEGEELIDQSPDVSISSTKLVFQSSTELPHATERSTPTVYTETTLADSAS
EKLYLVSSQTSKSPVTGHLVNVASEQPGITVSITDSREPNLSPEQTLKEVHVDTVATFKPPGEELFHQTESPSWG
TLNAVSESSTVESKPGFVEQVSEGREEAESSASEPTMELEMKTLVDSITTTLPKLSKETTDKESLLVVTTKDGAY
IKIVGENDEEKIHSGPHLTSTSPSNEILAGVLLHPGQPTTEIPTLPYSTLDINHKSPTGFVVQKDSTTYTTATKK
PVAASNNEFSNQATLNTEQLNTDHVTSSLSPLEASNGTEFLLVNNGDSVEGTAVYIPGLNLCKTSPCLNGGTCYE
RDSSYVCTCLPGFSGDQCEYDFDECQSNPCRNGATCVDGINTFTCLCLPSYVGALCEQDTETCDYGWHKFQGQCY
KYFAHRRTWDAAERECRLQGAHLTSILSHEEQLFVNRVGHDYQWIGLNDKMFEHDFRWTDGSTLQYENWRPNQPD
SFFSAGEDCVVIIWHENGQWNDVPCNYHLTYTCKKGTVACGQPPVVENAKTFGKMKPRYEINSLIRYHCKDGFIQ
RHLPTIRCLGNGRWALPKITCLNPSANQRTYSKKHFKNSSSAKDNSINSPKHYHPWISRWQDSRR
>2_ENSOANG00000006878:ENSOANT00000010958 peptide: ENSOANP00000010956
pep:NOVEL_protein_coding
MSLVDLGKKLLEAARAGQDDEVRILMANGAPFTTDWLGTSPLHLAAQYGHYSTTEVLLRAGVSRDARTKVDRTPL
HMAASEGHASIVEVLLKHGADVNAKDMLKMTALHWATEHNHQEVVELLIKYGADVHTQSKFCKTALDISIDNGNE
DLAEILQIAMQNQINTNPESPDTVTIHAATPQFIIGPGGVVNLTGLVSSEHSSKTTDETGVSAVQFGNSSTSVLA
TLAALAEASAPLSNSSDTPVVATEEVVTAESVDGAIQQVVSSGGQQVITIVTDGIQLGNLHSIPTSGIGQPIIVT
MPDGQQVLTVPATDIAEETVISEEPPVKRQCIEIIENRVESAEIEEREALQKQLDEANREAQKYRQQLLKKEQEA
EAYRQKLEAMTRLQTNKEAV
>3_ENSOANT00000011763 peptide:ENSOANP00000011761 pep:NOVEL_protein_coding
MQNLAPSCPSSVTTHIFLVVMNKEPANDGEWSGKQPICIKACREPKISDLVRQRVLPMQVQSRQTPLHQLYSSAF
SKQKLESYPTKKPALPFGDLPPGYQHLHTQLQYECISPFYRRLGSSRRTCLKTGKWSGRAPSCIPICGKTENITE
PKAPGIRWPWQAAIYRRTSGLQGGGSLHKGMWILICSGALVNERTVVVAAHCVTDLGKVTVIKTAELKVVLGKFY

RDEDRDEKTIQNLRISAIIVHSNYDPILLDSDIAVVKLLDKARISNRVQPICLVAPRDLSPSAEESQIAITGWKI
ITDVKDPGLKNDTLRSGVVRIVDSLRCEQQYEDNGIQVSITDNMFCARQDTTFPSNICPAETGGIAAVSLPGKVS
PELRWHLMGLVSWGYDKTCNLDLFTAFTKVVPFKDWIEKNMK
>6_ENSOANG00000007031:ENSOANT00000011197 peptide:ENSOANP00000011195
pep:NOVEL_protein_coding
RGKLGPVAPPRPAMPTRLLLALALALGHLSPLTVATSPSGSASGPPVHSNAIKIAPPVAPGAPVAPLAPLPGGTA
ASPAPPKHQPLDARKPGSPRCAEDGHCPPDEFCAGPGGSHLCQACRKRRKRCLRHAMCCPGNVCSNGICVPSDHD
HFQQPEIDETVIESFTRDHSTLDVHSKRTTLSSKMYHSKGQESSVCLRSSDCAAGLCCARHFWSKICKPVLKEGQ
VCTKHRRKGSHGLEIFQRCYCGEGLSCRIQKDHHLASNSSRLHTCQRQ
>7_ENSOANG00000010859:ENSOANT00000017207 peptide:ENSOANP00000017204
pep:NOVEL_protein_coding
SSKLCGEFEEQNEGGLLSSRCWPPKVKTINITLGAHNIEKKEETQQHMILLRSVTHPEYKKNPPTNDIMLLQLEK
KAKVTKAVRPLKLPRSLVKLKPGMVCSVAGWGGNLQSKVQPILQEVKLKVMGDEVCTSCYPRNFKNKTQICAGDP
RQYKSSYQGDSGGPLVCGKVAEGIVSYGNKNGSPPRVFTRISSYLSWIKTTMNSP
>8_ENSOANG00000000713:ENSOANT00000001118 peptide: ENSOANP00000001117
pep:NOVEL_protein_coding
KPGAMSGKLLRFWLLWGVVSSVSMQERIRLFRRGARIAPVTCMDYTTKKVYQQRESWLRHRGAQLEYCRCDSGLA
RCHTVPGKACSQQMCYNGGTCYQALYFSDFICSCPSGFDGKQCEINVNAMCYSDLGLTYRGTWSVTDSGADCLNW
NSSALTQKKFSGRRPDAIKLGLGNHNYCRNPDKDSRPWCYVFRKGEYTWEFCSTPLCTQATCGKRQKSPPQYRIK
GGLYTDIAAHPWQAIILAWSRKLFKDHFLCGGVLIDSCWVLSAAHCFEERYEPSRITVVLGRTFREASGENEQVL
KVEKYTVHENFDPDTYDNDIVLLKLKSDHCAKETDNVHPVCLPEKGLQLPDWTECELSGYGKHEESSPFYSERLK
EAHVRLYPANRCSSRFLSNRVITDNMLCAGDTRSGGTNGNLDDACQGDSGGPLVCMKDNRMHLLGIISWGIGCGQ
KDIPGIYTKVINYLDWIQKNMRL
>10_ENSOANG00000008025:ENSOANT00000012763 peptide:ENSOANP00000012761
pep:NOVEL_protein_coding
LLNWVLNSSGLNVSPIFLTVWAIKEVPGIEDYEVVYPEKLHVLHKRNIEEHEDPDKQEKYEPEVQYRIMLHGEGV
VLHLEKAKELLSPDYTETFYSSTGEEVTTGPQITEHCYYQGHILNEEISSASISTCAGLRGYFKLHDQRYLIEPL
KLTDQEEHAVFKYNHMKQDTINSTCAVNDSGQRKVEVQVKASRSGVSLGKDQYMQQKKYVEFFLVLDNTLYQRYG
PDQDELRKRVFDMVNFVSMVYKSINVLVELVGIETWTDADKIKVNPDASITLELFKNWRRSVLPRRKKHDIAQLL
TGVVFSEEMVGLAFVGTVCSPFHSVGVIQDHSQNRFIVAGTIAHEMGHNFGMTHDTDQCKCPTVTCVMDRALSNN
IPRGFSSCSQLSFKRFLSGKIPACIIRAPNPKDIISTPICGNLLLEVGEDCDCGTPKECTNNCCVAKTCKLKAGG
QCADGECCENCQIRKAGTLCRPVKDDCDLPEVCDGQSRKCPVDNYQVNGFPCQNGKGYCFMGKCPTLQKQCATIW
GEGVKAADAKCYEMNKVGLMYGHCKKINNSYVPCQPGHYLCGKLFCSGGSNSLTWEGSVMTFTTCKTFNTEKSDQ
NIDMVANGTKCGAEKVCSNGECVDLAKTYKSANCSSKCKGHAVCNHKLKCQCEEGWAPPDCKNTAVVMTMSIDAG
ENFSIIFLLLS
>14_ENSOANG00000008023:ENSOANT00000012761 peptide:ENSOANP00000012759
pep:NOVEL_protein_coding
EVVFPQNFPLAHTRTAKKSHFPGKQEKYQQVVQYQIAIKGDEVIFHLQKTRGLLSPDYTETHYSARGEEVTTSHQ
VTENCYYQGYILNERDSTVSLSICAGLRGFFMHHNQRYQIEPLKFTDQEEHAVFRYGDLEGGPANFTCGVKNSDW
PENPFTSRLSNRQAKQKNFLQEEKYLELFMVLDHAFFKEYDGNLEEIRRTVFETANMINMIYKTIEIYVSLVGLE
IWTDGDKIEVVPNASITLARFAFWRYDVLLKRKNHDQAHLLTGVGLRYPTVGLSYMNSMCNKNSVAIIEVCLVSS
NARSNIMVHEIGHTLGMGHTPQSCNCSTGNCVMVRHLSPIFPKDFSSCSQDKLREFLLWRRPHCLLSVPLFKTIV
TKPLCGNHILEVGEQCDCGTPEECTDLCCNAKSCRINMWVKCNGDCCYRCQIKKKGTLCRPAKHECDVPEVCDGR
STHCPKDQFRANGTPCHNGEGYCFGGECPTLQDQCTALWGEGSEVGDDDCYARNQDGKEGGYCKMVKNTFVPCAK
KDIKCGKIFCKGGSGVTPLHVEVEDYLTCKTFSPTLKAQVAQLPTTGTKCDDDRVCMNGECLELERVYHS
>15_ENSOANG00000008022:ENSOANT00000012758 peptide:ENSOANP00000012756
pep:NOVEL_protein_codi15_ng
MKLVDLVSANANISRQLFPLLFFFLVSAISELPGAKQYEVVFPQKLHTLHKRDVKEAEEEANNKEEIYESEVQYH
MKVQGEDIVLHLQKNKNLLSPDYTETLYSPTGERITTRPQHKEHCYYQGHIVNEKDSTASISTCGGLRGYFKHRD
QSYLIEPLKLTDQGEHAVYKYERKEPNRAKRSCGMKYSDSKNDLIRTPRAVKSREEQDFLQSEKFIELYLVLDNA
LYKHHKENQTVIRTLIFDVTNLLNVIYNTIKVHVALTGFEIWNDADKIKVVQNSGALFTNFLNWRRRDLLKRKTH
DHAQFLTGLSFNHQLTGSAASNSICLPTSVSIIEAFRKGKFSLVGVMSHELGHVLGMPDQPHTTKCPSGSCVMNQ
YLSSKFPKDFSPSCRSHFEKFLLSKKPRCLLNVPAPESIVTNPICGNGILERGEDCDCGAPEGCSNPCCEAKTCK
LKSQASC
>16_ENSOANG00000011661:ENSOANT00000018475 peptide:ENSOANP00000018472
pep:NOVEL_protein_coding
QGLQCMKMAGKSAIITICLVGYLFSAECTVFIDRERATKVLHRIRRYNSGKLEEFVQGNLERECIEEKCSFEEAR
EVFENTEKTTEFWKQYLDGNQCDSNPCLNGGQCKDDINAYECWCLPGFEGKNCELEESCKINNGRCMHFCTKSPD
NHLMCSCASGYRLGKDKKSCEPAVPFPCGRAAVALSNKKYTRGENIFFNMDLNSTEEEEVNQTNPVTDEVTQITP
VLKKLTRVVGGEDAMKGEIPWQVLLESKGEGFCGGAIINEKWVITAAHCIEPGVEITVIAGEHNVENDDKTEQRR
TVKQAILHPNYNAQISKYNNDIALLELENPLNLNRYVTPICIADKEYTNLFTKNGVGTVSGWGKVFNKGRTSPIL

QTLRVPFVDRATCLQSTKFTISNNMFCAGYRDGGKDSCQGDSGGPHTVEVGQTRFLTGIISWGEECAARGKYGIY
TKVSRFVKWIRDITKQT
>19_ENSOANG00000011917:ENSOANT00000018893 peptide:ENSOANP00000018890
pep:NOVEL_protein_coding
MRKHRHLPLVATFCLLLASPYIINAQHQEEDVKIGVAADIIFLVDSSWSIGKEHFQLVREFLYDVIKSLDVGDND
YRFALVQFSGNPHTEFLLNTYHANQDVLAHVATMPYLGGGSKTGKGLDYLIHHHLTKASGSRASDGIPQVIIVLT
DGQSQDDIVLPTAELKSADVTVFAIGVQDAEEGELKEIASEPLDMHVFNIETFTALHDLVGDLVSCVQTSMTPEM
AGAKEILKDITAQESADLIFLIDGSNNIGSVHFSAIRDFLVNFLERLAIGPQQIQVGVVQYSDEPSTVFSLNSYS
TKADVLDAVKALRFAGGEGANIGAALEFVVENHFTQATGSRVEEGVPQVLVLISAGQSSDEVDDGVVALKQASIF
SFGLGAQDADKAELQHIATDESFVFTTPEFRSLGDLQEQLLPYIVGVAQRNIVLQPPTIITQVVEVNKRDIVFLI
DGSSSLGVASFYAIREFLVRVIQKLEIGQDLIQVAVAQYADSVKPEFYFNTFQTKRDIVMALRRVKFLDGPARNT
GSALDYVRNYFFTSSAGHRAAEGVPKLLVLLTGGKSLDDVHQPAQELKRNGILAFAIGSRLAAKDELEDIAFDSS
LVFSPDEFKNVPLQGILLNFLAPLRTLSGTMQVHVNKRDIIFLLDGSLNVGNANFHYVRDFVMNLVNSLDVGRDN
IRIGLVQFSDTPETEFYLNTYQSKSDLLARLSQLQLKGGSTLNIGSALDFVLSNHFTEAGGSRINEHVPQLLLLL
TAGRSADSYLPASNALARAGVLTFSVGTSAADQAELEQIAFNPSQAYLMDDFSSLPALPQQLIQPLTEMISGGVE
EVPLAPTETKRDILFLFDGSANLVGQFPVVRDFLYKIIDQLNVKPDGTRVAVAQYSDNVKVESRFADHQQKAEIL
GVVRKMKIKTGKTLNVGAALDYARRNIFVKSAGSRIDEGVLQFLVLLVAGRSADSVAQPSDYLRRTGVVPFIFQA
RNADPTELQQIVLAPEFILAAESLPKIGELHPQIVNLLKSVQNGGQPQDPGEKKDVVFLIDGSDGVRTGFPLMKE
FLQRVVESLDVGPDRVRVGVVQYCERARPEFYLNTYNDQQSVVNGIRSLAHMGGSTLNTGAALNFVLRNIFTAPA
GSRITEGVPQFLILLTADRSQDDVRGPSVVLKRSGMVPFGIGIGKADIIEMKTISYVPDFALSIPAFRDLNSIQQ
VISERVSQLTREELEKLEPVVGDIPTRGRGSKRDIVFLIDGSQTARPEFTYIRSFIERLIENLDVGFDTTRVAVV
QFSEDPKVEFLLNAHSSKDEMQSAVRRISPKGGRQINTGTALEYVSKNIFDRALGSRIEEGVPQFLILFSSGNAD
DDVEEPAGHVKQVGVAPLTIGKNIDPEELVKISLSPEYVFSVSTFRELPSLEQKLLTPITTLTSEQIERILSSTQ
LPPPVVESDAADIVFLIDSSDSVKPDGLAHIRDFISSIVRKLNIGPSRVRIGVVQFSNDVFPEFYLKSHKTQSAV
LDALRRLRFKGGSPLYTSKALDFVAKNLFVKSAGSRIEDGVPQHLVLVLGGKSQDDVTRPSRVLGSAGIVRIGVG
SRNANRSELQAITNDQKYVFTVRDFRDLSLLESRIINSFGPSQATPPPHPPHPTPLPGRPNKKKADIVFLLDGSI
NFKRDHFQEVLNFVSGIVDTVYEDGDSIQVGLVQYNSDPTDEFFLKDFTNKEQIMDAISKVVYKGGRHANTKVAI
EHLTRHHFVPDAGSRLDQRVPQIAFIITGGKSIEDAQEASMALSGKRVKVFAVGVKDIDSTEVSGIASNSATAFR
VSTVQELSELSEQVLETLHDAMNEALCPGVPDVSRACNLDVILGFDGSAGQGVLESRVKNILDRITQMQKISCTS
NQEPTVRVSIMANSASGPVEAFDFSEYQPELFEKFQLLLGQRPYILTADTLKAYQDKFRKSPADSVKVVIHFTDG
TDENSARLEAASAALQQSGVNALIFVNLEHRDTNFDNVMELEFGRGFTYNRPLKLSLVDLDYELAEQLDSIAERA
CCGVPCKCSGQRGDRGVLGNIGQKGVPGEGGYRGYPGDEGGPGERGPPGLNGTQGFQGCPGHRGTKGSRGFPGDK
GELGEIGLDGIDGEDGDKGLPGSSGETGSAGRRGDKGAKGERGERGDVGIRGDPGDSGLDSRQRGPKGQKGEIGP
MGLPGNDGAPGSPGGNGKEGGFGRRGTVGVKGSKGGPGQPGIVGEPGLRGSQGSPGQLGPPGVIGEQGIPGPRGG
AGNPGVPGDRGRPGSLGRKGEPGEPGPKGGNGFRGPRGETGEDGRDGVGSEGRKGKKGEMGFPGYPGPKGAPGDR
GGEGGPGPKGNRGRRGNAGEPGPVGQKGDPGYPGSAGLKGNRGDSTDQCSLIQSIKDKCPCCYGPKECPVYPTEL
AFALDTSAGVNQDAFRRMREVLLRIIENLTIAESNCPRGARVAVVTYNNEVTTEVRFADSKRKSVLLQKIQNLQG
AFTSKQRSLETVMSFVARNTFKRARSGFLMRKVAVFFTNGPTKASPQLNEAILKLYDEGITPLFLTNREDRTLKN
ALQINNTAVGDAIILTSRDYSETLKKILTCHICLDICDPHESCGFGGQRLSFRDRRSAGTNVDLDVAFILDSSDT
TTPFQFNEMKKYIAYTVKQLELSPNPKSSHLLARVAVLQHAPYEYERNSSTLPVKVELSLTDYGSRDKLVDFLSN
KMTQLHGTRALGSAIDFTIETVFESVPNPRDLKVLILMLTGEVKKQELESLQRVIIDAKCKGYFFVILGIGRKVN
VKDIYSLSSEPNDVFFKLADKPTELNEEPLLRFSRLLPSFISSENAFYLSPDIRKQCDWFQGDQPAKNPVKFGQK
QLHVPNNVTSSITTKPLSTQPNPVSTTTNPVSTPSLTFRVTILTNQPLVLAQKWALELRWIDPIPRPTMRAHEGP
LNYPKYVEISAYVIHQQAKHSSADLSIKLPETSCTLPFQCCFRAFVSVILKACPQKTKAKGSREIQVFDITENSA
KLQWVNPEPSSPYIYDITITSAHDHSLVLKQNITSTERVIGGLRTGQKYQVVITGYLKSQARISYAGSFTTKKAQ
SSPPPPAPPKSASTSTVNLMVNTEPLAGAEAEICKLKKEEGTCRKFILKWYYDSETKSCARFWYGGCGGNENRFN
TQKECEKVCIPGHINPGVVATIGT
>new 24_2|294_aa:FA10V_TROCA-:402..450:E=7e-12
XCGKPQLLNRIVGGEDAKDGEWPWIVSIQKNRTHHCAGSLLTDRWIVTAAHCFKGSPDLSLLTVLLGAWTLTTPG
PQALRLSVAEVRPHPVYAWREGAPGDIALVRLASPVPFSEHILPICLPEASVPFPPETLCWIAGWGSIRDGVPLP
PPKKLQKLEVPIIAPETCSHLYRRGGGQQDTITPDMLCAGYREGKKDACLGDSGGPLMCQLEGSWLLAGIISWGE
GCAERDRPGVYIPLTAHQAWIRETVQEAQFLRPKLGGARATGPGAPKGLGGYRTRQGRAGALSPQTRKP
>27_ENSOANG00000002526:ENSOANT00000004001 peptide:ENSOANP00000004000
pep:NOVEL_protein_coding
CTGASAGERCQAPNPCLSSPCKNAGTCHAVLRGNIVDYTCSCRLGFSDRLCLTPEDNVCLNNPCRNGGSCDLISL
TEYKCRCPPGWSGKTCQQSDPCASNPCANGGHCLPFESSFVCKCLPGFHGQNCKMDINECNQNAAICRNGGSCIN
EIGSYHCLCKPAYTGPNCENLFVPCNPSPCQNGGTCRQTGDTTYECTCLPGFTGQNCEDNVDDCPGNNCKNGGTC
VDGVNTYNCRCPPEWTGQYCTEDVDECQLMPNACQNGGTCHNTHGGYNCVCVNGWTGEDCSENIDDCANAACFEG
ATCHDRVASFYCECPHGRTGLLCHLNDACISNPCNEGSNCDTNPVNGKAICTCPSGYMGPACNQDVNECSLGANP
CEHAGKCINTLGSFQCQCLQGYSGPRCEIDVNECLSNPCQNDATCLDQIGEFQCICMPGYEGVYCEINTDECASS
PCLHNGECIDKINEFHCQCPTGFNGHLCQFDMDECASTPCKNGAKCVDGPNAYTCECTEGFTGTHCEVDINECEP

DPCHYGTCKDGIATFSCLCQPGYTGHRCETNINECQSQPCKNGGTCYDRNNAYNCMCLKGTTGPNCEINLDDCAS
SPCDYGKCLDKINGYECACEPGYTGSMCNININECAANPCHNGGTCKDGINGFTCVCPEGYHDLTCLSEVNECNS
NPCIHGKCNDGLNGYKCDCDPGWSGTNCDINNNECESNPCMNGGTCKDMTSGYVCTCREGFSGPNCQMNINECAS
NPCLNQGTCIDDVAGYKCNCLLPYTGLTCKEVLAPCANSPCKNGGECKESEDFESFSCICPTGWQGQTCEIDINE
CVKSPCRNGAICQNNNGSYRCNCKAGYTGRNCETDIDDCQPNPCHNGGSCSDGINNAFCHCLAGFRGPQCEEDIN
ECASNPCKNGANCTDCVNSYTCTCPSGFSGIHCENNTPDCTESSCFNGGTCVDGINTFTCLCPPGFTGSYCQHDI
NECDSRPCLNGGTCQDSYGTYKCTCPQGYTGLNCQNLVRWCDSSPCKNGGKCWQTNNLYRCECNSGWTGLYCDVP
SVSCEVAAKQQGIDVTNLCRNSGLCMDTGNTHYCRCQAGYTGSYCEEQVDECLSNPCQNGATCTDYLGGYSCECV
TGYHGTNCSEEINECLSHPCQNGGTCIDLINTYKCSCPRGTQGVHCEINVDDCHPFTDPITRGPKCFNNGKCVDQ
VGGYSCNCPPGFVGERCEGDVNECLSNPCDPRGTQNCVQRVNDYKCECRQGYTGRRCDVVVDGCKGKPCKNGGTC
AVATNTGRGFICNCPPGFEGATCENDARTCGSLHCQNGGTCVSGHKSSKCLCLPPYTGPECQYPVSSPCLSNPCY
NQGTCEFTPEAPFYRCHCPAKFNGLYCHILDYSFVGGVGRDIIPPLIEENCEIPECKKDTGNKICNGKCNNHACG
WDGGDCSLNFNDPWKNCTQSLQCWKYFNDGNCDSQCNNAGCLFDGFDCQKVEVQCNPLYDQYCKDHFRDGHCDQG
CNNAECEWDSLDCAENMPAKLADGTLVVVVLTPPENLKNKSFKFLRAISRVLHTNVVFKIDDEGESMIYPYYGNE
EELKKHLIKRSTKGWGDGGKTSLYSEVTGRQRRELDQMDIRGSIVFLEIDNRQCIQSSSQCFQSATDVAAFLGAL
ASMGNLNIPYKIEAVQSETVESTKSSQLHLMYVVGAPLVILVLVMVGVVMSRKRRREHGQLWFPEGFKVTESSKK
KRREPLGEDSVGLKPLKNASEGALMDDNQNEWGDDDLETKKFRFEEQAMLPDVDDQADHRQWTQQHLDAADLRIS
SMAPTPPQGEIDADSMDVNVRGPDGFTPLMIASCSGGGLETGNSEEEEDAPAVISDFIYQGASLHNQTDRTGETA
LHLAARYSRSDAAKRLLEASADANIQDNMGRTPLHAAVSADAQGVFQILIRNRATDLDARMHDGTTPLILAARLA
VEGMLEDLINCHADVNAVDDLGKSALHWAAAVNNVDAAIVLLKNGANKDMQNNKEETPLFLAAREGSYETAKVLL
DHFANRDITDHMDRLPRDIAQERMHHDIVRLLDEYNLVRSPPLHNGPLGAPTLSPPLCSPNSYMGNLKPAAGKKT
RKPSTKGPGCAGKDPKDIKARRKKSQDGKGCLLDSSGVLSPVDSLESPRGYLSDVASPPLMASPFQQSPSVPLNH
MPGMPDTHLSLSHLNLAGKQEMAAALGGANRLAFDAAPPRLSHLPVSGPGAALGGGSVNFPVGGAAPLAGQCEWL
SRLQNGMVPNQYNPMRGNVQAGAHQPAAPALQHGLMAPLHGGLAAAATTTTLSQMMSYQGLPNTRLTAQPHLMQA
QQMQQMQQQQNLQQQLQQQNMQQPQQQQQHLSATPGASSHMGQNFLSNELSQSDVQQLGGATMAVHPILPQETQI
LPTPLPSSLAQPLTTTQFLTPPSQHSYSSPLDNTPNHQLQVPDHPFLTPSPESPDQWSSSSPHSNISDWSEGISS
PPTSMQSQIAHVPDAFK
>28_ENSOANG00000009837:ENSOANT00000015601 peptide:ENSOANP00000015598
pep:NOVEL_protein_coding
QVSLQYLSGSKWHHTCGGTLIDNNWVMTAGHCIGHTGEYSTMELGDMFLSIRNSDSLLLYIQQTTTFREDRVQVP
NDIALIKLAQPVSLSSKIQLACLPPANAILPHNFACYVTGWGRLQTGGALPDILQQGRLLVVDFETCSSSGWWGS
SVKTNMVCAGGDGVISSCNGDSGGPLNCLGDNGQWEVHGVVSFGSALGCNYYRKPSVFTRVSNYNSWINEVIASD
>29_1|126_aa:CRVP_TRIST-:13..51:E=2e-11
MEEEGMVDSVKGSREVKEDQNGVGAIGLGKKEKQKLLTIGFIVVSQLSPSRSTHKSMDVSFSSLSTSNPQVQKEI
VNKHNALRRQVSPPASNMLRMEWSPEAAKNAKAWADQCTLQHSSPEKRVTX
>32_ENSOANG00000015818:ENSOANT00000024943 peptide:ENSOANP00000024939
pep:NOVEL_protein_coding
TCSICLDLLEDPTSLECAHNFCSTCITDYCSTETQDAQCSAQPRCPECRSPFQRDQCVPDTRLRSLVQKFKSRQQ
KMLTFFQEAKEKEPKALKLVDCCPSEGPILNIDALNHCLRNPKVQDTPVCLITVLGEQRTGKSFLLNQMLLALKA
MESGQDSWRPQGGEDLQGFQWGGGSDTITKGLWIWNKPFLRIRSGKWVAVFLLDIEGSMALDECKESNVKLSALT
MLLSSYQILNVFRILKEADLEYLEMFLHVAETIGEICGMEAVQTFRPFPFPPVRCSAPLTAPLLLQHLDILVRDW
GYSEEFGWAPGKAYMNDIIQKQKRKHPKVWKMLQEARTQCYLLPFAGKNMATKKGGNTAGNTHMDAEFCSSLHAY
VIHVLESANNHAKRGQKGPLTGNQLATAIEVLSDLMKHEKFGFSSPVEKAIQQHNLDLKQNKFKEFEEFLGKQNE
ATKPLFSALRIRPNTMKACLAKERDSILASFSEAYIGKDQRWAVETLQEKIQAKEETFLKAYFKRYCGHVAAAGG
TAAGVLALVGAGVGAGVAAAVVAAEAVPLMSAGIAGAWARVVGG
>33_ENSOANG00000013614:ENSOANT00000021475 peptide:ENSOANP00000021472
pep:NOVEL_protein_coding
DNPQRKRDYLCLAPLWLALVTLAAAGVLIWYFLGFKEEGTSSRLYSGSVAVLDRQFFPDLANHESGAFRSEIAKA
QIMLKELISATRLSAYYNSSTVYSFGAKPLTCFFWFILQVPNSKVQKMSPDWVKEVLVDELKARANASDALPQDD
QYEMDPGTLTLLEASLRDIIVLNSTLGMLCCSVSFSLLGAGEGRGVMGELGLGEEDLYRLPQAQKLRESKRERQW
GQLEERRGNGGARSILGDCRGRRERLILLLYGCSRVEPALDVLSSGPVMSVVWKKGLYSYDDPFTLAAQAVPFQV
CAVNLTLEESLELQGPIRTPYYPSYYSPSTHCTWHLKVPSPDYGVALWFDSYALQRGKKSGLCTQGQWTIQNRRM
CGQRILNAYAERIPVVTAAGLTINFTSQISLTGPGLQAHYSLYNTSDPCPGAFLCPLNGLCVPGCDGIKDCGSGM
DERNCVCPAKFQCPEDSACIALPKVCDRHLDCVDGSDEQHCNHTVPCGAFTFKCADGSCVKKPNPQCDDLPDCPD
QSDELHCDCGLQAPTNRILGGFNSVEGEWPWQASLQAQGRHICGGSLIADRWVLSAAHCFQKDSLALPAVWTVYL
GKLQQNSSRASEVSFKVSRLLLHPYYEEDTHDYDVALLQLDHPVVRSPVVRPLCLPAPTHFFEPGLKCWVTGWGA
LREGGSFSNTLQKVDVQIVHQDLCDEAYRFSITPRMMCAGYRKGKKDSCQGDSGSPLVCKEPSGRWFLAGLVSWG
LGCGRPNYFGVYTRISRVLDWIKQEMS
>35_ENSOANG00000014841:ENSOANT00000023374 peptide: ENSOANP00000023370
pep:NOVEL_protein_coding

FSVIGPAEPILALEGGDAEMLCHLSTKESAEDMEVRWFQSHPSNIVHLYEYGEEKFGRQMEEYQGRTKLVRDAID
YGSVAVRIRNVRVSDEGQYRCSFFNGVYDEEATLELQVVGLGQEPVLSHSCPTDHSSPHLRFQGGESDHLLNMKW
QDIAGQRQDMGEREVHEHPTPCHTQPSTHTYTRSMGILCCNNNNPIEGAQDPQSFLLPSPLFPTALSLIVALGVI
LPVLGLLIAGGLYLIWKHPRDKAGKGSRPDREGRDRQMRTHPTENEHSSQSGMQKNLKNKLALYKLCWGKSQRMP
NFRSQKSAERSFKVVPIPDHMESVYQLNEGVKKQCGHVCPLEQGGVGRVIPGNQSGNSSGTKCWEVEVGEKRSWY
LGVCRENVKRKWWISASPENGFWTVEKIEDRSWARTASRPRLPLIPRPRRVVVYLDCEAGDVSFYSGTDGCHIYT
FPRAAFSGTLRPFFYLWSSDPVPLT
>37_1|50_aa:VSPL_BOTAS-:203..252:E=1e-23
GDSGGPLVCNGKLQGLVSWGTFPCGQPNDPGVYTQVCKFTAWIRKTMKEN
>38_1|419_aa:gi|68566091|sp|Q5R231.1|ACTP1_ACTVL-:131..177:E=1e-10
XYNRLRESGVHSLSPSRSPGPPAPSTWRDRRGEAHMEQTIEQLVHQVDSRRCVGIEVTNGMSLEFHSVRATPPGG
EWAMVSKAADKSGMIRMEERPGIWQGLISRPQLSSPDANPLEGDAANVAPGTAGLPVTGRPRAGVGLKVQMPESL
RLGQTLRILWPPQVPLATAPAPRDPEAGPETYCYSGHTHLPPSPIIPPKTKRHCIFVKTDGAPRGSVGLLVYKIR
LNLSLAILFSNPYDYNLYSIEFAVALYERDMAGTELDVLYDHIYKQKEQGSLKVAKRKLGSTQEPLVLKERGVRV
SATMSNDAKAVIRAVPLSSVYPRSLLLLSFPLPLQNTTTTNKMSVKHFLFAEHWAKHSPAASASPPIGWVNTASL
INRLIKRSLLLPCIIGTFTNRIVDIYVWYRCKPYPGHLQVSFDL
>42_ENSOANG00000005496:ENSOANT00000008735 peptide:ENSOANP00000008733
pep:NOVEL_protein_coding
VIDFESSDKSGDLLGDSYIHPKYVELAVAVDNARYRFRESNLSTVIQDTIQIVSYMDSYFEKMKAHVFLKALEVW
TDNDKVNTSHKKLSRILGEFSVYQDKVLYPRTQHDFAHLFVRRYYSDAVGWAFVASACMRKYMASTSTLAGPYLL
SSSLYSTHELGHGCGLGHDYRYCQCSAKRCIMFSRGSTPKGGFSNCSFNYFFNFVSKTATCLNNIPVVGFVEGRC
GNKVVEGEEQCDCGTESECKKDACCRPDCTLSPGAQCISGACCRRCQFVPAKMMCRRRQSECDLEEYCNGTSNLC
PEDVYKLDGTPCSDGAICYHGGCHSRLRQCRNLFGKEAKTAPLLCYKEVNEKVDRFGNCGLSGRGYKKCSVRDTL
CGRVQCVNVMTVPSMPDHTSVIQTHVKASNTTCWGTDYHVSMHTLRIADMGDVKDGTFCGTGLICIKRTCLNMSL
LSYNYEPQMCNHRGVFNNKRNCHCDRGWEPPFCETPGEGGSIDSGPVGTLPVAKTYKIWPVFIIRSTLFVALFIA
TSV
>43_1|46_aa:VSP10_TRIST-:151..199:E=6e-12
XVYPDVLQCASVHLVSQEACKRAYPGQITDNMICAGEHDGGKDSCQ
>44_ENSOANG00000010997:ENSOANT00000017426 peptide:ENSOANP00000017423
pep:NOVEL_protein_coding
VSDKCSGLKVVGRCRASMPRWWYNSTAQICQPFIYGGCGGNDNNFLTQDGCLQACTGPSGLGTGYTWHRASRERL
GANSRVSGPGAVQRNGELDKDPFNYDDHCAASAMTGPCRAAFPRWYFDAQKDACVSFIYGGCRGNRNNYLTQRDC
MASCHEGNERRMLVPGSKAAVLAVVLAGMAAALLGVMVVVFVRMARGAQSGAFDTIWTPVDDKEYLVKNAYTL
>46_ENSOANG00000012854:ENSOANT00000020334 peptide:ENSOANP00000020331
pep:NOVEL_protein_coding
HARIVNGEEAKPGSWPWQASLQDASGWHFCGGSLINSQWVVTAAHCEVTKNDFVILGEHDRSSGEEVIQKMAVEK
VFTHPDWDNYYIKNDISLIKLASPVNFSQTVSPVCLAEAGEDYESGALVVTSGWGKTRYNALVTPNQLQQTSLPL
LSAPECKTFWGSKIDENVMVCAGAAGSSSCMGDSGGPLVQKRDGAWYLVGIVSWGSSYCSTSTPGVYGRVTAFRD
WVDQIIADN
>47_ENSOANG00000011269:ENSOANT00000017858 peptide:ENSOANP00000017855
pep:NOVEL_protein_coding
RSYESIVKHNQPCGGFKIKQLEEEAETVALEIRRLQAEMEEDDVSFLMKHKSRKRRLTCTAEPEPVQSGLLIDLS
QALDSLQFRVWKKMLNIVEAVPFSFDPNTAGGWLSVSDSLTAVTNQGYRVPVENPERFSSAPCLLGSRVLAHGAH
TWEVEVGTLPSWRVGVTRALPGARPLQSTAHDARAGFWFVARARGLEGDQLVASEPAAASPLCLQLPRRLRVELE
CEDGELSFYDAERRRHLYTFHASFGPVRPYFYLGGT
>48_1|180_aa:VESP_LACMU-:51..148:E=5e-21
XNVTLDPHTAHPQLVLYEDRKRVTLRDTRRYLPYKPERFNDSACVVGRESFTSGTHCWEVEVGEATRWSLGVCRE
NVRRKGGTSESPADGFWAVKKDYDGYYALTSPRVPLPLTTSPSQVVVYLDFEAGDVSFYSGTDGSHIYTFPHAAF
SGTLRPFFRLYRVYDFLTICPVAAPSQETP
>49_1|49_aa:CRVP2_LAPHA-:92..138:E=2e-22
XTNCGENLFMSSSPNSWSNAIQSWYDEVKDFTYGKGAKTANAVIGHYTQ
>50_1|185_aa:VESP_LACMU-:58..153:E=2e-25
XLGSNPHIHLEGYKDGGIQLGCSSAGWYPEPQVQWRDAKGKMVPSLSKSQPHTLNDLFGVASSVIVQEDSVGTIT
CSIRNPLLNQEKGAVISIAGKIQAKLSWRLAQLHAVDVTLDPDTAHPELILSEDLKRVTRGDTRQTLPDNPERFD
VFPCVLGHEGFTSGRHYWEVEGCSCLCPHFLCCSP
>51_ENSOANG00000012443:ENSOANT00000019691 peptide:ENSOANP00000019688
pep:NOVEL_protein_coding
MVGFPASSMPSCLVHLLLFLLLPKDMLGSAQFSVIGPDKPILALVGGDAELSCHLNPKRSAEHMEIKWLQSQSLN
IIHLYDNGQDNNNVQMDKYQGRTELVRNDIKEGRLALRISEVRATDDGQYLCIFNEGPLSEETTLELQVAEMGSD
PHIRLVGHEDGRIRLECSSAGWYPEPQVLWRDAREETFPSLSKALSQTADGLFGVAASVIIQEGSMGTVSCSVQN
SCLSREKISAFISISAPLFPMALSLMVALGVTIPLLGLLIAGGLYLIWKQNRDKALQFRPKPTTPYPHPAIQFLE

CNHNPSRAKLLIELIWILGSLHHSSDASFLCSLAGWSQAQLHKGNIILDPKNGLSLYLILYGEFETRWKWEMRKG
NCQNTLRRFYRCACVLGQENSRQGDCWEVKVGDKMGWHGICRENVKRKGWIFVLPEEGFWTVEKNEDGYWARTSP
WTRLSLTKPPHRVAVYLDYEAGVISFYSGTDGSHIYTFPRVTFSGTLRPFFWLSSTDPIPLTIFSV
>56_ENSOANG00000004530:ENSOANT00000007184 peptide:ENSOANP00000007182
pep:NOVEL_protein_coding
RAFEEEGSQGSGFLLAALGRLNGRQRAHARHDDWGFSSSEVVIPRRLTPRGGEAEEPGRLSYLLPLEGRRHVLHL
RPKKLLLPRRLPVFTFTARGELLEEQPYVPHGCYYRGAVEGAPGSLATFSTCFRGLWGMLRLHGRLYQVEPLPAS
ATFEHRVSRLDEKAPGNLTCGLTFREIVRQEPQVWLPPSYGKHWNTSYVHPKYAELAVAVDNAMYQFRQSNLSRV
IEDILTLYNFADSHFQELKAHLFLKALEVWTDRDKVNTRVSTILSVLREFSSYLERDMYPRIRPDLAHLFVFKRY
SDGRAWSYHDSACNPSKMGSISSLIDFSLPNCGKWFTHELGHGFGMQHDENYCKCNASTCLMGPHDYNHGGFSNC
SFNEYFTFTSRSASCLNDVPEHLFIVENCGNKVVEKGEECDCGSEEECEKDACCLSNCTLSPNAECAYGLCCKGC
QIVPATTVCRPRQNECDLDEFCNGTTALCPENMYKQDGTPCSDSAVCYRGMCRSHLRQCKALFGKEALDGPLLCY
TEVNEYIDRFGNCGLEGNGYRECLVRDALCGRVQCINVKTVPSMPEHTSVIQTHLKANDTTCWGTDYHAPMQTLK
IADVGNVEDGTTCGPGLVCINRTCVSLSLLNFDCLSEKCNNRGACNNKRNCHCTFGWAPPFCDTPGDGGSVDSGP
AGNWVDEATTFHIWPVFPIRIVLFVASLILAAVKSMKFREKAPLEETRHLSAPPAD
>57_2|488_aa:gi|82189787|sp|O42138.1|VMED_AGKCL-:574..609:E=2.82e-27
LKARIFLKALEVWTDRDKVNTKHPELLPILDEFLPYQENNIYPRVQQDLAHLFLLKTFPDALGWAYLGTACNAKW
MGSISSLPGKLLFEPSLWFSHELGHICGIAHDGEYCTCQRLKCIMGASGRLHGAFSNCSFADFFTSTSQFASCLN
NIPEQFLVVEKCGNKVVEWGEECDCGSERECEKDACCLSSCKLSPGSVCVSGLCCKRCQFVLAKKVCRPRQSECD
LDEFCNGTSNMCPEDVYKQDGTPCSDGAVCYHGSCHSHLGQCRALFGKEAMVAPLLCYEEVNGYVDRFGNCGLEG
NRYRKCAVRDMLCGRVQCVNVKIIPSMPDHTSVIQTHVKASRTMCWGTDYHAAMHTMRIVDIGDVKDGSFCGTGL
ICINRTCLNVSLLNYDCEPQKCNHRGVCNNKRNCHCDYGWAPPFCETPGDGGSIDSGPAGKPVAAKTFQVWPVFI
LRIILFVAALILSSTQLMKFEKKASLKEMEKKSKKRLK
>59_ENSOANG00000007480:ENSOANT00000011909 peptide:ENSOANP00000011907
pep:NOVEL_protein_coding
VSPAHQSPRPCSFLTVSLSVTAQFSVIGPAEPVLALEGEDTEMHCHLNPKKSVEFMEARWFRSQPSNVVHLYDSG
AELFGEQMEEYQGRTELVRDAMDYGSVAVRLRNVRVSDEGNYLCSFYDGNSDDEAPLELQVVVSNVGVSPDLGTV
KSRDRTLFWWSQPSLHPSPTALVSIPTTNSADSPVMYFIFCLPLYHAVCAPQTTRFSALHSTLLCTQEGRPGNSV
SAPLFPSGLSLMVALGVTLPVLGLLIAGGLYVFWNQHRDKEGSLTSFCSFSGKLQMELKWSLFQLHEASVTLYPD
TAHPGLVLSEDRKRVTVGDKRQDLPDNPERFSHNSCVLGRERFTSGRHCWVVEVGERRVWYLGVCRENVRRKAGI
SVSPALGFWTLEKYGDGYWAHTSPRIPLPLTTRSRRVVVYLDCEAGAVSFYSGTDGSHIYTFSHAAFSGTLRPFF
WLWSSDPAPLTICPV
>60_2|398_aa:VESP_OPHHA-:146..157:E=2.44e-08
MKQSGLGSRTPIPTAFHPSASPLIIPPIRPAVALAPNRGDSASVTEPGIPLPAVDVTLDPSSADLYLIVSEDKKA
VRSVGKKLDLADLEDRFDNPFAVLGHQRFDRGCHYWEVGVEGKTRWTLGLCKQSIPRKGRYISASPESGFWALSP
KNTDNYQALTSSRAPIYVIEPPRAVGIFLEYEEGRVSFCNVTEGTWLYTFKASFTETLRPYFYPGPLSKGKNVNP
LIILPRPREGRGARSDGPSTQESKPLMDGDGGETGARRSDATHPRSHGSPPSDPGSAPRSSDSRPTIASPSWAWD
RGEQPDNLGSTAKLGTVPPAQSERLTDTRALDEAPDCSTDRSIRHPAEKPGASPAVIQPTRFAPLMPTYSPHLRF
IRLAANPSAASYLRPSGRHPLHI
>61_2|140_aa:VSPP_CERCE-:199..241:E=2e-08
XSLICKMGIKLVNPICNMDCVQPINLHLSQPLVQCLAHMPLPPPYKLQEVQITIFKLEECQPIFHIVSHEIQKDM
FCAGDKKKWQDSCQGDSGGPLACKINGAWTLIGVVSWGIRCATPNFPGVYTNVSYYRDWIDRRMS
>62_ENSOANG00000007093:ENSOANT00000011287 peptide:ENSOANP00000011285
pep:NOVEL_protein_coding
MVILLLLAAMLPGAATVISCYGDTGKPVDWFAVYKLPELPDSRARSGMSYMYLDGKTEGWREGTGLINSTQGAVG
RTLQPLYRGNASQCDGLAFLLYNDQPPKGSGVKDKASRGHTKGVLLLDKDGGFWMIHSTPHFPPPASTTYSWPDT
ARPKGQSFLCVSFSYSQFQEIGKQLAYTFPLVYNYSVEGTFAQDLPELLAASQGHHVKSAPTAAWHSLPLAGRSS
SALPNLVPLQMVRPGWLASALGSDLDVQFWPNSPGVLPSNCSGPFRVLNVVHTAFPPPAGPTFSATLDHSKWCVA
ARGPWTCVGDMNRNQGEEHRGGGILCSRLPALWKAFQPLVQKCQPC
>63_1|263_aa:BLTX_BLABR-:187..230:E=2e-15
AHISSCLQDVSTWMSARHLKLNMPKTELLIFPPKPCPLPDFPVTVDGTTILPISQARNLGDIVDSVFSFTPHIQS
VTNICRSHLHNIAKIHPFLSIQTAIVLSILHSTARIIFLQKCFKNVAPLLKNLQWLPVDLRMKQKLLTLGFKAVH
PVAPSYLSSLLSFYCLPPIFPSELQCVELQILPNEICDLAHPEKVTEFMLCAGLMQGGKDSCQGDSGGPLICNNT
LQGITSWGHFPCGLPGKPGLSTKVFAYLDWIAKTMEDN
>64_ENSOANG00000013077:ENSOANT00000020676 peptide:ENSOANP00000020673
pep:NOVEL_protein_coding
SRHRLVLLIPAQMITFLLFLPHLLQNSLVSGAEGSTSARDLVGIVGGQDAQKNKWPWQVSLRHRGQHLCGGTLIH
RRWVLTAAHCFDCFDKISAYDVQTGAWKLDQADPAKLIPVKNIAIHNNYLKNDLVGGDIALVELGRAVDKSGWNK
DIQLPEAMAQLTKGMPCWVTGWGYIGEKDKLPPPRTLQEAQVPIFNTSVCKKNYFKINRLILDDMLCAGYKKGKK
DSCQGDSGGPLACETTSKGNWTLVGVVSWGNGCGRSRFPGVYVNVSYYKTWIQEKIRQ
>66_1|427_aa:VESP_OPHHA-:130..157:E=6e-24

MIAIASEVQLTCEAALEGRSATRIPVEEAQQQAEALLDCRIATDEEWAQFGGTLFIAPTAALVDWKMRGECRTFD
HPRQQPSVGGLGASQEPHVKMHKLMGRTVSTHFANVTGHTDNQEEISEETALSGATCESPSFQLEPGEIKSDGEI
EEEEEEEEEERGHLRFSIEERLLQRAWPSSELPVFLRKGRAWNTETSKRRRWLPSANPVEAVIAVLVGSEEIYLA
KPTSLSREDRGHPDPGQSFRSKDGTQENRKGLTPVTLDPRTAHPCLILSEDLKRTSVGDAHQKLPDNPERFTSWF
CVLGREGFATGRHYWEVEVGEQGGCAVGVARESVKRKGGVRFDPGEGVWAVEFCQGQAWALTSPMTPLDLPRGPR
WVGVALDYERGQVSFSDAETQVPLFTFNAVFTERVRPLFWLCGRGSRLALRP
>71_ENSOANG00000001418:ENSOANT00000002247 peptide:ENSOANP00000002246
pep:NOVEL_protein_coding
MKSLLMFLLSLLFPPGAGAGEIIGGREVKPHSRPYMAYLKYYKNGKVHECGGFLVRKDFVLTAAHCRGSKMGVLL
GAHSIAYREATQQRIPVAEKFSHDYNNRTHVNDIMLLKLAHAANMTKEVNVIRLPLPVTNVKPGTTCSVAGWGMM
GANGPKTSTLQEVQLEVMPPAQCSFYRSFQPSCQLCVGNPKSYKSSYKGDSGGPMVCGKQAQGIVSYGRWAGKPP
NVYTRISFYLPWIKEILQK
>72_ENSOANG00000013866:ENSOANT00000021872 peptide:ENSOANP00000021869
pep:NOVEL_protein_coding
LNASRYQVQVGELKLYSLPPSKLIPVKRIIVHERYNGYPQRGADIALLELSRSAVLSKQVKAIQLPSPRLPVQLL
TKCTVTGWGNIKQGVPLPPPYTLQELTVTIFNSEVCRRNYQEITYKIQDDMFCAGDKKEKKGICYGDFGGPLACE
GKGSWTLVGMVSWGMPCVLPHFSGVYTNVSIYTEWIRQKPPLLPWA
>73_ENSOANG00000013005:ENSOANT00000020565 peptide:ENSOANP00000020562
pep:NOVEL_protein_coding
EGTSGKTALHLAVESQEAGLVRFLLGHGARVDARMFNGCTPLHLAVGRRQAGIASSLCQAGADTLLRNMEDETPQ
DLADGHGDVRVFDDLKISGKPLICAD
>80_ENSOANG00000000478:ENSOANT00000000762 peptide:ENSOANP00000000761
pep:NOVEL_protein_coding
LAAALSENSAEGSGRWRNARRCAANVTMDPDTAHPDLVLSGDQRSVRLRDTWQAQPDNPKRFDYVVSVLGSPSFT
AGRHYWEVEVEGKTKWTLGVCKESVRRKGVITVTPEEGFWVLRLTQGKKYSALTSRRTPLSLRVPPRRVGILLDY
EAGAIAFYNVTDPSRIFTFTSSFSGPLRPYFCPRASDGGRNLAPLTICPI
>83_ENSOANG00000011236:ENSOANT00000017802 peptide:ENSOANP00000017799
pep:NOVEL_protein_coding
MALFILALTLVAAAAATDKEKIIAGEKCKEDGHPYQVAVYRGGGLLCGGVLIHPGWVLTAAHCRKPQLQVLLGKY
NLHKQEEFQQLSPVAQPFPHPDYDRGRHDNDIMLLRLAHPTPVSRHIKPLSLETDCNANSSSCLISGWGKTDREG
QYPGRGGKCKPSQNRSGLGLPLPLETEMGRGSGSGKETLYNRISRNVPCQRSEQMGRDPCLGDSGGPLVCGGRLR
GLVSWGEVPCGTGEKPGVYTNVCRYVDWIRETIRGN
>84_ENSOANG00000015023:ENSOANT00000023650 peptide: ENSOANP00000023646
pep:NOVEL_protein_coding
KIIEGVECQPDSHPWQAALFRGNELHCGGVLVNRNWVLTAAHCKLPEYQVYLGQHNLKAKEKGGQVIRARTSYRH
PNYSTETHVNDLMLIRLDRAASLTGRIRPLPLPTSCDKPGTKCTVSGWGTTTSPEDGPGPDVPDPKDPDL
>88_ENSOANG00000011994:ENSOANT00000019002 peptide: ENSOANP00000018999
pep:NOVEL_protein_coding
MLVVDKWLSGLSNRLPSPSREPSSYSVILGTNTLEPISSDGVTRNVKQIVAHPSFTGSVKASYDIALLELSEPVS
FTEKIQPICIADASSRPASGTPCWTTGWGSIAGEENLPPPVTLQKVEVPLIYREACDNFYHQSQPSSPEEPGMIC
AGYPEGQRDSCHGDSGGPLACPVDGTWVLTGVVSFGEGCALPNRPGVYADVATYTSWILENIPQGNGK
>89_ENSOANG00000007297:ENSOANT00000011622 peptide:ENSOANP00000011620
pep:NOVEL_protein_coding
QLRPLLLLLLLLVLLPPTPGVRAAPPDNNTAICLLPKDEGPCRAILPSYYYDRYTQTCLLFSYGGCKGNANNFET
LKACQAACGKLAKVPKICRMDTRSDTCKGKVEKYFFNMSSMACEKFSFGECPVGPNQFSDKASCVRFCSPKSDVP
KFCSSPLDRGSCSAHVTRYYFNVESGTCEEFSYSGCGGNHNNFVSQKDCTKTCSRGMERPPPRLRPSAVRSWRR
>92_ENSOANG00000005244:ENSOANT00000008336 peptide:ENSOANP00000008334
pep:NOVEL_protein_coding
LYPLKISHSSCALKADGGPCRAMLRRYFFNISSRKCEEFEYGGCGGNENKFLMLEDCQKKCISAIDNSSKRMKIF
RGSDVQREKPEFCYLEEDVGICRGLISRYFYNNQSKQCEEFKYGGCLGNENNFESLEECKRTCGDGLDSLQVDKE
EIQPDIIVKPIVPISVSIPLLLEFSSPSWCRTPAERGLCRANEKRFFYNAVIGRCRPFNYSGCGGNENNFTTRKA
CLQNCKKGFNRKVVKGGLIKTKRKRKKLPVKITYEKILIGKI
>93_1|421_aa:VESP_LACMU-:60..138:E=2e-13
XRFSLDPETANEYLCLSQGNKSVEWVIPPQVVSQHRKRFVGEAYVLGSTGFNSGQHYWEVEVEGKGLCKIGVATE
SVERKSSRGHEETIWSLTICTPFHVLIGDEPDPNIEVPRVVGVLLDYEEGQVPGRSRPCGLNKVEFLRWSRTEAE
GKGRDEAVPAGLLGGRTDLGGGRHPPGKGSNTGGVKGTDEVTEAQRSEVTCPRSHSRHLGESELEPTSSDFQAVL
YPLGHAARCTLPSTQYHALHRRDWNSSEDFPICCDGSVDGPESRKEGGEAGRNSSTEDIYVIFGTFVDPAEEETL
ERRSRCEGCCGERVTLGPESWRSQEEAGDVPGTGTTHMEKTGEMKVINLHDNINLTFASVQGPQNSWTRLGHLIR
RIQVHYPNGSYQMYETYKLQMPLQTCLPCPTVREYNLTETKVFIVD
>94_ENSOANG00000012140:ENSOANT00000019231 peptide:ENSOANP00000019228
pep:NOVEL_protein_coding

VLMTVLVFFTSGKGYKTSPLPDIENEEFIKDCVQMHNKLRSEVNPSSSNMKYMTWDPDLAKTARAWAKTCQFKHN
IYLKQPKMVHPSFSSVGENIWTGSLSLFSASSAIQKWYDEVQYYTYETRSCTKVCGHYTQVVWATSYKVGCAVHL
CPQVAGFKGLTNGAHFICNYGLAGNYPTWPYKTGKPCSSCDNNENCQDKLCRHPGGNRPPVWPSPTISSPPLNSS
HTACGQGCAFILILRSVFLLLTVGATLLIKWRYPRIFYYK
>96_ENSOANG00000010013:ENSOANT00000015877 peptide:ENSOANP00000015874
pep:NOVEL_protein_coding
MMGFPDSSMPRCLLFLQLLQMATLGSAQFAVIGPAEPILALEGGDAELSCHLNPKMSAEMMEVRWYRAKFFPAVY
VYRAGQDTDKEQMEEYRGRTTLVRDAIANGSVALRIHNIRASDEGRYHCFFQRDAVSEETILELRVAGEIPMTTL
GQFLSVHLPGNPGGTGLGLQCLPGPQRTWDLELRGNCKLVAGREYEWKDQPGEQMEEYRGRTEMMRNAIAEGSVT
VKIHNQRICKQNKSVVEVGSSISFVTAQFSVIGPVKPILVLVGEDAKLPCHLEPKTSAKDMEVRWFRTHASNVVH
LCQNGTDHLGEQLEGYRGRTELLRDAIANGSLAVRIHKVRASDDGQYCCSFQHGSVYKWTTLELQVTTLGSDPHI
RMEWYKGGGIWMECTSAGWYPEPVVRWRDARGQTVPALSESRPRARDGLYGVAVSLVVRDDSVGMVSCSIWNPRL
GQEKKSSISITGR
>98_1|72_aa:HELO_HELHO-:200..242:E=3e-12
XGNNREKINEPYQRGTPCGSCKNACDKGLCTNPCKYENKWSNCEELKNSATCNHPQVKSDCEASCKCTTEIK
>99_ENSOANG00000009231:ENSOANT00000014702 peptide:ENSOANP00000014699
pep:NOVEL_protein_coding
VSYVIQKEGRKHVIHLERNKELLPKDFVVYTYNKEGTLLSDYPVVQDHCHYRGYVEGVPNSAVALSTCSGLRGLL
HFENTSYGIEPLESSSGLEHLLYPLSNNKQSTTQLCGNHTHVFNKDGNLGVFLADMKKRRAILPQTRYVELFLVV
DRERMYIMLNIRIVLVGLEIWTHSNPISIVGSAGDVLGNFVQWREKNLITRRRHDSAQLVLRKEFRGTAGMAFVG
TVCSRSHAGGINVFMHSRVEMFASIVAHELGHNLGMNHDDERTCSCAASSCIMNSGATCSRGSRNFSSCSADDFE
KLTLNKGGSCLLNIPKPDESYSAPFCGNKLVDPGEECDCGSPKECELDPCCEKNCKLQSGADCAYGDCCHKCRFL
PGGTVCRASANECDLPEYCNGSSQFCQQDVSIQNGYQCQNNKAYCYNGMCQSYDAQCQAIFG
>100_2|498_aa:KC3_ANESU-:5..55:E=6e-20
MRNRDLPTPCSPHSAVLMSTSPLSAWHRICSSQCDMKSSNLLLLAMVLSFRVLVSWGAVEQAKESECATNAVLCN
DPCRGDESCPSGQKCCNTGCERTCSSPKPTEDDPLEPAKVPKLPVLVTGPSILGAPGDGAGKPGVCPLDVRSCPQ
PGQPLCLSDSHCPFNQKCCYSACHLRCMVPILGRPGRCPPQAAAIRCVRPEPDQCRSDDDCLDLKRCCYRRCGLK
CVEPEGLPVLPRIWPQMEPAVPPPQPPGPFPLSGARPPPLTQVLSGGLTPPTPPFCTLPFVIGNCNMWKPRFFYN
VATKRCERFMYSGCQGNQNNFLTKAKCLRSCQRLEHLGKYNGCLKSSSSPQEIASLMDGAGAQGKGPENGRSRRI
FSVEIRKRFTLTAFGAVNPALRPRLSPVPPPPPSTGEFGLVADGLPLGWPLPNLGMDWGPCQAKIPRFFYNAVTR
RCTKFFYGGCKGNPNNFVKLDSCKAVCEAQDQEGGPGDRADRKDTEEV
>101_1|328_aa:VESP_LACMU-:51..141:E=9e-13
XQFSVIGPAEPTLALEGQDAEMPCHLNTKESAEDMEGRLFRSQPSNIVHLYQHGKDQFGRQMEAYRGRTELVRDA
MDYGSVAVRLHNVRVSDEGNYHCSFFDGSQNEAPLELQVVAVSLMVALGVTLPMVGLLTAGGFYLFWKQHRDKEK
FLLELRQTLVELRTTNVTLDPDMANRYLVLSEDRKQVTRGDTWQDLPNSPDRFSHDFCVLGRERFTSGRCCWEVE
VGERKGCSLICKMGMKTVSPTWDNLIPLCLPQRLEQCSAHSQASESCKGLGTTMFDEKHYGTYFILTQAHNLGVI
VDFALSFTPHIQTVTKTCWSHLHKEQLN
>102_ENSOANG00000000592:ENSOANT00000000934 peptide:ENSOANP00000000933
pep:NOVEL_protein_coding
MAGCPESFTSRFLVILLFLQLLTMGSAQFSVIGPAEPVLALEGGDTEMPCHLNPKESAEFMEVRWFRSRPSNIVH
LYDNREELFREQMEEYQGRTELVRDAMDYGSVAVRLLNVRVSDEGNYHCSFYDGNTVDRAPLELQVTHTTHNLTF
PPEEDPTNPVSVSTLIHGGGETIRQEGRPGVFCPCFGLSACPEAKTQNFISAPLFPTALSLMVALGVTLPVLGLI
IAGGLYLIWKQNMDIGKGEKGSRPDSQEKLLVKLRWSRAQLHQTNVTLDPHTAHPQLVLSEDRKRVSLGDTRHGL
PANPERFSDNWCVLGRERFTSGRHCWEVEVGEKRSWYLGVCRENMRRKVGISESPAGGLWAVKRDNGEYWAITSP
RVPLPLTTPPSRVVVYLDYEAGDVSFYSGTDGSHIYTFPRAAFSGALRPFFYLWSYDSVPLIICPVPDGAGGDPV
PAHSRDLN
>103_1|213_aa:VESP_LACMU-:51..148:E=7e-19
XKLQIELRWSRGQLHMANVTLDPDTAYRFLVLSEDGKRVTVGFIRQDLPDKPERFSDVWCVLGRECFTSGTHCWA
VDVEDATVWSLGVCRENVRRKGKISESPADGLWAVKKEREQYWALTSPRVPLSLTTSPRRVVVYLDCEAGDVSFY
SGTDGSHIYTFPRAAFSGTLRPFFRLYYLYQSLKICPVPGHTASQCDASYPPTLSLRSMTQQE
>105_ENSOANG00000021318:ENSOANT00000030925 peptide:ENSOANP00000027125
pep:NOVEL_protein_coding
LINLIIIMIISGKGSVILVEIIGGKEAIPHSHPYIASLQVNGRHVCGATLVHPQWVLTAAHCLNQGIRDVRVVLG
LHKRRDSGRDFTVKKLVYHPEY
>106_3|507_aa:IVBTI_OXYSC-:4..60:E=7e-19
MQSSKDPTSHPTTTSCILDVSVQESCLERSRDCLRACCALPGCNLALVELPPEAGGPGGLDEPVTACYLLDCLYE
QTFVCKFTRKPGFLNYLTRDVYQAYRDLQKHGFGRGSRIPRTWAGTDLKVQAQEPLVLRGTGDTDWHLLQGDASV
RVEKKEADQLEVWGLKEGSYVFQLTATNPDGQRDTDNVTITVLSSEQTADYCLVPKKVGRCRGSFPRWFYNSTEQ
QCQKFVYGGCLGNKNNYLREEECKMACKDVRGPLVESRQNPDPTRHTCSEVCQASQFRCRNGCCIDAFLECDETS
DCADNSDEAGCDQYISGFDELKQVDFPSEKDISGFDELKQVDSPSEKVPFFSPGTIGGLHKTQQNTFVDVPDTGV

CEDSLLRWYYNPFTERCFRFTYGGCPGNNNNFEGEQECLQACEGVSKKDVFGLRRETLTAGSVSVEVAVAVFLAT
SIVVVLALLGYCFFKKRRKGSSRRRRHPPPPPTGASSTVSTAEDTEHLVYNHTTKPL
>107_1|33_aa:HELO_HELHO-:139..171:E=6e-18
VVWYNSYQVGCSAAHCPNQPVFKYYLVCQYCPX
>108_ENSOANG00000003027:ENSOANT00000004798 peptide:ENSOANP00000004797
pep:NOVEL_protein_coding
LRFGNGLTAGDTWQDLPNNPERFNYNWCVLGRQSFTSGRHCWAVEVEVKNDWSLGVCRENVRRKGGISESPADGF
WAVQKLYGDYRALTSPRVYLSLTTFPRRVVVYLDYEAGDVSFYNGTDGSHIYTFPRAAFSGTLRPFFRLYYTDEF
LTICPV
>new _111 lcl|GenomeScan_predicted_peptide_4|832_aa:BLTX_BLABR-
:28..82:E=2e-08
MESPAGKAVEAAAHARGVGLHATDGIIILAVENEGRRRDVSLNSSALRNADYTLRLARLRFLFKDARDGYRRLTP
PPPFWYLLSAYYVQNTVLSAGAAEPSGGSPRRASVCLMLIETLSSCDVHTHPCRGTISVDLDCEPRVGQALSDLT
VYPVLNIMLGTLLYLRTFFSEIGHYEVSSILVCLFCQAAASFPDGQIVTPLRIFIFPLPPHPQTHNLRSRSLRGT
PNISHYLDATDKTKMALNSGIPPYGPYYENRGYQPENFSPPRHPGDPTAYVPYPAPYYPSPVPQYIPRVLTHSSS
PSVLVQPKPPSGIVVTSKAKKVICIAFSVAILLIGAVIAAIFIWKFFENKCSGSGIECGSSGTCISPSFWCDGIS
HCPSGEDENRCVRLYGPKFVLQVYSSQNKSWYPVCQDDWNDDYGKIACKDMGYNRNSFHLSHGVTDSSGATSFMK
LNMSSRNIDLYKKLYNSDVCSSKMVVSLRCIECGTASRASQLNRIVGGAVASPGEWPWQVSLHVQGVHVCGGSII
TSEWIVTAAHCVEEPVSSPRYWTVFAGILRQSAMFYGSGYKVQKIISHPNYDTKSKNNDIALMKLQAPLTFNGKT
SDVLNAVTVPIIEPWKCNSRYVYNHLITPAMICAGYLKGGIDSCQGDSGGPLVTEKDALWWLVGDTSWGSGCAKA
NRPGVYGNLTLFTDWIYRQMQRSLPAAVDSEAVAVHAAPDLSAAGRLRVEIPGKQNWNHNSRQAAKGIRSDSFSY
SRAVEFACCGHEIYICGRGHVCVDPDAPDVDRSETPAYRKVSFPLRLGSCLGIRERSAVHARLSGKNAMRREVRV
CAVEAPA
>112_ENSOANG00000008031:ENSOANT00000012771 peptide:ENSOANP00000012769
pep:NOVEL_protein_coding
MAGSVEGGVLVWLVEEDVRCPICYDPLRSPATLPCGHSFCSACLCLYWERCAHLGLKGPEAPCSLCRWASSSRKL
PGPSVILQALVDKYLAALQESGLGSPGPHPDKDPQVNSLKGAVFRREVEDLVEQLESEIALLRSLEPHQKCELAD
DRNYLDKDSSCGMESVKAVPEPGSPNPFEKNISGIERTLDKLKKKLQETLIWAEAPEETLQAVASPSILLPATRP
SSCQRSSQFSQWAVHLTFDLRSISHYLEVSECGRRVIVSSHKQAYTDCPERFRINQVLCSEGFSSGHRYWEVRTE
QGTTWSAGVAMKEPSYLNHYLGRKGLSWCIEWAGRQLAAWHQGEKIPIGKDKPRVLGVFLDLEAGELSFYSVDSQ
ERLLHQFKVDVSAPLFPAFWLYGAESGNSLAFKQDGT
>113_ENSOANG00000010402:ENSOANT00000016497 peptide:ENSOANP00000016494
pep:NOVEL_protein_coding
KAKEEKIESGTRAPKEFLEFPENINQLELLETHRHLIPTGTQSLWEGDSDEEEEQDEKTEEWYQVQENKLKKEPE
KLLLWAAEKNRIATVQRLLAEKATRVNVRDEDEYTPLHRAAYNGHLAIVRELVSQGADVHAVTTDGWTPLHSACR
WDNVSVAAFLLQHEADVNAQTRGLLTPLHLAAGNRDGKNTLELLLMNRYIKPNLKNHAGETALDIARRSSVYHYL
FEIVEGCTNSSLPES
>115_ENSOANG00000015639:ENSOANT00000032408 peptide:ENSOANP00000028612
pep:NOVEL_protein_coding
APGCQNLGLMSRETADALSPGGQVFINQEDANKVLGRAKRANSLFEELKKGNLERECNEETCSYEEAREVFEDTD
KTNEFWNIYKDGNQCETQPCQNQGVCKDGLGEYTCLCSAGYEGKNCDTTTVKICSLNNGDCEQFCKSVVNTVVCS
CAQGYILGDDQKSCIPTVPFPCGKLTVGRRKRSAELPEEQDGDNAHVAEDVLEATENPFEAEPPDQNTTLAEPGE
NALVRIVGGRECHDGECPWQALLVNDENGQGFCGGTILNEYYILSAAHCMHQAKRFKVRVGERDTEKKDSSEMAH
EVEKVIVHSKFVKKTYDFDIAVIKLKTPITFRMNVSPACLPEKDWAEDILMNQKAGVVSGFGRVHEKGRPSTVLK
MLEVPYVERSTCKQSSSFDITPNMFCAGYDSRPEDACQGDSGGPHVTKYKDTYFVTGIVSWGEGCAQNGKFGVYT
KAATFLSWIKRMMRQKAGRSSAPRER
>115B_ENSOANT00000024652 peptide: ENSOANP00000024648
pep:NOVEL_protein_coding
MAGDWRILGLLFLHLLFPQTEQTVFLSPTRANNVMARQKRARSYVIEEVFEGNLERECLEERCDYEEAREVFEDK
AVTDRFWSHYSGGRKCDSNPCQNNGKCQDISYGYICLCPEGYEGINCQHESSKCHPLTRGGCQQFCHPGLRSFKC
SCASGYTLGEDDKSCFPNEEDCACGILNQEGNGTVLETKEARPEFLWQVKLMNSEGEEFCGGVILKKNFVLTTAE
CARTYENISVFLGNATADPTPLDVQVHRIYEHMHYDRETGENNLALLELAEPIRCRDGRVPVCIPENDFASYVLI
PSKLGVISGWTVDGNQLRDWTVGRSDAHLSDKECERAVNGTVTSRMFCVKPSGSATKPLVEGSIITMEDRNTWFL
LGILNSPPTQQPGQSLLFTKVTRYSMWFRKIMG
>new 116 lcl|GenomeScan_predicted_peptide_3|3205_aa:LATA_LATMA-
:715..790:E=6e-11
MRRRWVKESSEEMGVSEPCRGRSFRYVAGSTRDASLASDLCSGVADPIVLEQAGPFPAVENEKISSSSLRNEVVV
STYPEKGPVSGFRSNAYRGELMIPGAESGFIFARVLSPLFKMRRSLVLGCERRRWFPGEYVLPNPRPPRLSLPFR
VTRTYRKPSRGSDAAVPMQVLCSSCPPVPASQRHSRASSAADPVTLLPFPLMSAAGRDRLLEWPILRRKGIIPAL
IFSWRFTARTVNSRPGRRRSFQVEVSGEVKLVIGTVAKVESFILDQDDLENPMLETASKLLLSGTADGADLRTVD
PETQARLEALLEAAGIGKLSTADGKAFADPEVLRRLTSSVSCALDEAAAALTRMRAESTANAGQTDNRSLAEACS

EGDVNAVRKLLIEGRSVNEHTEEGESLLCLACSAGYYELAQVLLAMHANVEDRGIKGDITPLMAAANGGHVKIVT
LLLAHGADVTAQSSTGNTALTYACAGGYVEVVKVLLESGASIEDHNENGHTPLMEAGSAGHVEVARVLLENGAGI
NTHSNEFKESALTLACYKGHLEMVRFLLEAGADQEHKTDEMHTALMEACMDGHVEVARLLLDSGAQVNMPADSFE
SPLTLAACGGHVELAALLIERGANLEEVNDEGYTPLMEAAREGHEEMVALLLGQGANINAQTEETQETALTLACC
GGFLEVADFLIKAGADIELGCSTPLMEAAQEGHLELVKYLLAAGANVHATTATGDTALTYACENGHTDVADVLLQ
AGADLEHESEGGRTPLMKAARAGHVCTVQFLISKGANVNRTTANNDHTVLSLACAGGHLAVVELLLAHGADPTHR
LKDGSTMLIEAAKGGHTSVVCYLLDYPNNLLSAPPPDVTQLTPPSHDLNRAPRVPVQALPMVVPPQEPDKPPANV
ATTLPIRNKATSKQKSSSHLPANSQEAQGYITNQSPESIVEEAQGKLTELEQRIKEAIEKNAQLQSLELAHADQL
TKEKIEELNKTREEQIQKKQKILEELQKVERELQLKTQQQLKKQYLEVKAQRIQLQQQQSCQHLGLLTPVGFGEQ
LPEGDCARLQQAEPISLKDDPQQTAAQMGFAPIQPLAMPQALPLATGSLPPGSIANLTELQGVIVGQPVLGQAQL
AGLGPGILTETQQGLMVASPAQTLNDTLDDIMAEGSSVSGLQCQQEFCPTWLFHTSLSTLPSAVSGRASTMSNTP
THSIATSISQPQTPAPSPIISPSAMLPIYPAIDIDAQTESNHDTALTLACAGGHEELVQTLLERGASIEHRDKKG
FTPLILAATAGHVGVVEILLDNGADIEAQSERTKDTPLSLACSGGRQEAISVTQSGLGWGSLGKGTVGVEARALT
AAMAQEKPKNWSMGGLGEKGGGLGVVELLLTRGANKEHRNVSDYTPLSLAASGGYVNIIKILLNAGPREECEGGA
DQVALGNDHLFKAGTGLLLTDGPDSEGGGGCCSGNGNGIHVSLSGWKSEKQRGLVDPAYYRQGRLLMPSNASLLG
KTGSKLGISPLMLAAMNGHTAAVKLLLDMGSDINAQIETNRNTALTLACFQGRTEVVSLLLDRKANVEHRAKVVE
LLLTRGANKEHRNVSDYTPLSLAASGGYVNIIKILLNAAMNGHTAAVKLLLDMGSDINAQIETNRNTALTLACFQ
GRTEVVSLLLDRKANVEHRAKTGLTPLMEAASGGYAEVGRVLLDKGADVNAPPVPSSRDTALTIAADKGHYKFCE
LLISRGAHIDVRNKKGNTPLWLAANGGHLDVVQLLVQAGADVDAADNRKITPLMAAFRKGHVKVVRYLVKEVNQF
PSDSECMRYIATITDKEMLKKCHLCMESIVQAKDRQAAEANKNASILLEELDLEKVKLFPQCKNAFADLIELFLF
VELVVNLSRFDVDSRGPNYLGGRLEELDSGLQRLTVKSTVPFLVGLTPLGGNNNNEHHHRVNNDGTCSTLIKFQL
REESRRLALAAKREKRKEKRRKKKEEQRRKLEEIEAKNKENFELQAAQEKEKLKVEDEPEAPTEPPSATTTTTIG
ISATWTTLAGSHGKRNTTITTASSKRKSRKNKIGPENVQILFEDQLPISYGQPEKVNGESKSSSTSESGDSDNMR
ISSCSDESSNSNGSRKSDGPAPAAGPQPAKKQPAVLVTFPKEERKPASAKPSVKLVSVGRPGTAAPGRVPARSRL
GGTRFPRPRNPSRSARSGISAGPQIPDAAAMAWFLLAEDEGIEERRGLVARAQGWERLTEVIGEATTNSLSTCTK
SGPSPLSSPNGKLTVASPKRGQKREEGWKEVVRRSKKVSVPSTVISRVIGRGGCNINAIREFTGAHIDIDKQKDK
TGDRIITIRQKPHSRWVAFFLPALQGCSLALAHALLAAQTFQQIRPPRLPMTHFGGTFPPAQSTWGPFPVRPLSP
ARATNSPKPPPVPRHGAQNGGGPQANAAGPAPAGPAPAAAPAVSAPAAGGPGSPSRQRPPRLPDICSPRPPQTMP
GPLLENSTPCPPRTPFPRQGTWGGCRFYPDCNENLGFLSNHTNSRPLDWNPSVAVLNVNHVKRPHSVPSSVQLPS
TLSTQSASQNPAHPAGKSMAPNFSAPLPFGPFSTLFENGPNATHGFWGGSVVSSQSTPESMLSGKSSFLPNSEPL
HQSDTSKAPGFRPPLQRPAPVPSGLVSMDSPYAPVPPSSTHLGNFASNLSGGPMYAPGAPLGGAPAAANFNRQHF
SPLSLLTPCSSASNDSPAQSVSSGVRAQSPAQSAVSLGSEKPSSVSQDRKVPVPIGTERSARIRQTGTSTPSVIG
SNLATPVGHSGIWSFEGIGGNQDKVDWCHSGMGNHMIHRPMSDPGVFSQHQAMERDSTGIVTPGTFHQHVPAGYM
DFPKVGGMPFSVYGNAMIPPVAPIADGAGGPIFNGPHAADPAWNSLIKMVSNSTENNGPQTNGARPIVKRLNANI
VIIIKRFFSELRAGCRPAAVREREEACGLHFTAEEKGTEKVENLQPEIKNIAKYLNHEISLRAKRLGWSKRIARL
THMNNMRRLVSELYVRHNCHPFKATLLVWIQLPMWVVVSVALRNLSVGAVDSEGLPIREQLSTGGALWFPDLTVL
DSTLILPVSLGILNLLIVEVFALQKLEMSRFQKYLTHFIRGVSVLMIPIAATVPSSIALYWLCSSCLGLSQNLLL
RSPRFRRLCRIPPARSDSDTPYTDLRAAFYAKYLPGKRSGRREPGSGGPAPGRSE
>117_ENSOANG00000014706:ENSOANT00000023178 peptide:ENSOANP00000023174
pep:NOVEL_protein_coding
SLKVMKLRKLAQQVANCRQCLERSTVLINQAEHILKENDHARFLQTAKNVAERVAMATASSQVLIPDINFNDAFE
NFALDFSREKKLLEGLDYLTAPNPPSVREELCTASHDTITVHWISEDEFSVGSYELQYTIFTGQANFISLYSSMD
SWMIVPNIKQNHYTVHGLQSGTRYIFLVKAINQAGSRNSEPARLKTNSQPFKLDPKLTHKKLKISNDGLQMEKDE
SSLKKSHTPERFSGTGCYGAAGNVFIDSGCHYWEVVVGSSTWYAVGVAYKSAPKNEWIGKNASSWVFSRCNNNFV
VRHNNKEMLVDVHPQMKRLGVLLDYDNNTLSFYDPANSLHLHTFDISFILPVCPTFTIWNKSLMILSGLPAPDFI
DYPEQQE
>118_ENSOANG00000001493:ENSOANT00000002374 peptide:ENSOANP00000002373
pep:NOVEL_protein_coding
VLKTTSELFLSSAAEGADLRTVDPETQARLEALLEAAGIGKLSTADGKAFADPEVLRRLTSSVSCALDEAAAALT
RMRAENSQNAGQADNRSLAEACSDGDVNAVRKLLDEGRSVNEHTEEGESLLCLACSAGYYELAQVLLAMHANVED
RGNKGDITPLMAAASGGYVDIVKLLLVHCADVNAQSSTVQGNTALTYACAGGFVDIVKELLKAGANIEDHNENGH
TPLMEAASAGHVEVARVLLEYGAGINTHSNEFKESALTLACYKGHLDMVRFLLEAGADQEHKTDEMHTALMEACM
DGHVEVARLLLDSGAQVNMPADSFESPLTLAACGGHVELAALLIERGANLEEVNDEGYTPLMEAAREGHEEMVAL
LLAQERNINAQTEETQEALTLACCGGFSEVADFLIKAGADIELGCSTPLMEAAQEGHLELVKYLLAAGANVHATT
ATGDTALTYACENGHTDVADVLLQAGADLEHESEGGRTPLMKAARAGHLCTVQFLISKGANVNRATANNDHTVVS
LACAGGHLAVVELLLAHGADPTHRLKDGSTMLIEAAKGGHTNVVSYLLDYPNNVLSVPAADMSQLTPPSQDQSQV
PRVPVHALAMVVPPQEPDRAPQENSPSLLGLQKAVSSRVPAGPNSSSHTTESPTPEPCSQTPATAASQSVLPMYP
SVDIDAHTESNHDTALTLACAGGHEELVSVLIAREAKIEHRDKKGFTPLILAATAGHVGVVEILLDKGGDIEAQS
ERTKDTPLSLACSGGRQEVVDLLLARGANKEHRNVSDYTPLSLAASGGYVNIIKILLNAGAEINSRTGSKLGISP
LMLAAMNGHVPAVKLLLDMGSDINAQIETNRNTALTLACFQGRAEVVSLLLDRKANVEHRAKTGLTPLMEAASGG
YAEVGRVLLDKGADVNAPPVPSSRDTALTIAADKGHYKFCELLINRGAHIDVRNKKGNTPLWLAANGGHFDVVQL

LVQAGADVDAADNRKITPLMSAFRKGHVKVVQYLVKEVNQFPSDIECMRYIATITDKELLKKCHQCVETIVKAKD
QQAAEANKNASILLKELDLEKSREESRKQALAAKREEDEENKPKENLELPEDDDEEENDDEVEPEVPIEPPSATT
TTTIGISATSTTFTNVFGKKRANVVTTPSTNRKNKKNKTKDTPQNVQLILPDQHISLAQQKADKNKITGDSDSDN
LDSTDCNSESSSSGGKSQELNFTMDVNSSGERRYASLLLHSHEEKPGANAATAKAPTRLESEANPGSLSTNYKSV
SLPLSSPNLKLNLTSPKRGQKREEGWKEVVRR
>119_ENSOANG00000002928:ENSOANT00000004654 peptide:ENSOANP00000004653
pep:NOVEL_protein_coding
SEISHSICKLPYSVGPCKSSITMYYFNAKANQCKQFIFGGCWGNENRFSTLDECKKACDEHGKNARVPDSETSSS
ICQLSYSVGLCKARIPQYHFDSNTQQCKRFYYGGCQGNENRFSIKAECRNTCAEPISHPTSGHGSLRRKSITQVT
RLLVSPQLCPTPTAWFLCSGNICEKPMDKGLCMAYMPKFYYNSKTKKCESFIYGGCQGNENRFDTVEECMARC
>120_3|254_aa:KC1_ANESU-:5..56:E=2e-20
MGSNLCSVTWQLYDFGHQKEGVDTNAGETVPALRHKNAAAVDVAKSVLQSFHRHLIQCLAHTSGSICEKPVEVGR
CMGHMPMYYYNSKKKKCEPFIYGGCQGNENRFKTMDLCKSHCGGSTKSGRCPPPPKGYRGSCYESCKGDQYCPPK
HKCCSNGCGRSCKLAVTDILWGSRRAFTLKGRPSPVGPGSRYEASWTPAPLPGQRQHLLWRRGKVQTKTGMGERK
LDSTGYGGHLEDIRCFGPSVFNQSVNRVY
>new 122 lcl|GenomeScan_predicted_peptide_6|878_aa:KC1_ANESU-:5..56:E=3e-21
MPRLHLRSFWPPDSVPTASGAPGPETLVLPLPVPWILDCGHPVPGSPDLEIPFLPILTPRTWRIQSSRSRCLEPA
CGRLTGRPRSWTLTTPLVSFRDGENRGETRDMSNSTEDISQALCGTLQRRQFLPSGIQVLQQRMRSRTGHSGKRN
VGLGAEEECEWELIGGRGKEECRGEPSTDPTNRTSLEIPPGSFREGFRDLVPAQPRHQEQKEVRIVYAAAGETLP
ALRGKSVAALDAAKFARHRTVWAGQESVGSRAQLGRTARAGQESVERRAEREKGLRDPAPAHPCLRVRKGPAYNS
AEEMIPALRDRCAAPMDVTLTAHQRWKRGLRDPAPAHPCLRVRKGPAYNSAEEMIPALRDRCAAPMDVTLTARHR
WKRGLRDPAPAHPCLRVRKGPAYNSAEEMIPALRDRCAAPMDVTLTAHQRWKRGLRDPAPAHPCLRVRKGPAYNS
AEEMIPALRDRCAAPMDVTLTARHRWKRLHYYPSAEDPTQQRPAYSPKSLVQCSVYGSKNVCEKSADPGPCKSYM
AVYYFNSRTKNCEAFMYGGCGGNDNRFRTAEECMARCGGSKNPVVCSTPSPGTKGNCDQDCRGDNSCGKRQRCCA
QGCGQVCDAPVKGGQLEPGRKAWRVGLSGAGSRDICEEPVEVGRCMGHIPMYYYNSKTKKCESFIYGGCQGNENQ
FKTIEECMARCGGSVKLGRCPISRKEISEPCVEECKGDDSCPVGQKCCVSGCSQVCMLSVKDGMAARQFTEAGDL
RLGGPVQGPSGGQRECGQRFQGSFPWSSGPLGDVRSQGSAPASSHQGGDTKPLGLQPHYKGSGDNSHGGEEKCKL
KLARMIFGPMRDGDYVQPNLFGEKVVRSYKPLMEKASERREQSQVVIVMLGSA
>new 124_2|1132_aa:gi|73621141|sp|Q696W1.1|LC2_VIPLE-:27..70:E=3e-05
MSTLESSNSADPHFLVTLRKGASPNNWEQTDSLFPPWRLIAGTLSVLCLGLVATVATMALRVFKRNWVQFSMSCY
QYSAEKEPWKESQQACKSQNSTLLHIDGLRELNFFKFFQLSGWIGLSRTGPDSSLKWEDETTYTRNFLSFTKERK
GGDCALYLNEKSISMEDCAIGKLHICERQARISSCLQDVSTWMSARHLKLNMSKTELLIFPPKPGPLPDFSITMD
GTTILPVSQARNLDFPFSPWRILAVTLGMLFLVLLVSVQLLQAKQMSDQIFYTELKKKPWSQPQPKRPKNNKSKD
SGKEKELNYVELNLHRSSQQDSKQGRLKDKEPSEEQVTYPELNKERLDLPWHGQKSKGTKGRRSAGEESVTYMDL
KHHNRPQWCSPFENAHSKVLSNQELNNTSSSHSNTTSLNGYHSGPCPVNWVNYRNSCYLISMERKTWQDSHMICV
SQNSSLLKIDSMEELTVMFLKFFPDYGFLKSLKSVQFRILPTREMEKHQVIYSELHIHNSSQKPDGRPQKHKSKF
VSSEQQVTYAEMKNSKLAQQQHRKSKNFPDEDQRSRQPKQKRYKDVKNTGLESDQQVTYTDLKLLSQPQLQGRCE
VGQSKGNWVQFSTGCYQYSAEKKPWKESQQACKSQNATLLHIDGLRELVYVHTLFIDINAFPDHLQDDKSSQWTF
GRRNRLSLFPLADLRGDSGAAFAGATCVHPASARKMNWCHGRLRPSTARWVPGFPFPLVSSSHKSEPWTKYPQNI
FSKQSLVQYSAHSKRSTNSIDSLISYYSWLVYWFGTFSGDGDQMSEQQVFYTELKINNFSQPHSKRQGDKKSKDS
GKEKELNYAELKLPRSSQQESKQERLDDRACGGSGNDRKIFSSPETSEQQVTYSELNEQRLNLSWQDQKSKGAKS
RRSTNEQSVTYSELKQHNRPQQCSPSENAQSKVTQQQEVSPSPLPKPSHNTISKTGCDWDPCPGDWIGFRNSCYL
FSNDTKNWRDSKIACATLNSSLLWLDNQEELKFSQRGVSEAICTDELSEREFQQDKYGTQSRPVQATGSTQTSHL
AVIEISHSPALDQPHREGGSSGAEMAGEMSERQVFYMELKKNNNLSQTHPQRPKNNKNKDSGKEKELSYVELKLH
SSQQESK
>125_ENSOANG00000003664:ENSOANT00000005815 peptide:ENSOANP00000005813
pep:NOVEL_protein_coding
ETFAVSLESGELPSEGIVRVKLVNGDEQAYLCNSKWSITEANVVCRHLGHAQGALHHRYANDSAIRMKAPHCLDV
TCRGLESSLSECAFSKRADTHDETPAGVTCYLREVEPPSPDSFECVNGKFVSRSKTCDGINDCGDQSDELCCRAC
RPGGFHCTSDVCIPEDHRCDGEMDCILGDDENNCKGNSELRNCILSPVSISRAVSKLDMNIERRRIKSLIPQITC
GMRRNISTRRKRILGGKAAVKDEFPWQVAIKEDNQIKCGGIYIGGCWILTAAHCVRQNRAHRYQVWTGLLDWIKL
NPEIQIHRVNQVIVHDQYSASTYQNDIALLEVKKQGNKKECNLPFTIPACLPWSEYMFRPNHRCVVSGFGLEEEF
ARVYSLKWGHVNLIANCSKFYSSRYHEKEMLCAGKYSGISSSRRCPSETRISHSPGSVARAYLWGVVSWGENCGK
PQFPGVYTKVAHYFDWISRHVGRSLVSRYNV
>126_ENSOANG00000014944:ENSOANT00000023530 peptide:ENSOANP00000023526
pep:NOVEL_protein_coding
GSVRLRGGKNKYEGTVEVYSNKVWGTICSSQWDDNGAAVVCRQLQLGERGIAKQTPFSGLGLIPISWSSVRCRGD
EENILLCEKDVWQDGTCPQKMAAAVTCNFSGGPAFPPVRLVGGRSSHEGRVEVYHGGQWGTVCDDQWDDADAEVI
CRQLSLGFAHVNSGVAKAWSQAYFGEGSGPVLLDEVRCTGNELSIEQCSKSSWGEHNCGHKEDAGVSCTPLTDGA
IRLAGGKGSHEGRLEVRYSGQWGTVCDDGWTELNTQVVCRQLGFKFGKLVPESRFEESTGPILLDDVSCSGKESS
FLQCSRREWGKHDCSHREDIGMTCHPDNDVHRLSLGLPIRLMDGENKKEGRVEIFINGQWGTICDDGWTDKDAAV

VCRQLGYKGPSRARTMAYFGEGKGPIHVDNVKCAGNERSLADCIKQDIGKHNCRHSEDAGVICDYFGKKALGNSN
TDSLSSVCGLRLLQRRQKRIIGGKNSLRGGWPWQVALRLKSSHGDGRLLCGATLLSSCWVLTAAHCFKRYGDNTR
NYAVRVGDYHTLVPEEYEEEIGVRQIVIHRDYRPDSSDYDIALVRLQGPEERCAKFSTHVLPACLPLRRERPQKT
APNCYVTGWGDTGRAYSRTLQQAAIPLLPKRVCEERYKSRFTGRMLCAGSLLEQKRVDSCQGDSGGPLMCERPGE
SWVVYGVTSWGYGCGIKDSPGVYTKVSAFVAWIKNMTKL
>127_ENSOANG00000002628:ENSOANT00000004184 peptide:ENSOANP00000004183
pep:NOVEL_protein_coding
METLESELTCPICLELFEDPLLLPCAHSLCFNCAHRILVSHCASNECVDSITAFQCPTCRYVITLNQRGLDGLKR
NVTLQNIIDRFQKASVSGPNSPSETRRERASDGGSMTSSEKVLCQFCDQDPAQDAVKTCVTCEVSYCEECLKATH
PNKKPFTGHRLIEPIPDSHIRGLMCLEHEDEKVNMYCVTDDQLICALCKLVGRHRDHQVAALSERYDKLKQNLES
NLTNLIKRNTELETLLAKLIQTCQHVEVNASRQETKLMEECDLLIEIIQQRRQIIGTKIKEGKVVRLRKLAQQIA
NCKQCIERSTSLISQAEQSLKENDHARFLQTAKNITERVSMATASSQVLIPEINLNDTFDTFALDFSREKKLLEC
LDYLTAPNPPTIREELCTASYDTITVHWTSDDEFSVVSYELQYTIFTGQANVVSLCNSADSWMIVPNIKQNHYTV
HGLQSGTKYIFIVKAINQAGSRNSEPGKLKTNSQPFKLDPKSAHRKLKVSHDNLTVERDETSSKKSHTPERFTSQ
GSYGVAGNVFIDSGRHYWEVVISGSTWYAIGLAYKSAPKHEWIGKNSASWVLCRCNNNWVVRHNSKEIPIEPAPH
LRRVGILLDYDNGSVAFYDALNSLHLYTFDITFAQPVCPTFTVWNKCLTIITGLPIPDHLDCTEQLP
>130_ENSOANG00000010179:ENSOANT00000016139 peptide:ENSOANP00000016136
pep:NOVEL_protein_coding
MTFSGLQLVWEELTCSVCMEYFVDPVTLSCGHSFCHPCLLKSWEETFQVSSCPECRGAFEPGDLQINQRLGKLAL
IGKQLRPCLLQQSSTGEGLLCGEHQQTLKLFCQEDKLPMCVACCHSEVHADHRVSPIDEAAEDYRERLQELLRHL
WTEMAQVQKLISKEKKNSEYLEEEVERWKQNLVSEFEKMHRLLDKELELKLERLEKEAAENRKTLWQNKEKLSQQ
TRKLRALITEIEEKCQRGDTELLQVRLLEREGEEDRGMGNVEQTSPGTPLSVCCVPGMREMLSRYEVDVTLDPDT
ASPYVIVSPDRKSVKFVETRQNVPDHEGRFDNCASVLGAAVFTSGRHYWEVEVGDKPEWEVAVCKESRNRKNSMP
IFPGDTFSLMTFQTKRGASLWVSSPLIPLSMKMPTHRLGVFLDYEAGVVSFYNVMEKCLIYSFPPTRFSGPLRPV
FSPCLVHRGQNTEPLTIRPV
>131_ENSOANT00000011456 peptide: ENSOANP00000011454
pep:NOVEL_protein_coding
MDYFQMIFSLLFVVFQGALETAVLGADLSTGIGTGVEVHPPPAPWRPRRTKRCSCSSLLDKECVYFCHLDIIWIN
TPEHTVPYGLGGPSRSKRALQDSFPAKQSDGNNRCQCANQKDKKCWDFCQAGKELWAQNTLEKGRKQLKKGEQCA
DLGLKCVYWQLVNRRKMRRMEAIGNRIKAAFNFAKLKAELHMAKKVTHNRAH
>132_2|159_aa:IVBTI_OXYSC-:4..58:E=1e-20
MSICSWKRITETPKNPGEDGVPVRRYYYDPAAARCLPFNYSGCGGNKNRFNNRKKCLAVCGIPGIPPICQLPRKK
GPCNQELPRFYFNTATRTCELFSYGGCEGFLNRFQTEEECLKTCLSDVYLKEQHNGKSMGLGDEDLDSDSSSATF
LLCDFGQVT
>133_ENSOANG00000004023:ENSOANT00000006376 peptide:ENSOANP00000006374
pep:NOVEL_protein_coding
ACERDVQCGAGTCCAISLWLRGLRMCTPLGQEGDECHPPTRKVPFFGKRQHHTCPCLPNLLCSKFLDGRYRCSVD
F
>134_ENSOANG00000003965:ENSOANT00000006275 peptide:ENSOANP00000006273
pep:NOVEL_protein_coding
PLTVIKFLEVYERSFCRTIETLVDIFQEYPDEVEYIFKPSCVPLMRCAGCCNDEGLECVPSEVHNVTMQIMRIKP
HHSQQINEMSFQQHSKCECRPKKDVKAKQEKKSKRGKGKGQKRKRKKARYKSRSFPCEPCSERRKHLFVQDLQTC
KCSCKNTDSRCKSRQLELNERTCRCDKPRR
>new 136_2|142_aa:HELO_HELHO-:200..242:E=2e-10
MKREHSRPDARRGREVALVIPLGRGCVGREGCSAPVSTTGGVEKAWSDGKIPRLGVIEDLRGSVERARALEGNYR
AKINKPYQRGTPCGSCKNACDKGLCTNPCKYENKWSNCEELKNFATCNHPQVKSDCEASCKCTTEIK
>142_ENSOANG00000013321:ENSOANT00000021035 peptide: ENSOANP00000021032
pep:NOVEL_protein_coding
YLPIPTLMARLCLLLAAVWHTAAGAPRPEMFGRLTSPGFPAVYPNNKEKSWQLAAPPGHVIKIYFTHFNLELSYL
CEYDYVKLRSGDKELVTLCGQESTDTEQAPGNRTFRSIGNSLVVTFRSDYSNEKPFTGFEAFYAAEDVDECATLP
GREPACDHHCHNYVGGFYCSCSLGYTMHRDKRTCSAKCTGLVLSERSGVITSPDYPKGYPKLSNCSYSIRVEDGF
SIILEFVESFDVETHAEVLCPYDTLKIKTDKREYGPFCGQTLPPRIETGSNTVEIIFITDVSGDHTGWKIKYITT
GLSCLKPGAPPNGYISPLQQEYTVKDHFSLSCLKGYVLLQGDKILKSFTATCQKDRSWNQPMPKCVVVDCGPPAD
IPSGRVSYITGPEVTTYEAEIQYSCKIPFYTLKTSNDGKYHCGADGFWKSSRGEKSPPVCEPVCGISTRTAVERI
FGGKMAKFGEFPWQVRLRGERFGGGALLYDNWVLTAAHVVYGHKDLSSLVIRMGALKRLSPNYIQAWAEAVFIHE
DYLHDNVNFNNDIALIKLKHRIEINGNITPICLPGRDSRFHLKPNDLGTVSGWGRTENRPLASSLTYVEVPVVDT
QTCKNAYAKKKEVTKFLLTDNMICAGFESGGKDACAGDSGGPLVFLDSETKKWFVGGIVSWGLQCGVAEQYGVYT
NVNNYISWIENIILNN
>143_ENSOANG00000006443:ENSOANT00000010286 peptide: ENSOANP00000010284
pep:NOVEL_protein_coding

KESSGSPGEMGLLRAFDSAEFVSWEKIGSGGFGQVYKVRHVHWKTWLAIKCSPGLHVDDKDRVELLEEAKKMEMA
KFRYILPVYGICRDPVGLVMEYMETGSLEKLLASEPLPWELRFRIIHETAVGMNFLHCMSPPLLHLDLKPANILL
DAHYHVKISDFGLAKCSGLSHSHDLSMDGLCGTIAYLPPERIKEKNRFFDTKHDVYSFAIVIWGVLTQKKPFADE
KNILHIMVKVVKGHRPELPPVSKSRPRSCNNLIRLMQKCWRDNPRERPTFQGDNIKEIQVGFKYSEIIIISQLNW
GILALKKQLNTTVTANVTSPDLPQEITSETEDLCEKPDDEIKETQELDIKNPHEPKTEEMPALPAPKRASAPAFD
ADYSLSELLSQLDSETSQTMEGPDELSRSSSESKLVSTSSGKRLSGVSSVDSAFSSRGSLSLSFERENSVSDIST
TDLHKRKLAEAITSGDTGKLMKILQPQDVDLVLDGQSSLLHLAVEAGQEECVKWLLLNNANPNLTNKRGSTPLHV
AIEKKVRSVVELILARKINVNAKDEDQWTALHFAAQNGDECSTRLLLEKNASPSEVDFEGRTPMHVACQHGQENI
VRILLRRGVDVSPQGKDDWLPLHYAAWQGHLPIVKLLAKQPGVSVNAQTLDGRTPLHMAAQRGHYRVARILIDLR
SDVNVRNRLLQTPLHVAAETGHTSTSRLLLNRGAEKEALTVEGCTALHLASRNGHLATAKLLLEEKADVLGAGPL
SRTALHLAAANGHAGVVEELLGAVAIDAADREGLTALHLAVRGGHAETMEVLLKHGARINLQSLGAQAPPLLPGN
SPVATLLRRRNT
